# Supplementary material for: A Rational Synthesis of a Branched Decaarabinofuranoside Related to the Fragments of Mycobacterial Polysaccharides
Source: Molecules. 2025 Aug 6;30(15):3295. doi: 10.3390/molecules30153295 (PMC12348351; doi:10.3390/molecules30153295)

**Supplementary data**

# A Rational Synthesis of a Branched Decaarabinofuranoside Related to the Polysaccharide Fragments of Mycobacteria

**Polina I. Abronina[1]*, Nelly N. Malysheva[1], Maxim Y. Karpenko[1], Dmitry S. Novikov[1], Alexander I. Zinin[1], N. G. Kolotyrkina[1], Leonid O. Kononov[1]***

N. D. Zelinsky Institute of Organic Chemistry of the Russian Academy of Sciences, Leninsky prosp., 47, Moscow 119991, Russian Federation.

e-mail: polina-abronina@yandex.ru, leonid.kononov@gmail.com

## Table of Contents

**4-(2-Chloroethoxy)phenyl 2,3,5-tris-*O*-(triisopropylsilyl)-β-D-arabinofuranosyl-(1→2)-3,5-bis-*O*-(triisopropylsilyl)-α-D-arabinofuranosyl-(1→3)-[2,3,5-tris-*O*-(triisopropylsilyl)-β-D-arabinofuranosyl-(1→2)-3,5-bis-*O*-(triisopropylsilyl)-α-D-arabinofuranosyl-(1→5)]-2-*O*-benzoyl-α-D-arabinofuranosyl-(1→5)-2,3-di-*O*-benzoyl-α-D-arabinofuranoside (2)**


$^{1}$H NMR spectrum of compound **2** S6

$^{13}$C NMR spectrum of compound **2** S7

COSY $^{1}$H-$^{1}$H spectrum of compound **2** S8

HSQC $^{1}$H-$^{13}$C spectrum of compound **2** S9

HMBC $^{1}$H-$^{13}$C spectrum of compound **2** S10

HMBC $^{1}$H-$^{29}$Si spectrum of compound **2** S11

$^{29}$Si INEPT NMR spectrum of compound **2** S12


**2,3,5-Tris-*O*-(triisopropylsilyl)-β-D-arabinofuranosyl-(1→2)-3,5-bis-*O*-(triisopropylsilyl)-α-D-arabinofuranosyl-(1→3)-[2,3,5-tris-*O*-(triisopropylsilyl)-β-D-arabinofuranosyl-(1→2)-3,5-bis-*O*-(triisopropylsilyl)-α-D-arabinofuranosyl-(1→5)]-2-*O*-benzoyl-α-D-arabinofuranosyl-(1→5)-2,3-di-*O*-benzoyl-D-arabinofuranosyl *N*-phenyltrifluoroacetimidate (4)**


$^{1}$H NMR spectrum of compound **4** S13

$^{13}$C NMR spectrum of compound **4** S14

COSY $^{1}$H-$^{1}$H spectrum of compound **4** S15

HSQC $^{1}$H-$^{13}$C spectrum of compound **4** S16

$^{29}$Si INEPT NMR spectrum of compound **4** S17

$^{19}$F NMR spectrum of compound **4** S18


**4-(2-Azidoethoxy)phenyl β-D-arabinofuranosyl-(1→2)-α-D-arabinofuranosyl-(1→3)-[β-D-arabinofuranosyl-(1→2)-α-D-arabinofuranosyl-(1→5)]-α-D-arabinofuranosyl-(1→5)-α-D-arabinofuranosyl-(1→5)-α-D-arabinofuranosyl-(1→5)-α-D-arabinofuranosyl-(1→5)-α-D-arabinofuranosyl-(1→5)-α-D-arabinofuranoside (5)**


$^{1}$H NMR spectrum of compound **5** S19

$^{13}$C NMR spectrum of compound **5** S20

COSY $^{1}$H-$^{1}$H spectrum of compound **5** S21

HSQC $^{1}$H-$^{13}$C spectrum of compound **5** S22

HMBC $^{1}$H-$^{13}$C spectrum of compound **5** S23


**2,3,5-Tris-*O*-(triisopropylsilyl)-β-D-arabinofuranosyl-(1→2)-1-*O*-acetyl-3,5-bis-*O*-(triisopropylsilyl)-α-D-arabinofuranose (10)**


$^{1}$H NMR spectrum of compound **10** S24

$^{13}$C NMR spectrum of compound **10** S25
COSY $^{1}$H-$^{1}$H spectrum of compound **10** S26
HSQC $^{1}$H-$^{13}$C spectrum of compound **10** S27
HMBC $^{1}$H-$^{13}$C spectrum of compound **10** S28
HMBC $^{1}$H-$^{29}$Si spectrum of compound **10** S29
$^{29}$Si INEPT NMR spectrum of compound **10** S30

**4-(3-Chloropropoxy)phenyl 2,3,5-tris-*O*-(triisopropylsilyl)-β-D-arabinofuranosyl-(1→2)-3,5-bis-*O*-(triisopropylsilyl)-α-D-arabinofuranoside (13)**

$^{1}$H NMR spectrum of compound **13** S31
$^{13}$C NMR spectrum of compound **13** S32
COSY $^{1}$H-$^{1}$H spectrum of compound **13** S33
HSQC $^{1}$H-$^{13}$C spectrum of compound **13** S34
HMBC $^{1}$H-$^{13}$C spectrum of compound **13** S35
HMBC $^{1}$H-$^{29}$Si spectrum of compound **13** S36
$^{29}$Si INEPT NMR spectrum of compound **13** S37

**4-(2-Chloroethoxy)phenyl 2,3,5-tris-*O*-(triisopropylsilyl)-β-D-arabinofuranosyl-(1→2)-3,5-bis-*O*-(triisopropylsilyl)-α-D-arabinofuranosyl-(1→5)-2-*O*-benzoyl-α-D-arabinofuranosyl-(1→5)-2,3-di-*O*-benzoyl-α-D-arabinofuranoside (14)**

$^{1}$H NMR spectrum of compound **14** S38
$^{13}$C NMR spectrum of compound **14** S39
COSY $^{1}$H-$^{1}$H spectrum of compound **14** S40
HSQC $^{1}$H-$^{13}$C spectrum of compound **14** S41
HMBC $^{1}$H-$^{13}$C spectrum of compound **14** S42

**2,3,5-Tris-*O*-(triisopropylsilyl)-β-D-arabinofuranosyl-(1→2)-3,5-bis-*O*-(triisopropylsilyl)-α-D-arabinofuranosyl-(1→3)-[2,3,5-tris-*O*-(triisopropylsilyl)-β-D-arabinofuranosyl-(1→2)-3,5-bis-*O*-(triisopropylsilyl)-α-D-arabinofuranosyl-(1→5)]-2-*O*-benzoyl-α-D-arabinofuranosyl-(1→5)-2,3-di-*O*-benzoyl-D-arabinofuranose (15) and 2,3,5-tris-*O*-(triisopropylsilyl)-β-D-arabinofuranosyl-(1→2)-3,5-bis-*O*-(triisopropylsilyl)-α-D-arabinofuranosyl-(1→3)-[2,3,5-tris-*O*-(triisopropylsilyl)-β-D-arabinofuranosyl-(1→2)-3,5-bis-*O*-(triisopropylsilyl)-α-D-arabinofuranosyl-(1→5)]-2-*O*-benzoyl-α-D-arabinofuranosyl-(1→5)-1,3-di-*O*-benzoyl-β-D-arabinofuranose (16)**

$^{1}$H NMR spectrum of compounds **15+16** S43
$^{13}$C NMR spectrum of compounds **15+16** S44
COSY $^{1}$H-$^{1}$H spectrum of compounds **15+16** S45

HSQC $^{1}$H-$^{13}$C spectrum of compounds **15+16** S46
HMBC $^{1}$H-$^{13}$C spectrum of compounds **15+16** S47

**4-(2-Chloroethoxy)phenyl 2,3,5-tris-*O*-(triisopropylsilyl)-β-D-arabinofuranosyl-(1→2)-3,5-bis-*O*-(triisopropylsilyl)-α-D-arabinofuranosyl-(1→3)-[2,3,5-tris-*O*-(triisopropylsilyl)-β-D-arabinofuranosyl-(1→2)-3,5-bis-*O*-(triisopropylsilyl)-α-D-arabinofuranosyl-(1→5)]-2-*O*-benzoyl-α-D-arabinofuranosyl-(1→5)-2,3-di-*O*-benzoyl-α-D-arabinofuranosyl-(1→5)-2,3-di-*O*-benzoyl-α-D-arabinofuranosyl-(1→5)-2,3-di-*O*-benzoyl-α-D-arabinofuranosyl-(1→5)-2,3-di-*O*-benzoyl-α-D-arabinofuranosyl-(1→5)-2,3-di-*O*-benzoyl-α-D-arabinofuranoside (18)**

$^{1}$H NMR spectrum of compound **18** S48
$^{13}$C NMR spectrum of compound **18** S49
COSY $^{1}$H-$^{1}$H spectrum of compound **18** S50
HSQC $^{1}$H-$^{13}$C spectrum of compound **18** S51
HMBC $^{1}$H-$^{13}$C spectrum of compound **18** S52
HMBC $^{1}$H-$^{29}$Si spectrum of compound **18** S53
$^{29}$Si INEPT NMR spectrum of compound **18** S54

**4-(2-Azidoethoxy)phenyl 2,3,5-tris-*O*-(triisopropylsilyl)-β-D-arabinofuranosyl-(1→2)-3,5-bis-*O*-(triisopropylsilyl)-α-D-arabinofuranosyl-(1→3)-[2,3,5-tris-*O*-(triisopropylsilyl)-β-D-arabinofuranosyl-(1→2)-3,5-bis-*O*-(triisopropylsilyl)-α-D-arabinofuranosyl-(1→5)]-2-*O*-benzoyl-α-D-arabinofuranosyl-(1→5)-2,3-di-*O*-benzoyl-α-D-arabinofuranosyl-(1→5)-2,3-di-*O*-benzoyl-α-D-arabinofuranosyl-(1→5)-2,3-di-*O*-benzoyl-α-D-arabinofuranosyl-(1→5)-2,3-di-*O*-benzoyl-α-D-arabinofuranosyl-(1→5)-2,3-di-*O*-benzoyl-α-D-arabinofuranoside (19)**

$^{1}$H NMR spectrum of compound **19** S55
$^{13}$C NMR spectrum of compound **19** S56
COSY $^{1}$H-$^{1}$H spectrum of compound **19** S57
HSQC $^{1}$H-$^{13}$C spectrum of compound **19** S58
HMBC $^{1}$H-$^{13}$C spectrum of compound **19** S59
HMBC $^{1}$H-$^{29}$Si spectrum of compound **19** S60
$^{29}$Si INEPT NMR spectrum of compound **19** S61

**4-(2-Azidoethoxy)phenyl 2,3,5-tri-*O*-acetyl-β-D-arabinofuranosyl-(1→2)-3,5-di-*O*-acetyl-α-D-arabinofuranosyl-(1→3)-[2,3,5-tri-*O*-acetyl-β-D-arabinofuranosyl-(1→2)-3,5-di-*O*-acetyl-α-D-arabinofuranosyl-(1→5)]-2-*O*-acetyl-α-D-arabinofuranosyl-(1→5)-2,3-di-*O*-acetyl-α-D-arabinofuranosyl-(1→5)-2,3-di-*O*-benzoyl-α-D-arabinofuranosyl-(1→5)-2,3-di-*O*-acetyl-α-D-arabinofuranosyl-(1→5)-2,3-di-*O*-acetyl-α-D-arabinofuranosyl-(1→5)-2,3-di-*O*-acetyl-α-D-arabinofuranoside (21)**

$^{1}$H NMR spectrum of compound **21** S62
$^{13}$C NMR spectrum of compound **21** S63

COSY $^{1}$H-$^{1}$H spectrum of compound **21** S64
HSQC $^{1}$H-$^{13}$C spectrum of compound **21** S65
HMBC $^{1}$H-$^{13}$C spectrum of compound **21** S66

# $^1$H NMR (600 MHz) spectrum of compound 2 in $CDCl_3$

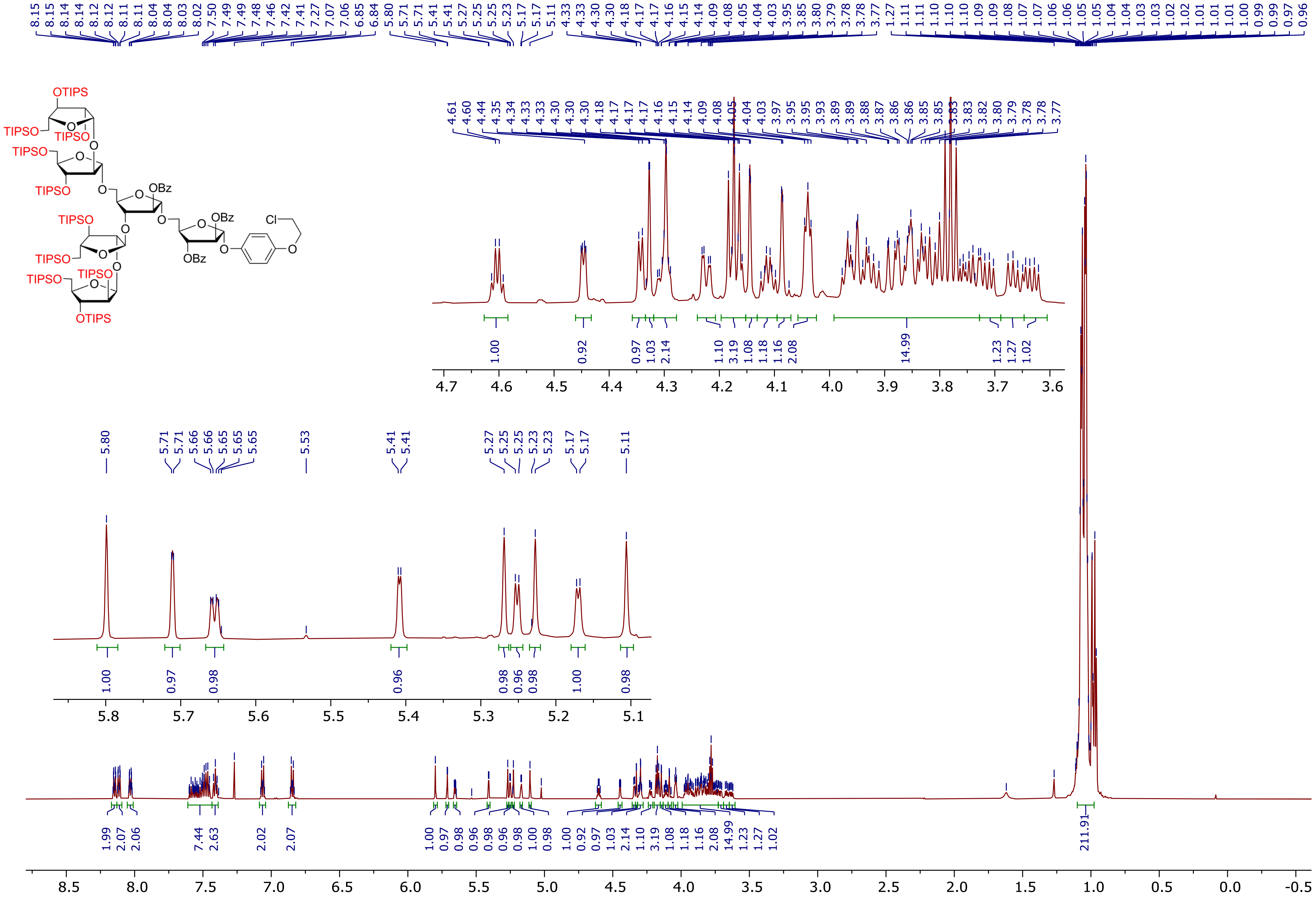

## $^{13}C$ NMR (151 MHz) spectrum of compound 2 in $CDCl_3$

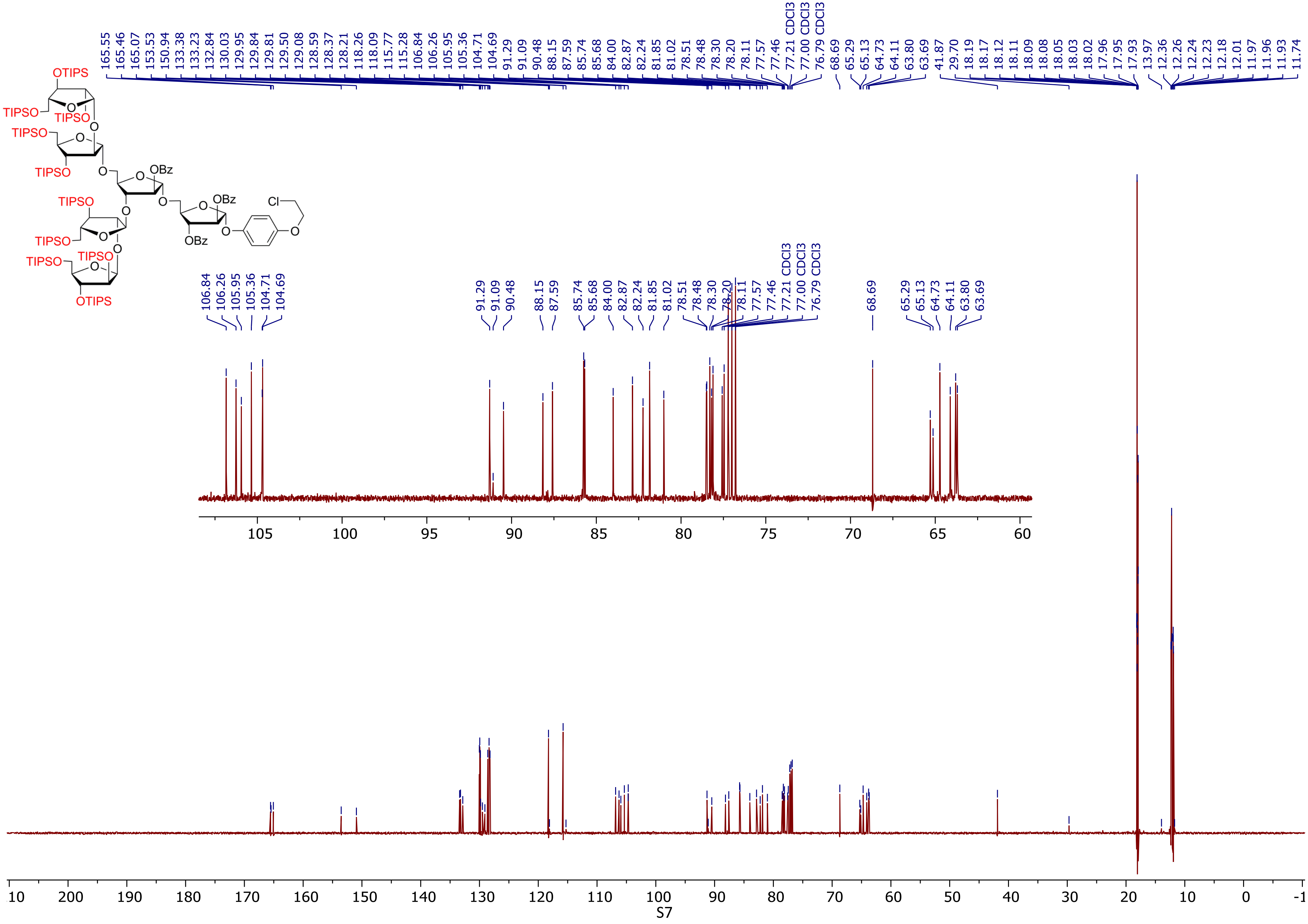

COSY (600 MHz) spectrum of compound 2 in $CDCl_3$

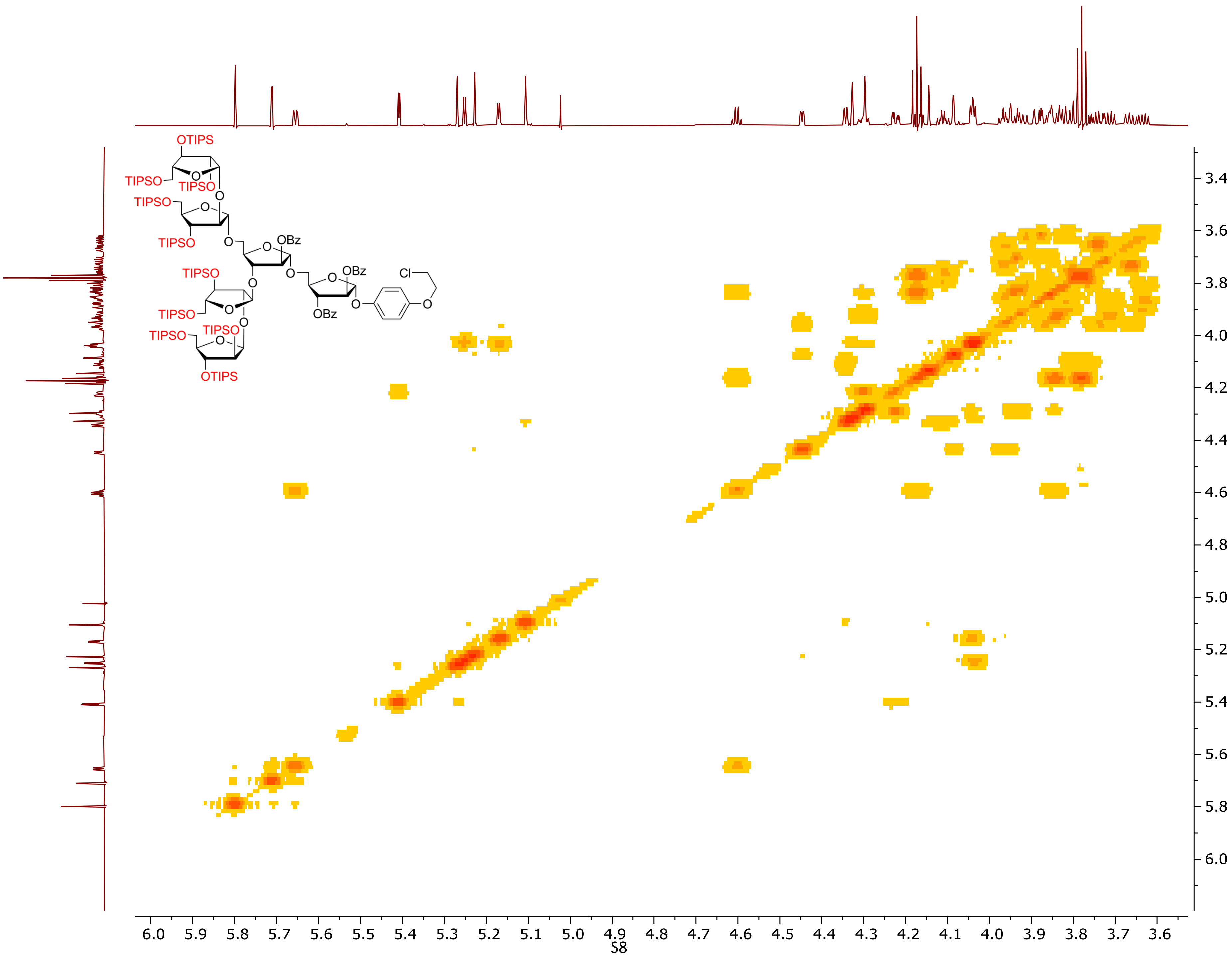

## HSQC (600 MHz) spectrum of compound 2 in $CDCl_3$

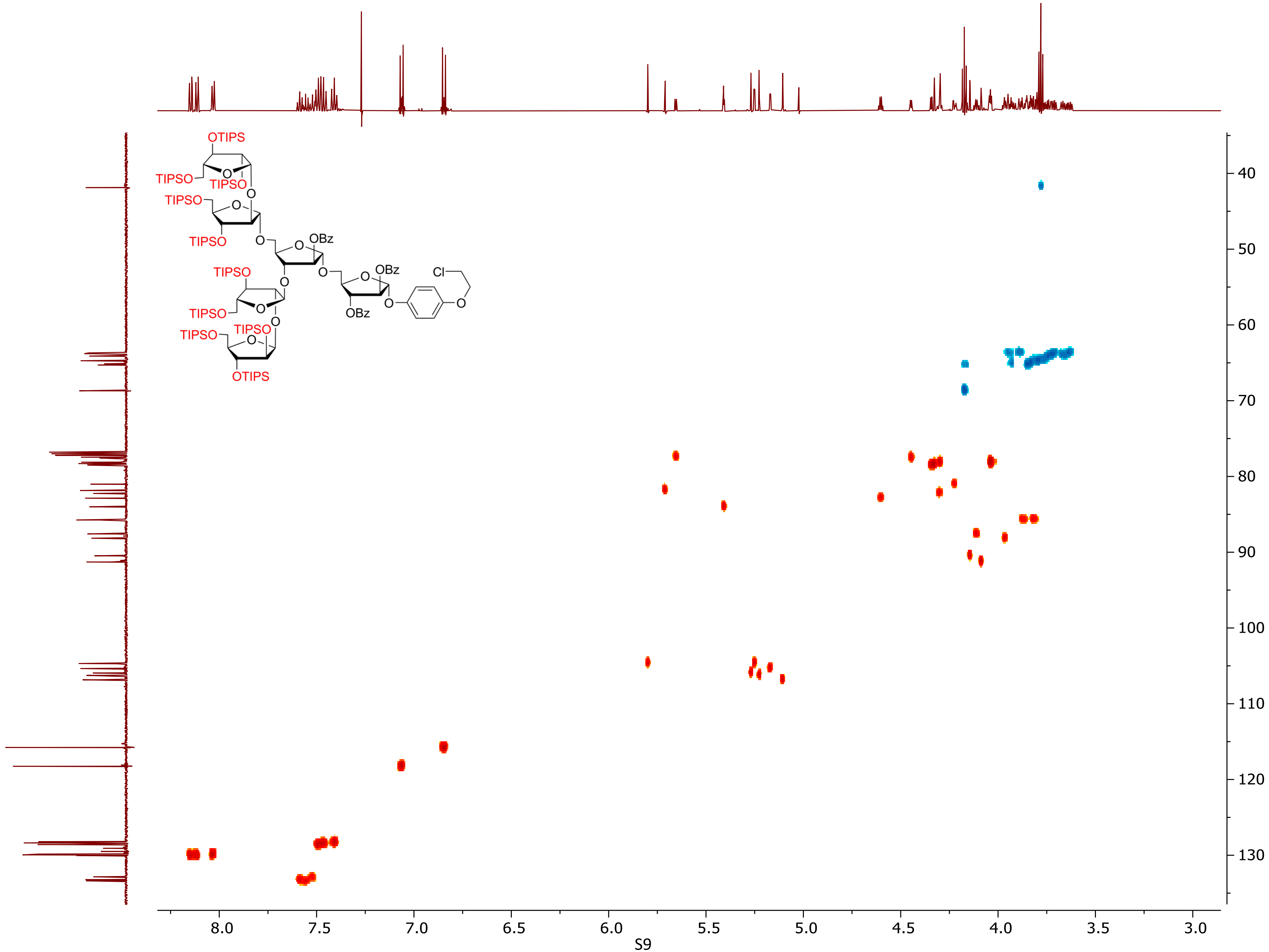

HMBC (600 MHz) spectrum of compound 2 in $CDCl_3$

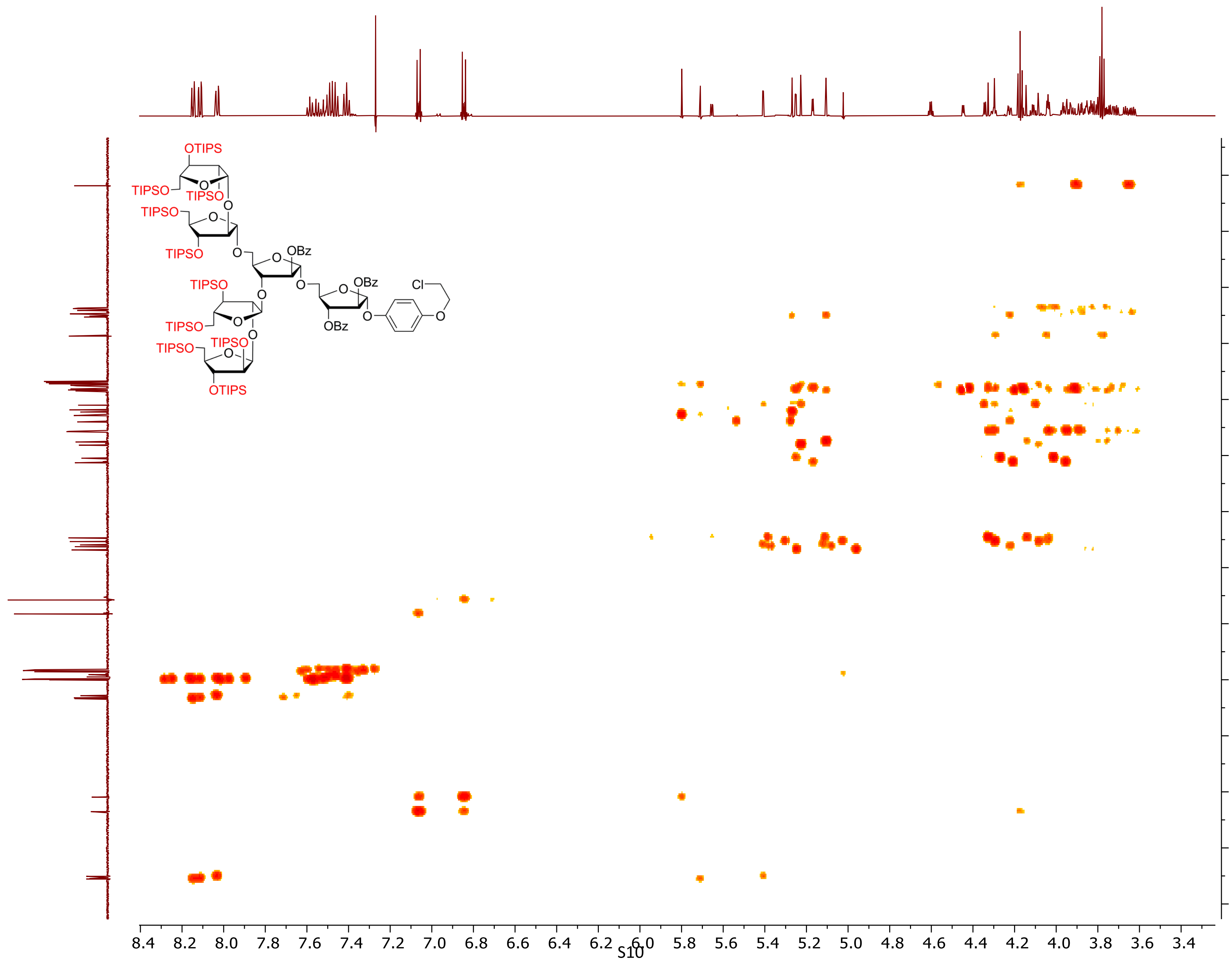

$^{1}H$–$^{29}Si$ HMBC (300 MHz) spectrum of compound 2 in $CDCl_3$

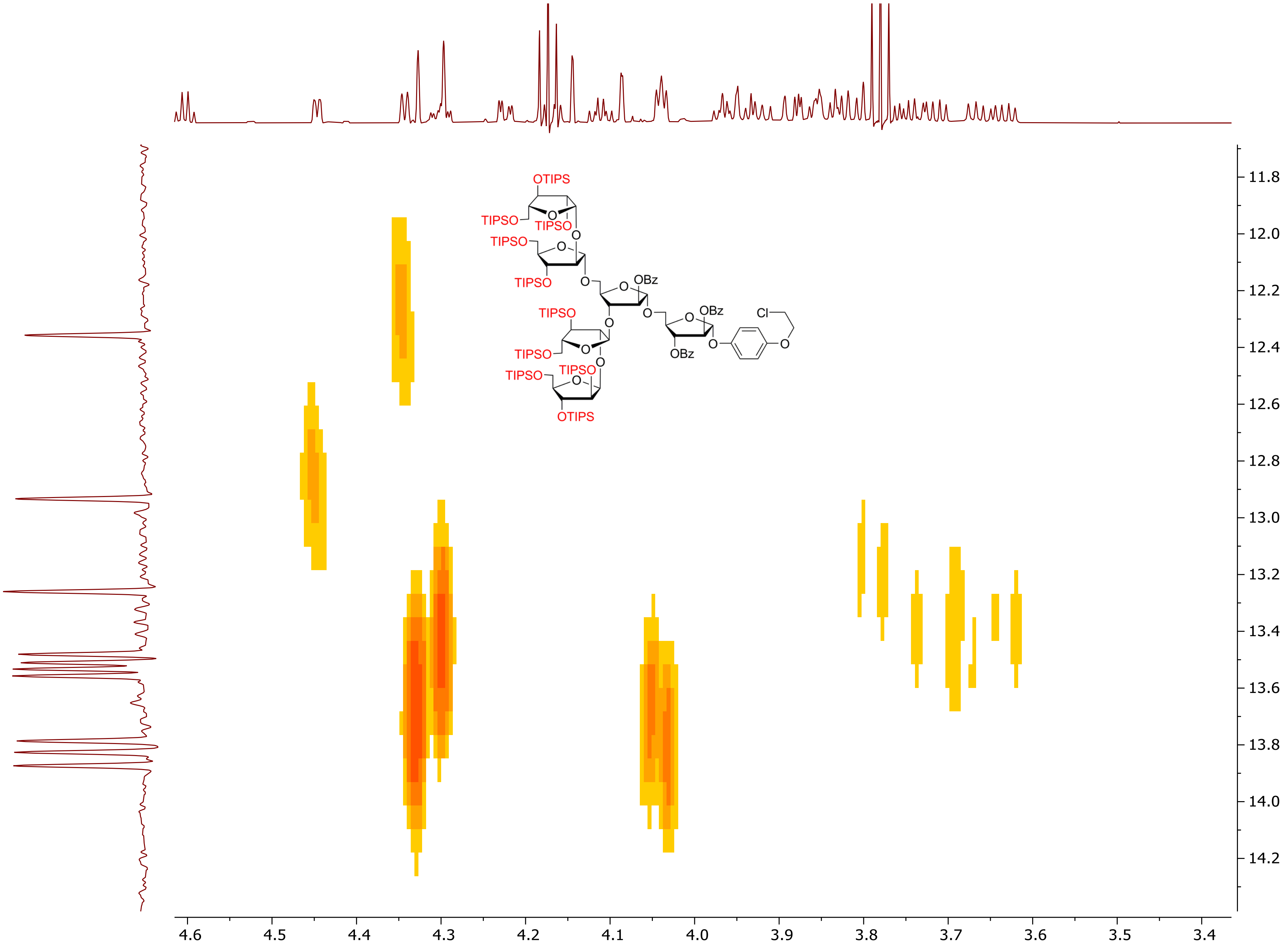

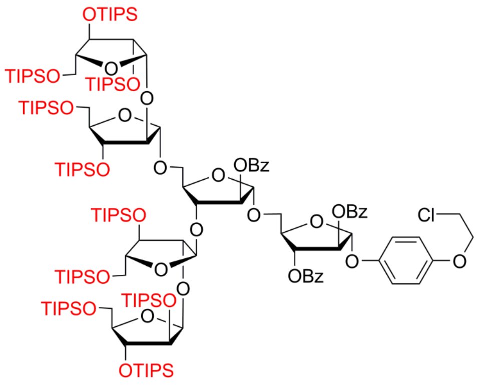


# $^{29}Si$ INEPT NMR (59.6 MHz) spectrum of compound 2 in $CDCl_3$

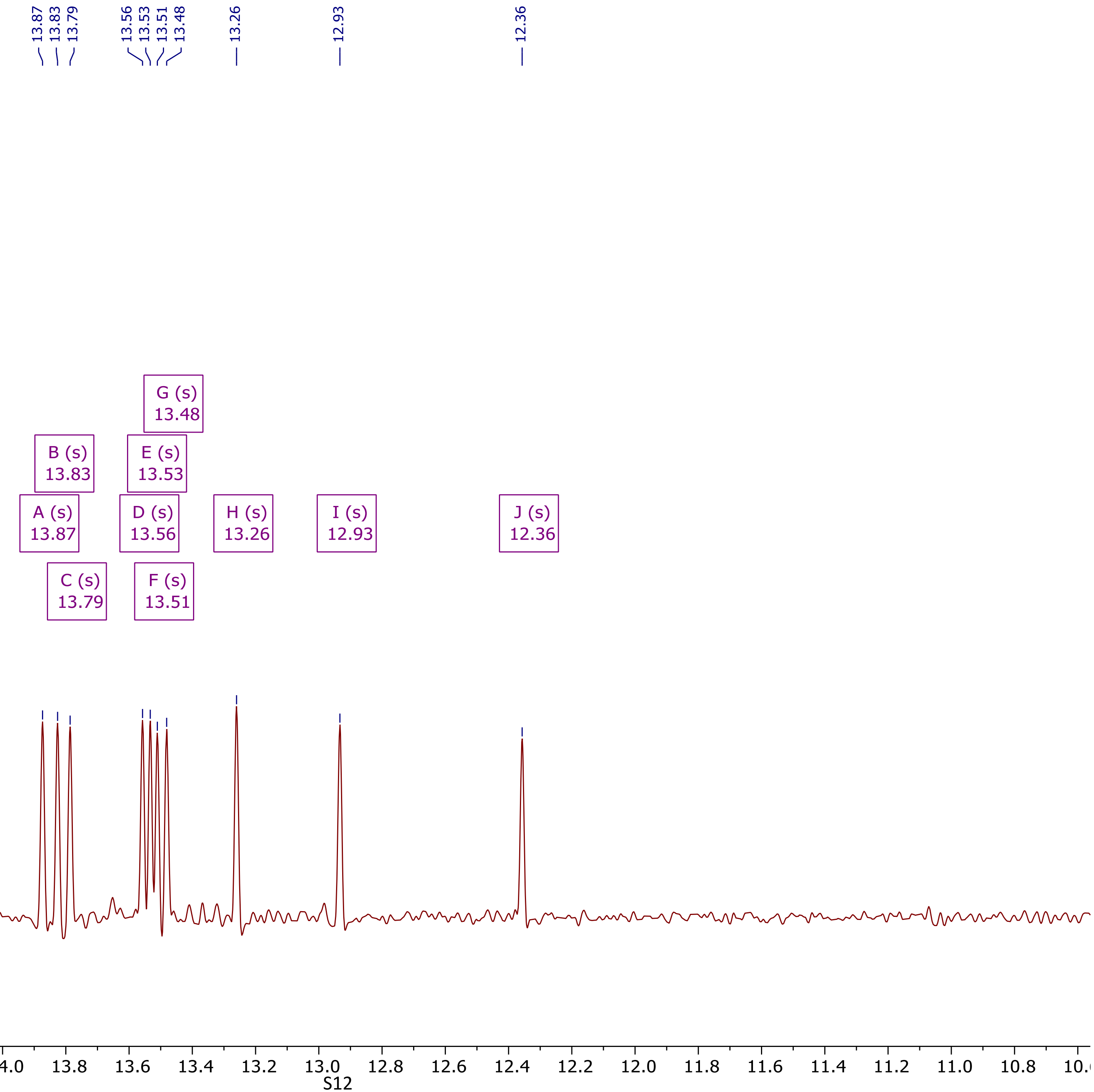

## $^{1}H$ NMR (300 MHz) spectrum of compound 4 in $CDCl_3$

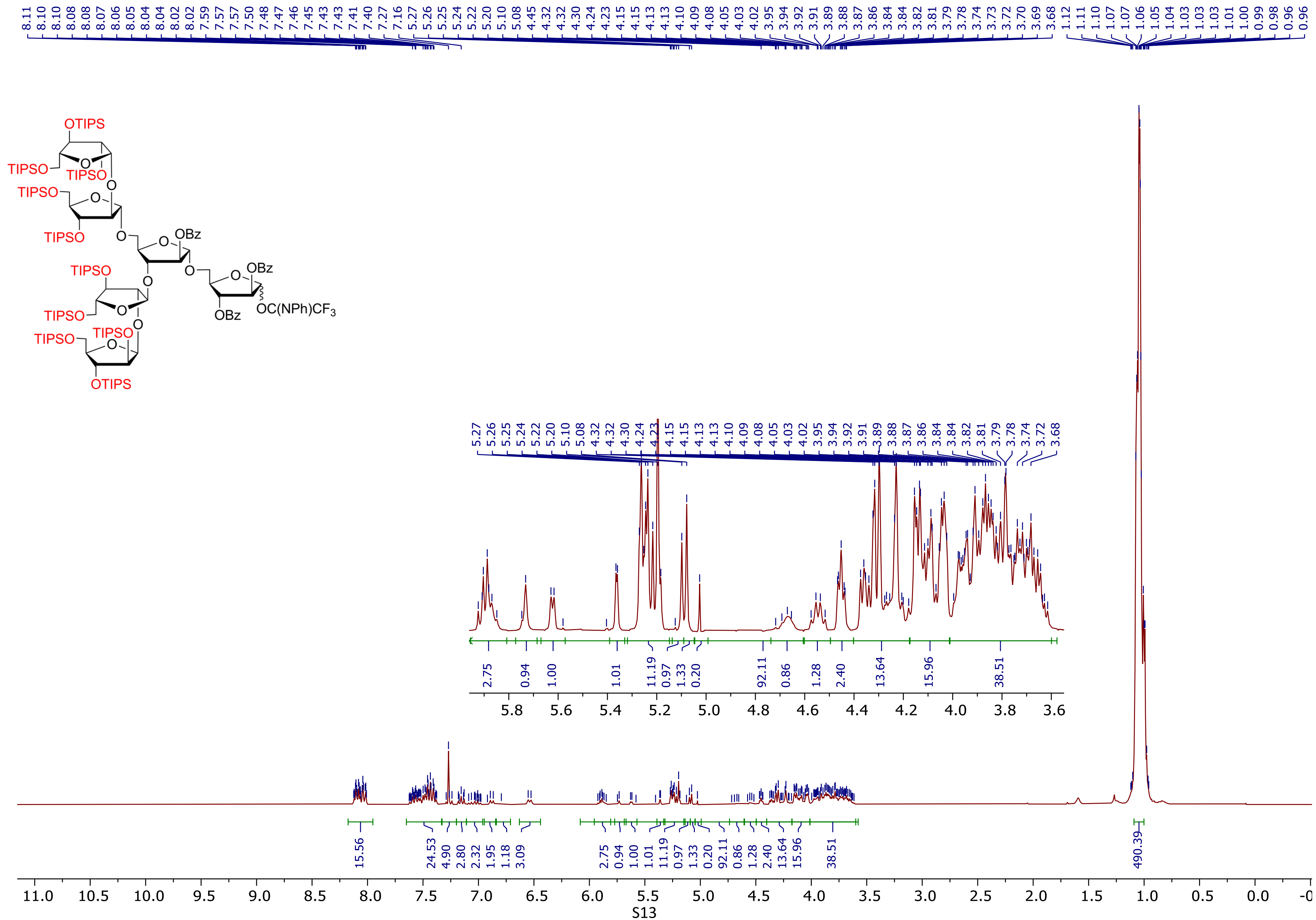

## $^{13}C$ NMR (76 MHz) spectrum of compound 4 in $CDCl_3$

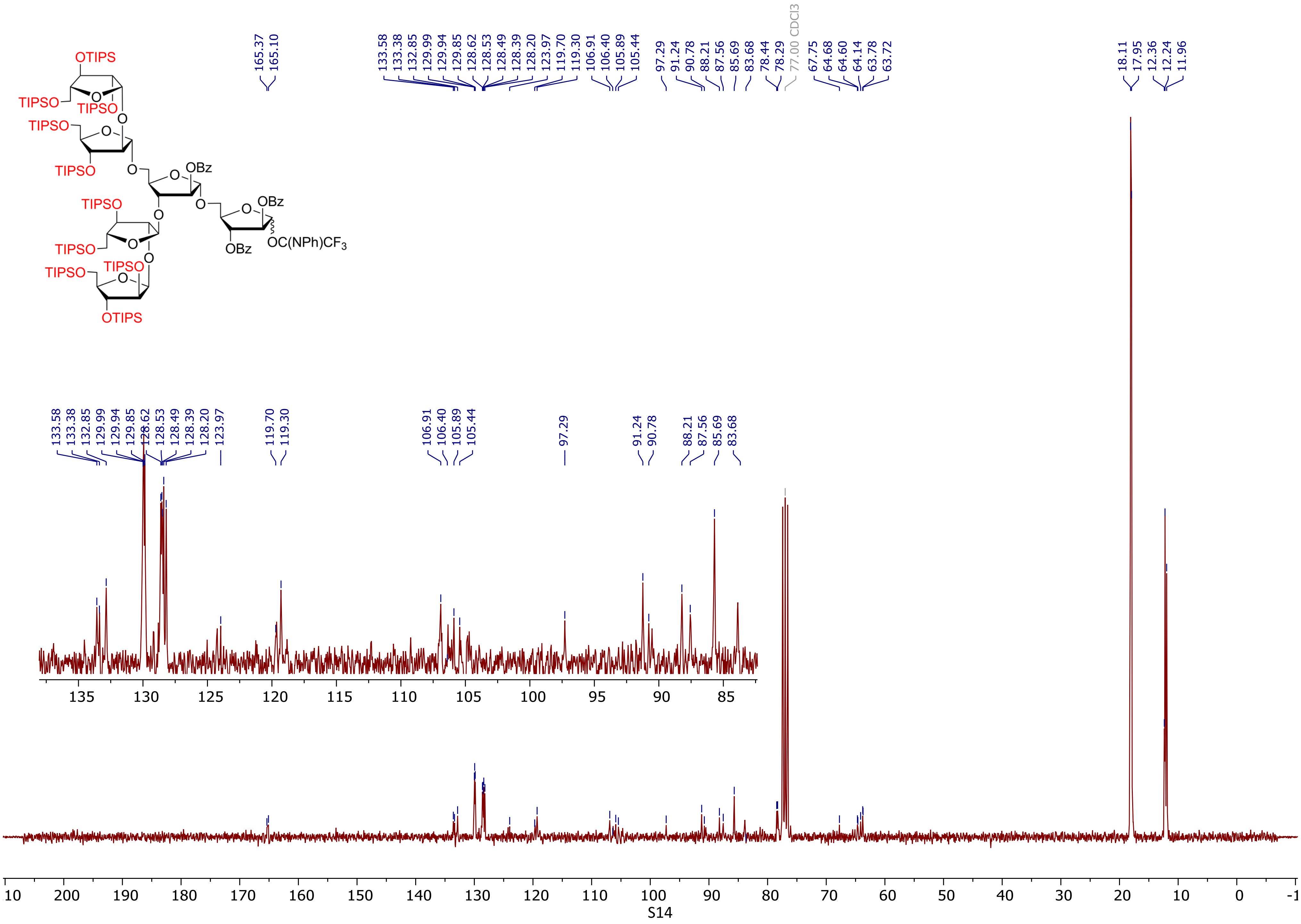

## COSY (300 MHz) spectrum of compound 4 in $CDCl_3$

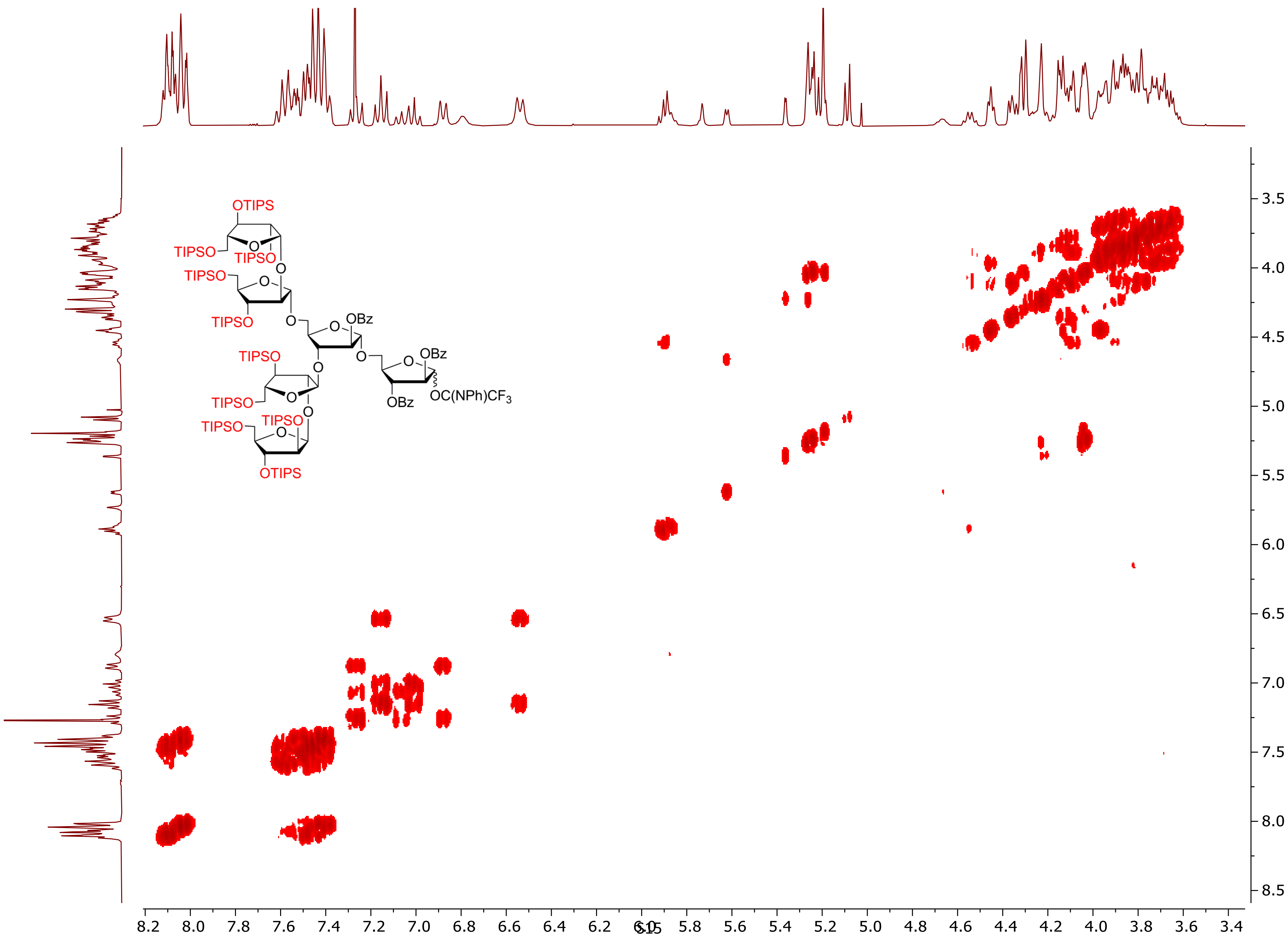

## HSQC (300 MHz) spectrum of compound 4 in $CDCl_3$

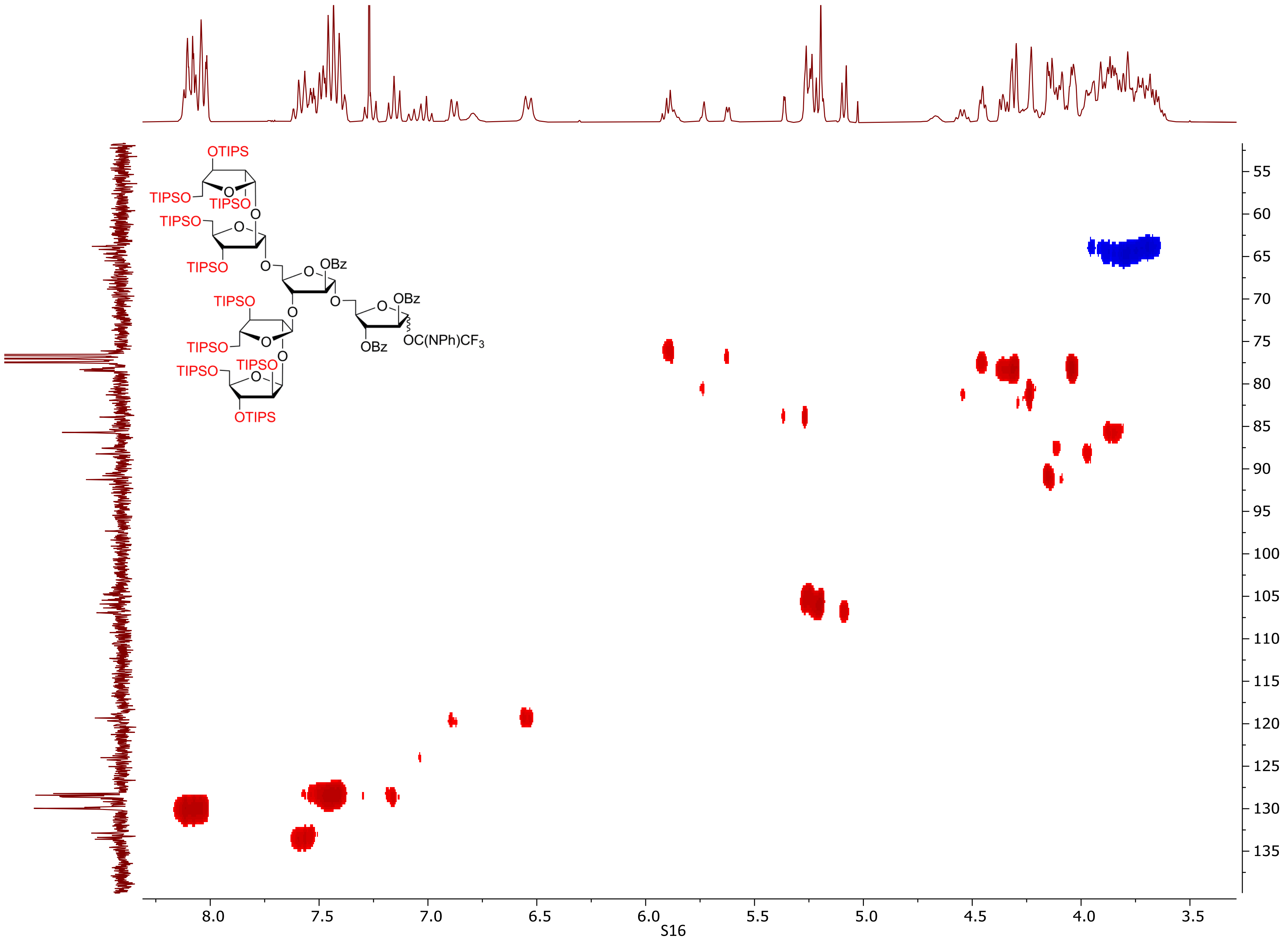

# $^{29}$Si INEPT NMR (59.6 MHz) spectrum of compound 4 in $CDCl_3$

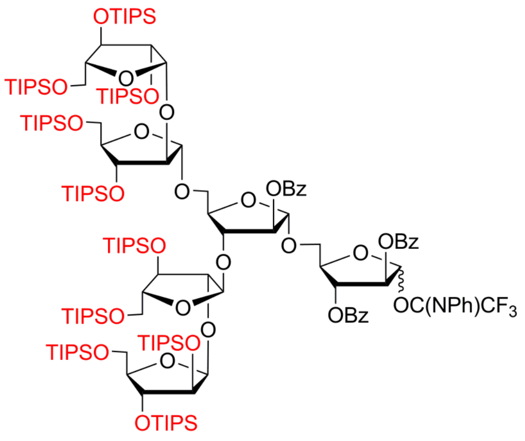


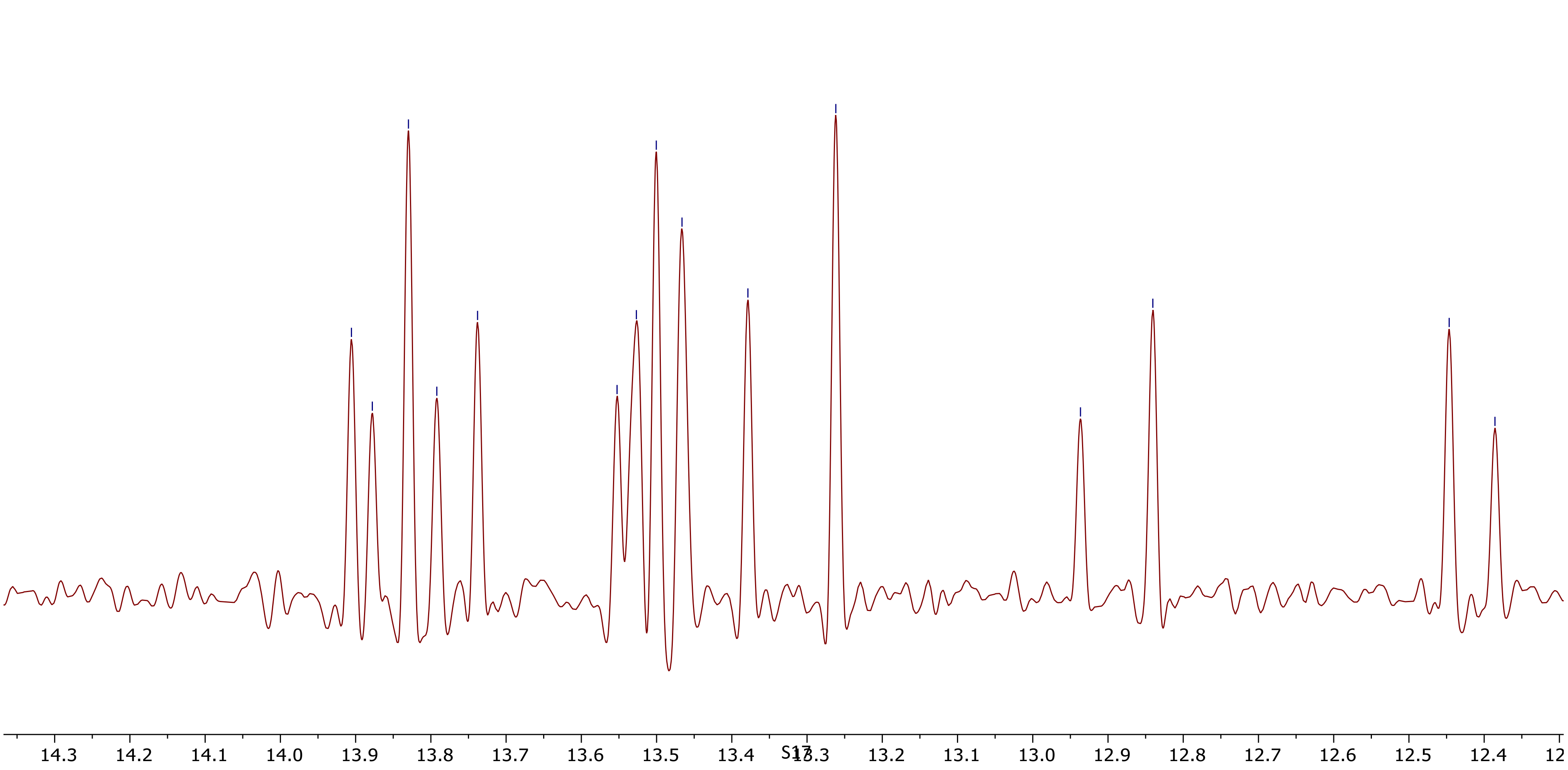

## $^{19}F$ NMR (298 MHz) spectrum of compound 4 in $CDCl_3$

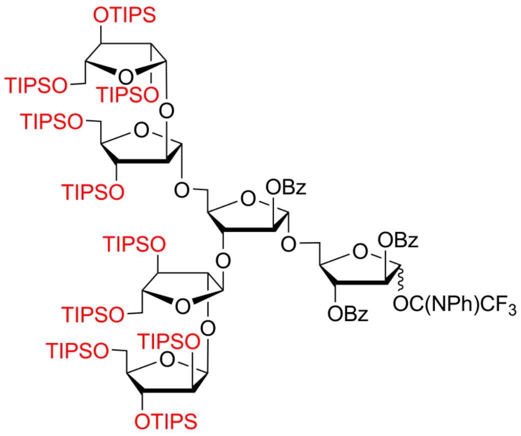


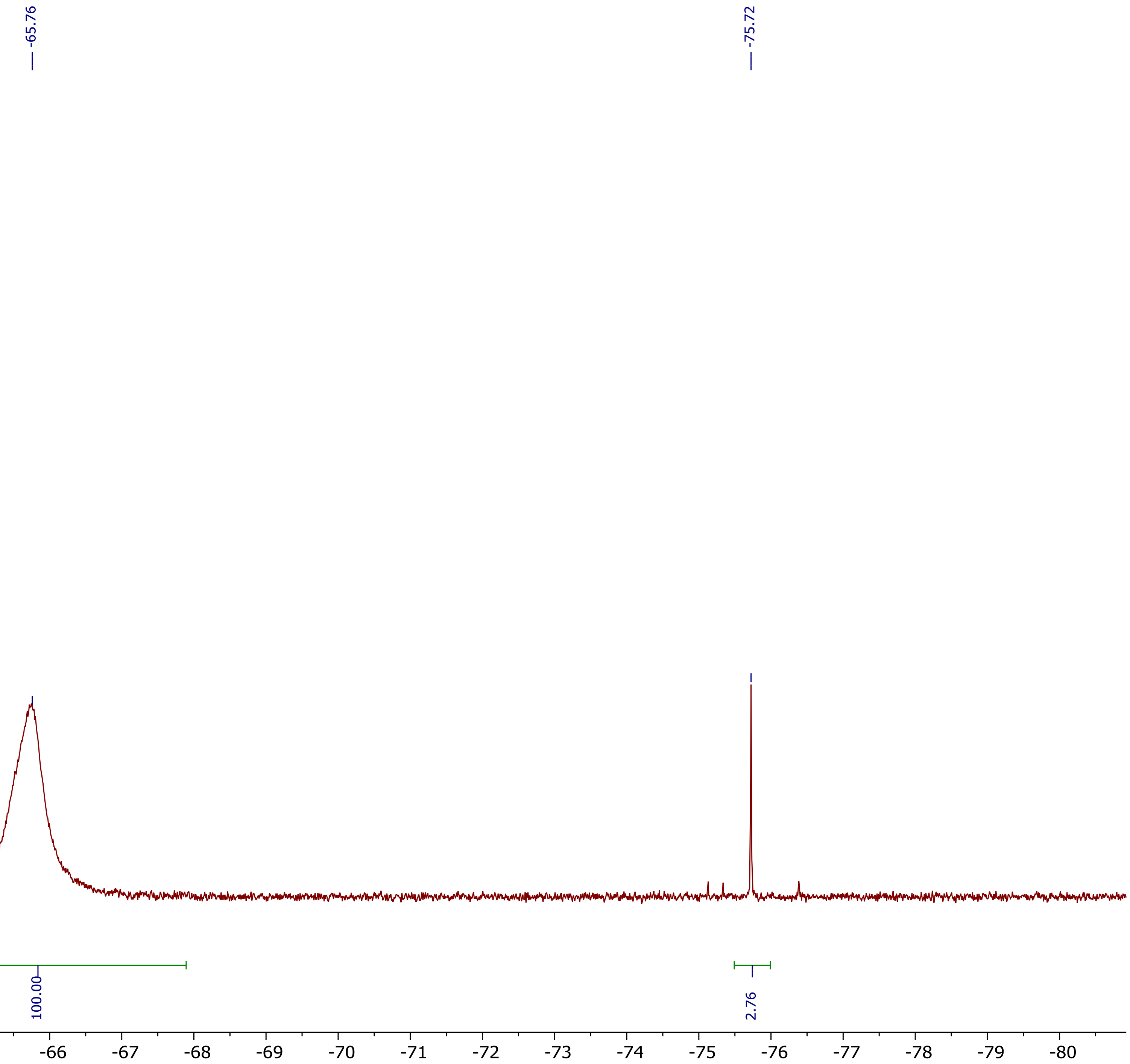

## $^1$H NMR (600 MHz) spectrum of compound 5 in $CDCl_3$

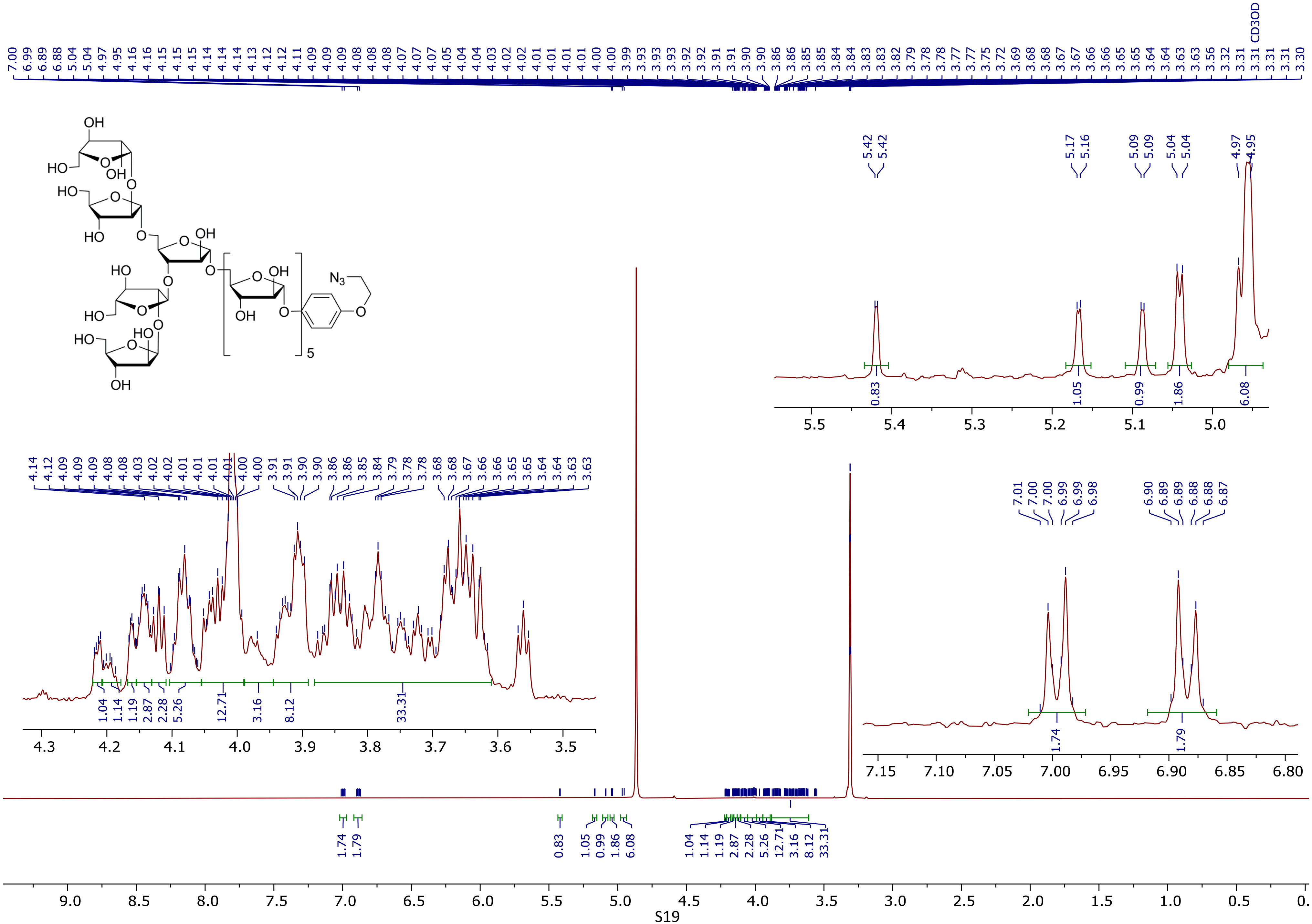

## $^{13}C$ NMR (151 MHz) spectrum of compound 5 in $CDCl_3$

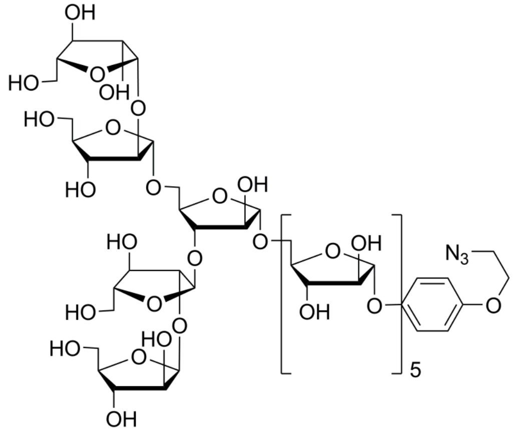

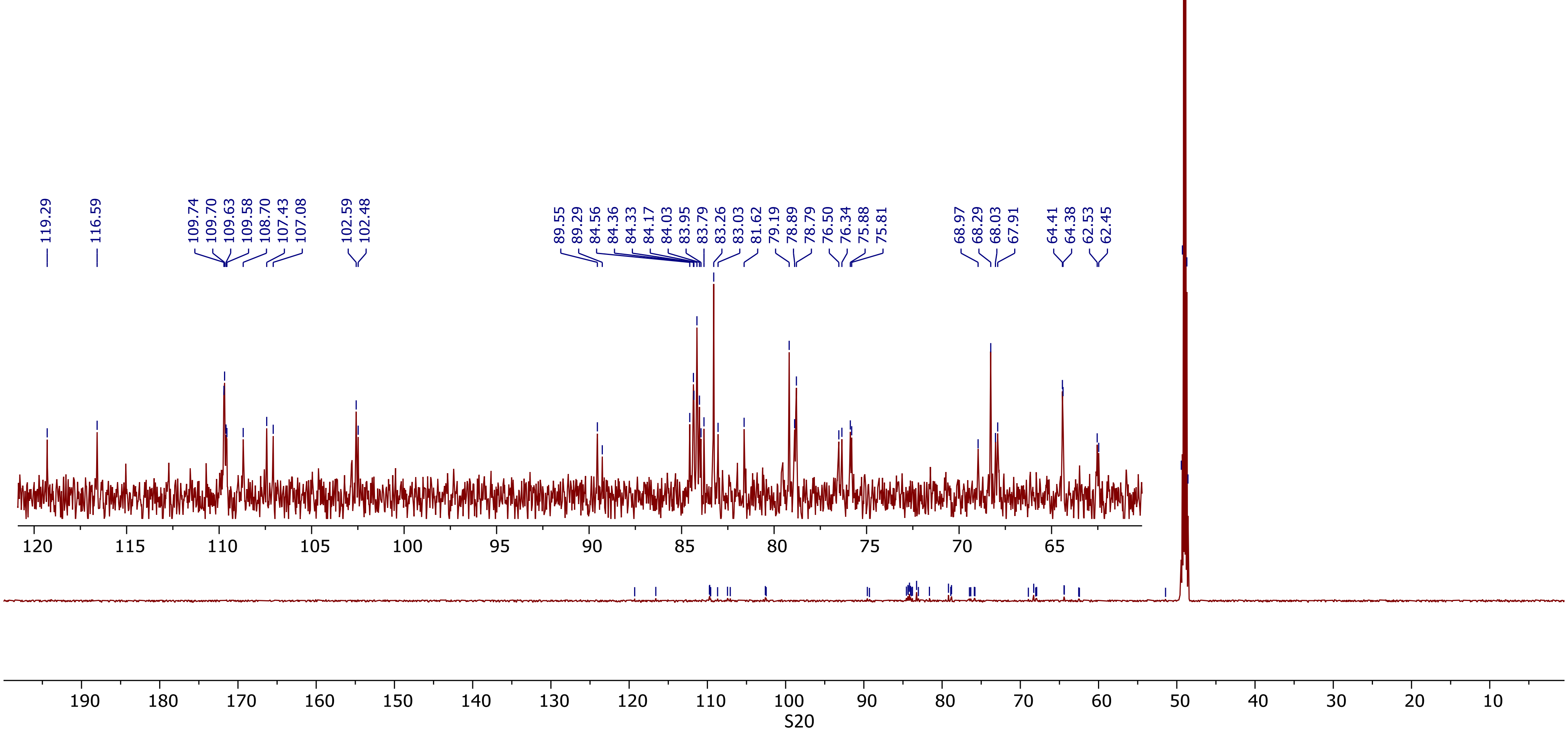

## COSY (600 MHz) spectrum of compound 5 in $CDCl_3$

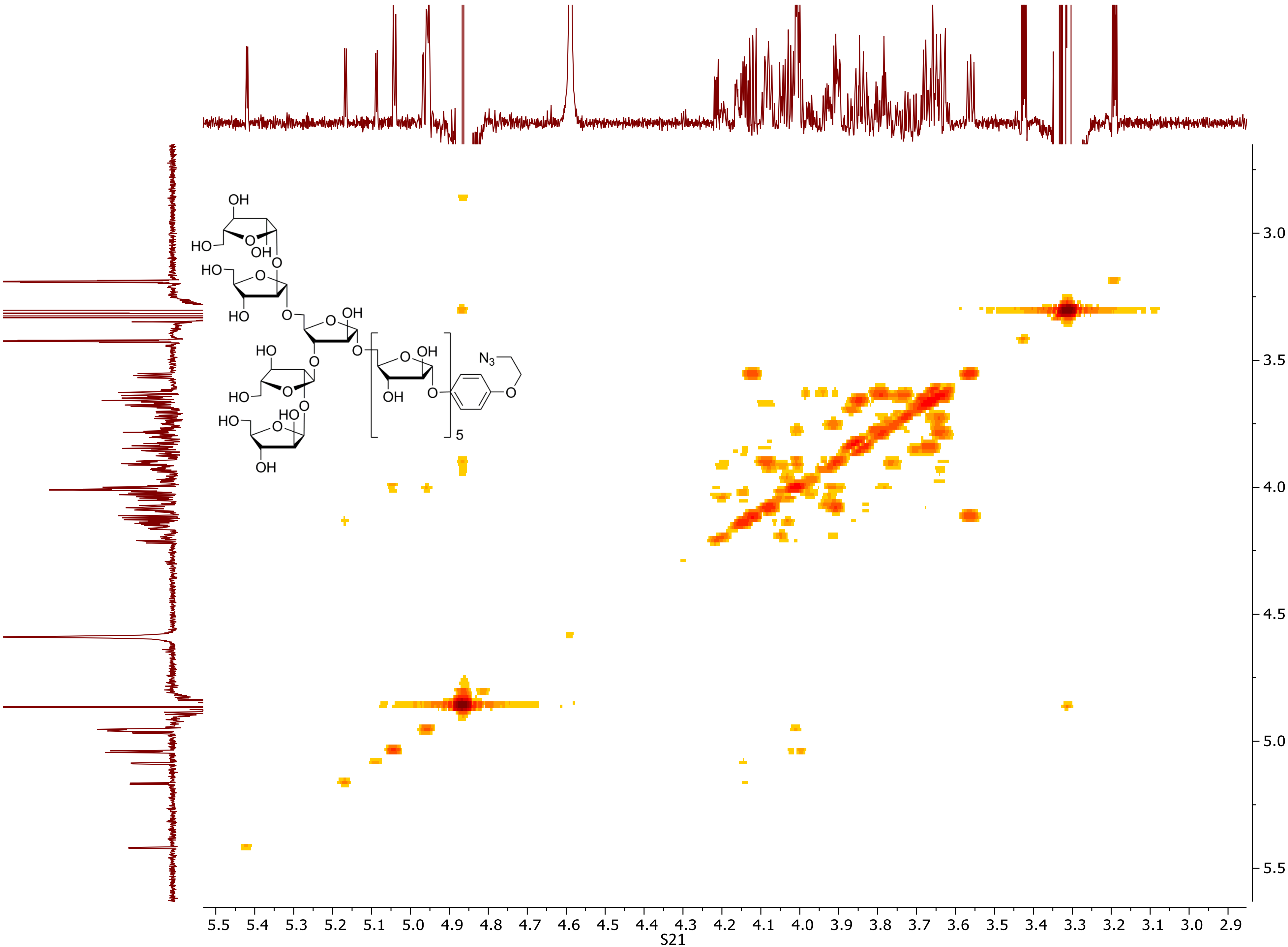

# HSQC (600 MHz) spectrum of compound 5 in $CDCl_3$

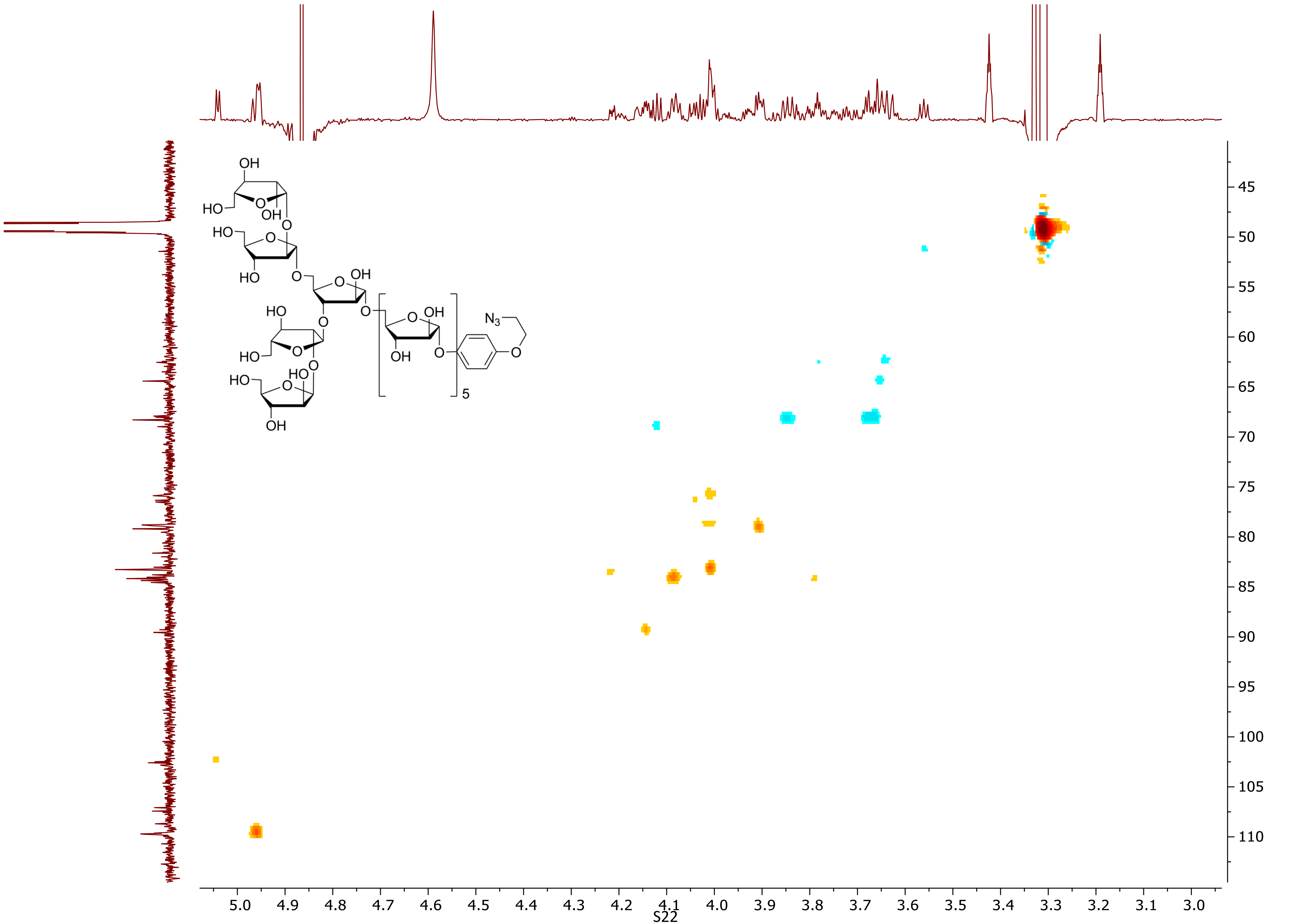

**HMBC (600 MHz) spectrum of compound 5 in $CDCl_3$**

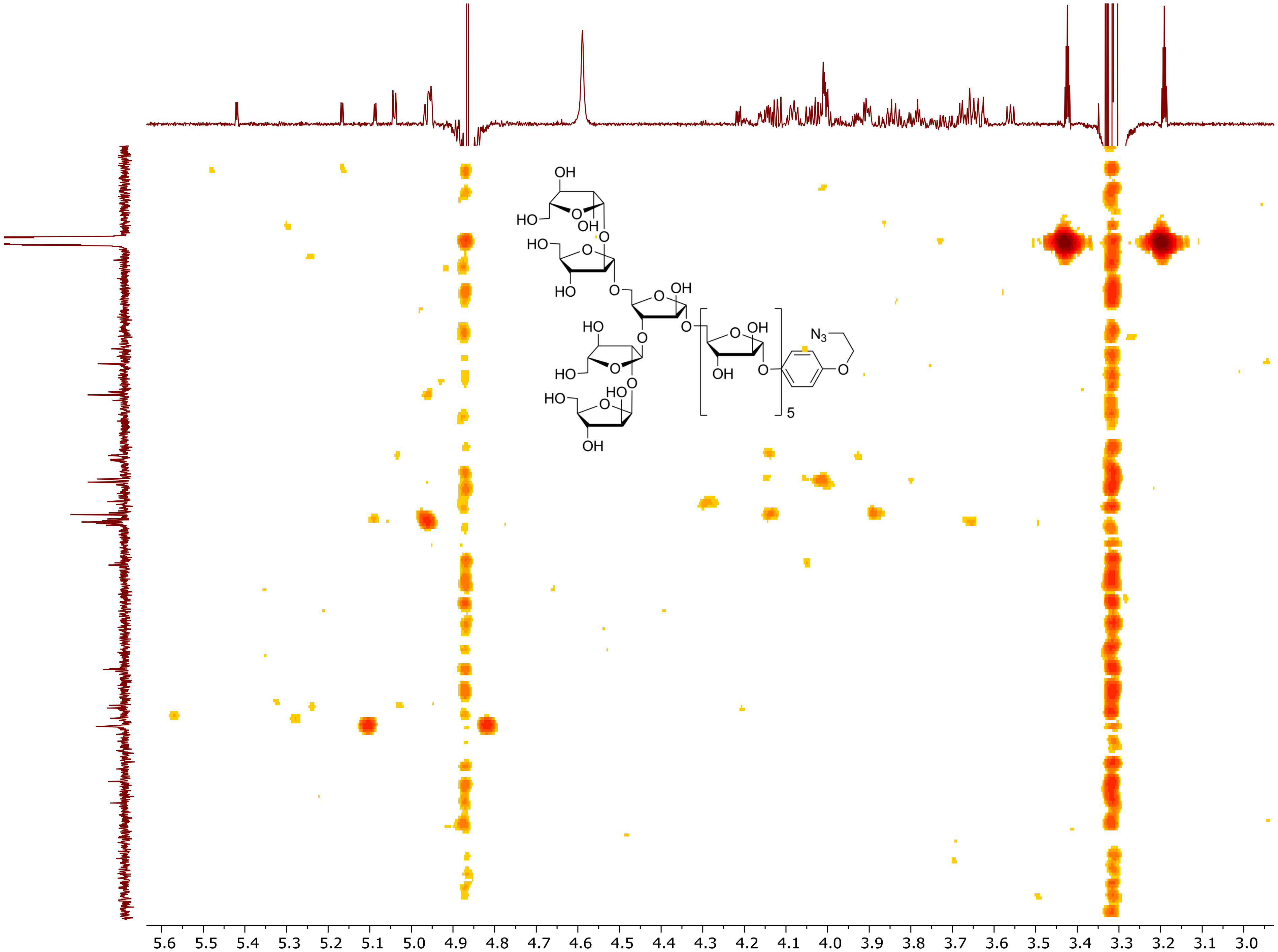

## $^1$H NMR (600 MHz) spectrum of compound 10 in $CDCl_3$

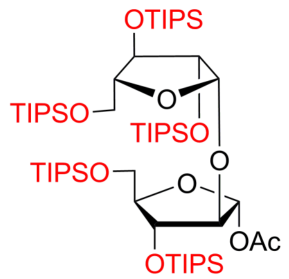

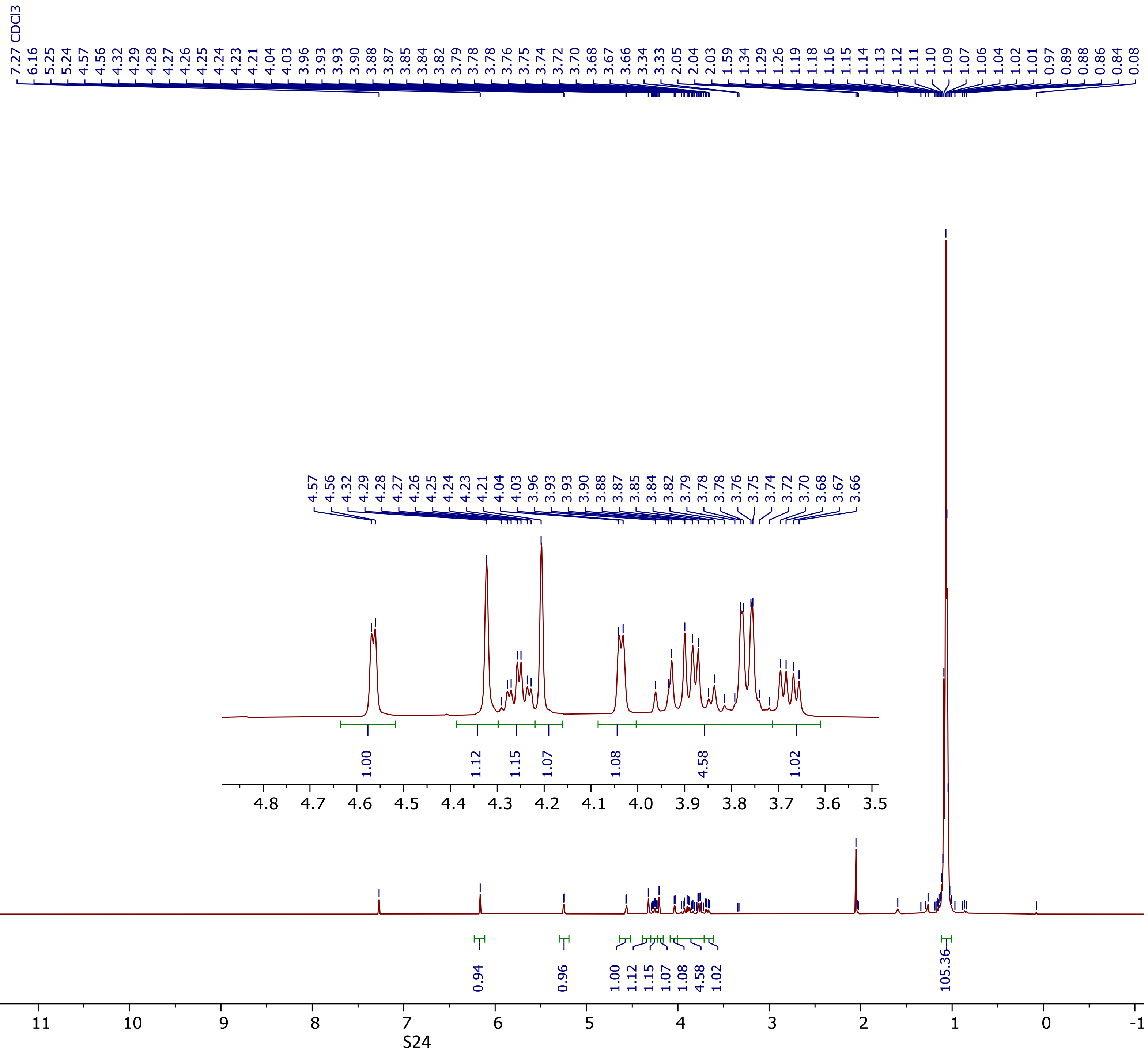

## $^{13}$C NMR (151 MHz) spectrum of compound 10 in $CDCl_3$

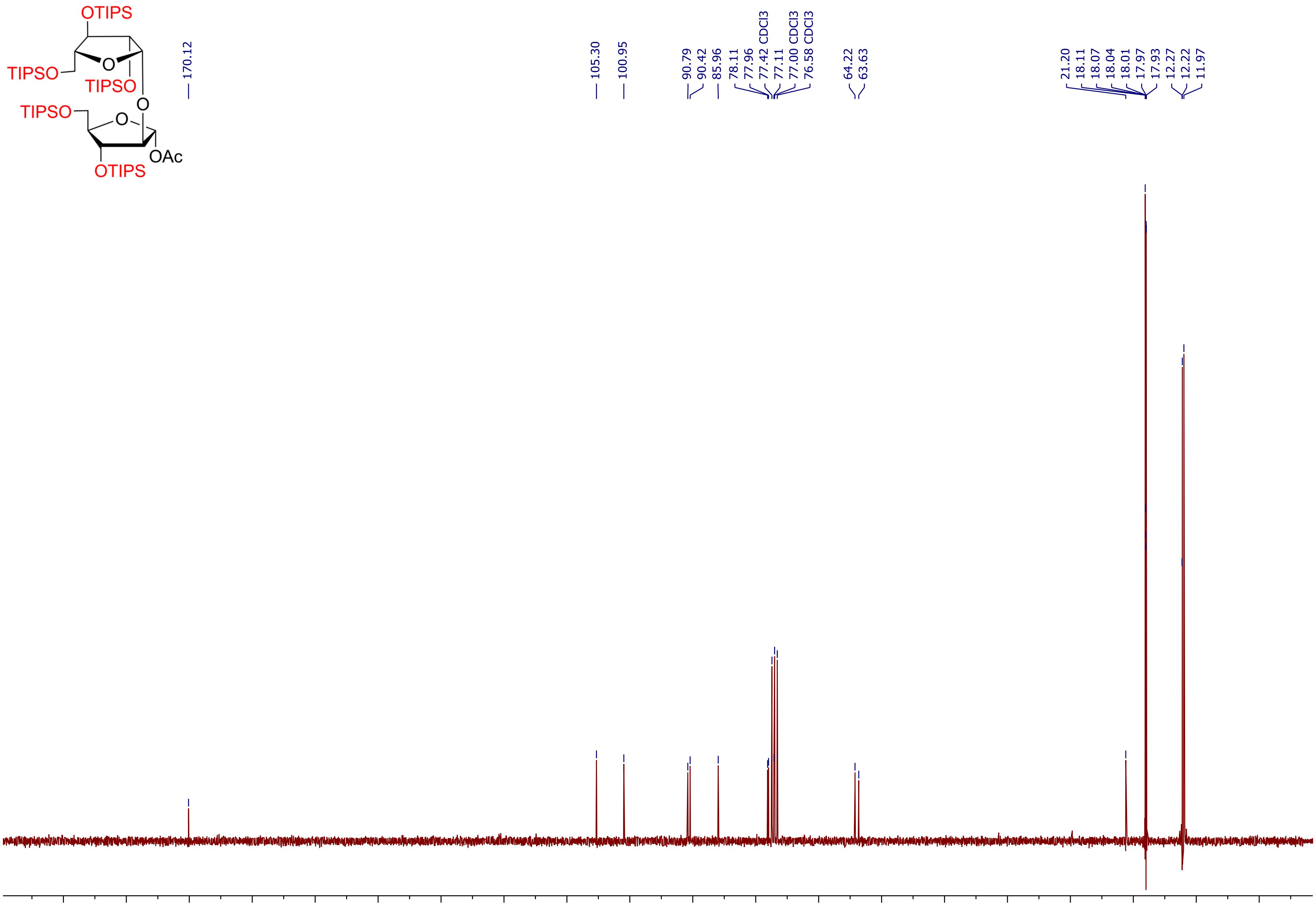

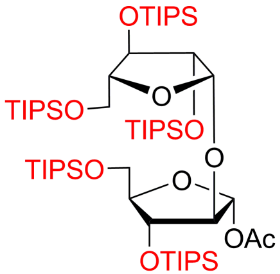


# COSY (600 MHz) spectrum of compound 10 in $CDCl_3$

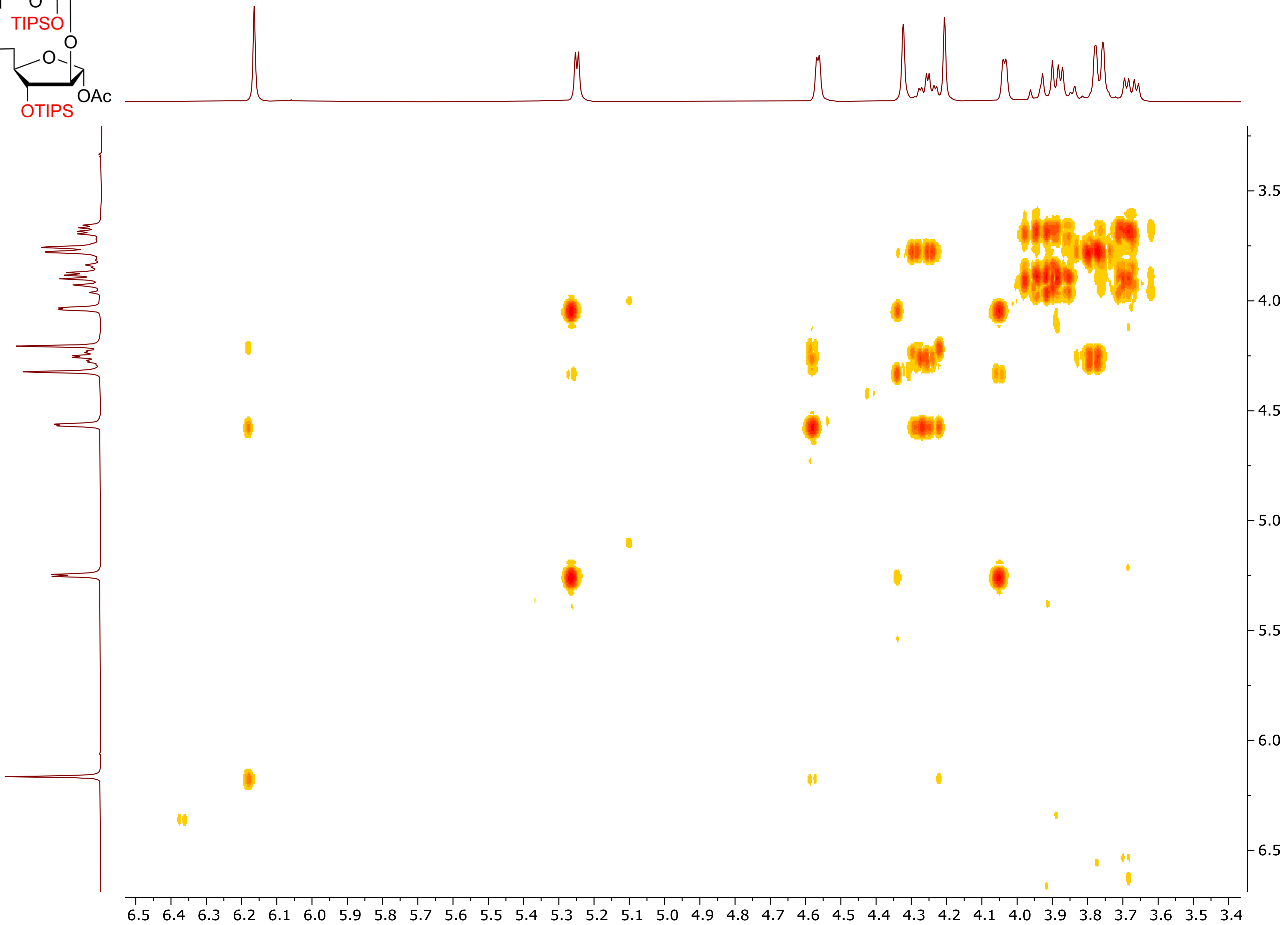

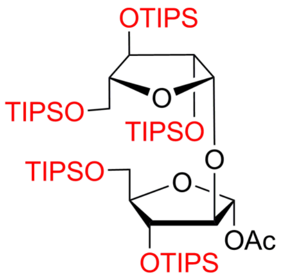


## HSQC (600 MHz) spectrum of compound 10 in $CDCl_3$

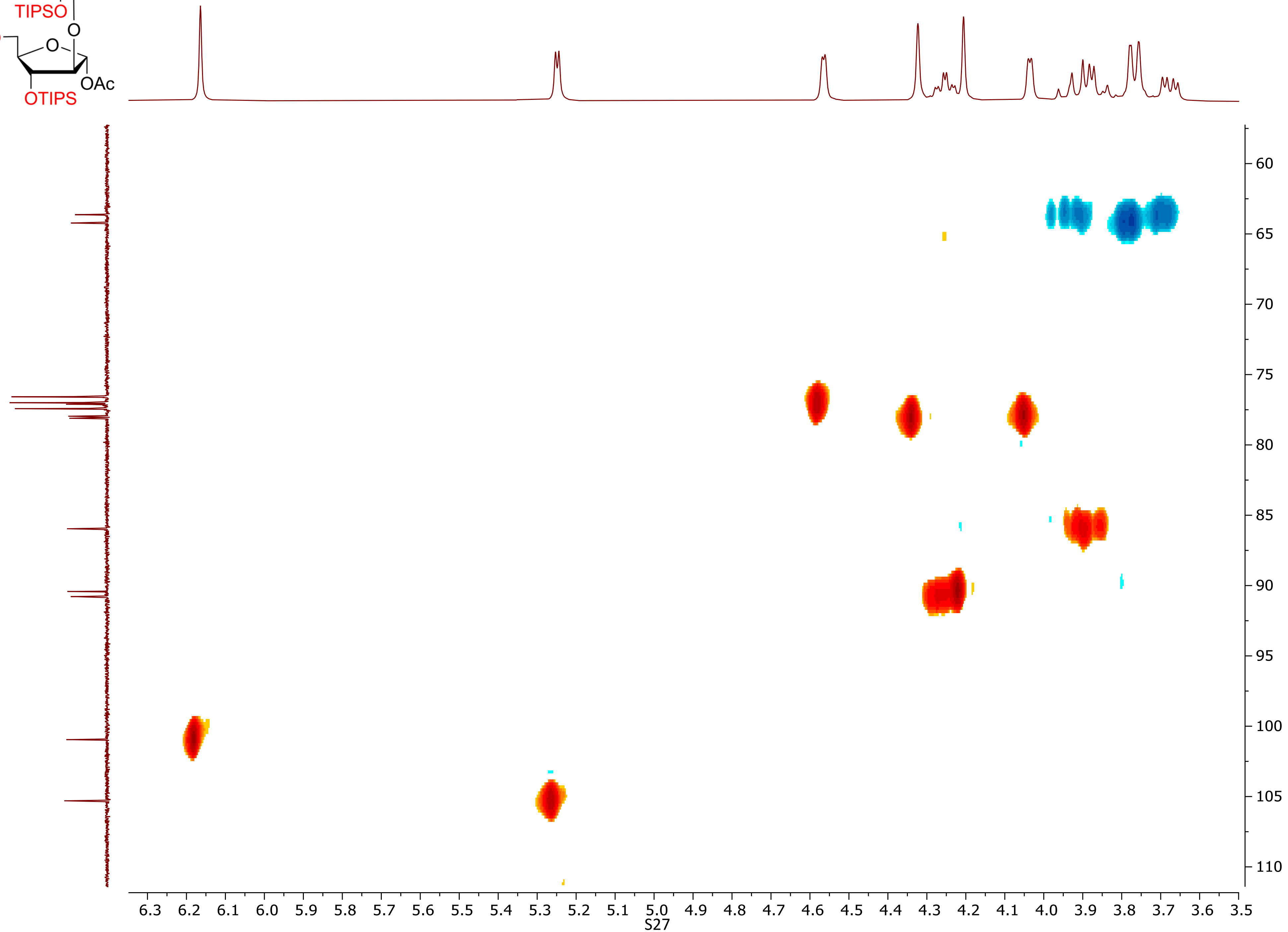

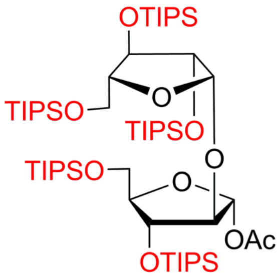


## HMBC (600 MHz) spectrum of compound 10 in $CDCl_3$

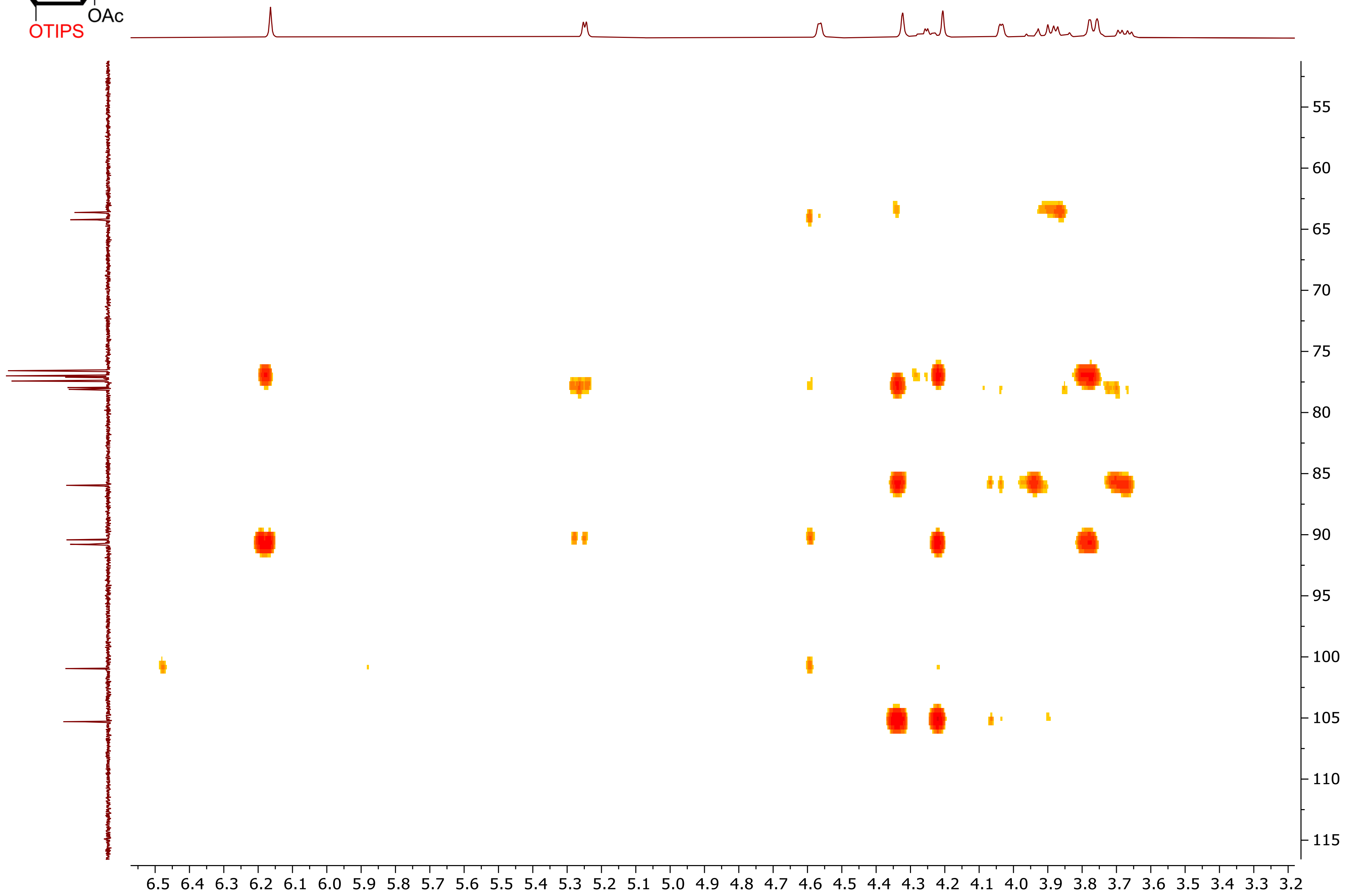

$^{1}H–^{29}Si$ HMBC (300 MHz) spectrum of compound 10 in $CDCl_3$

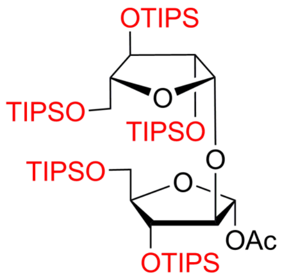


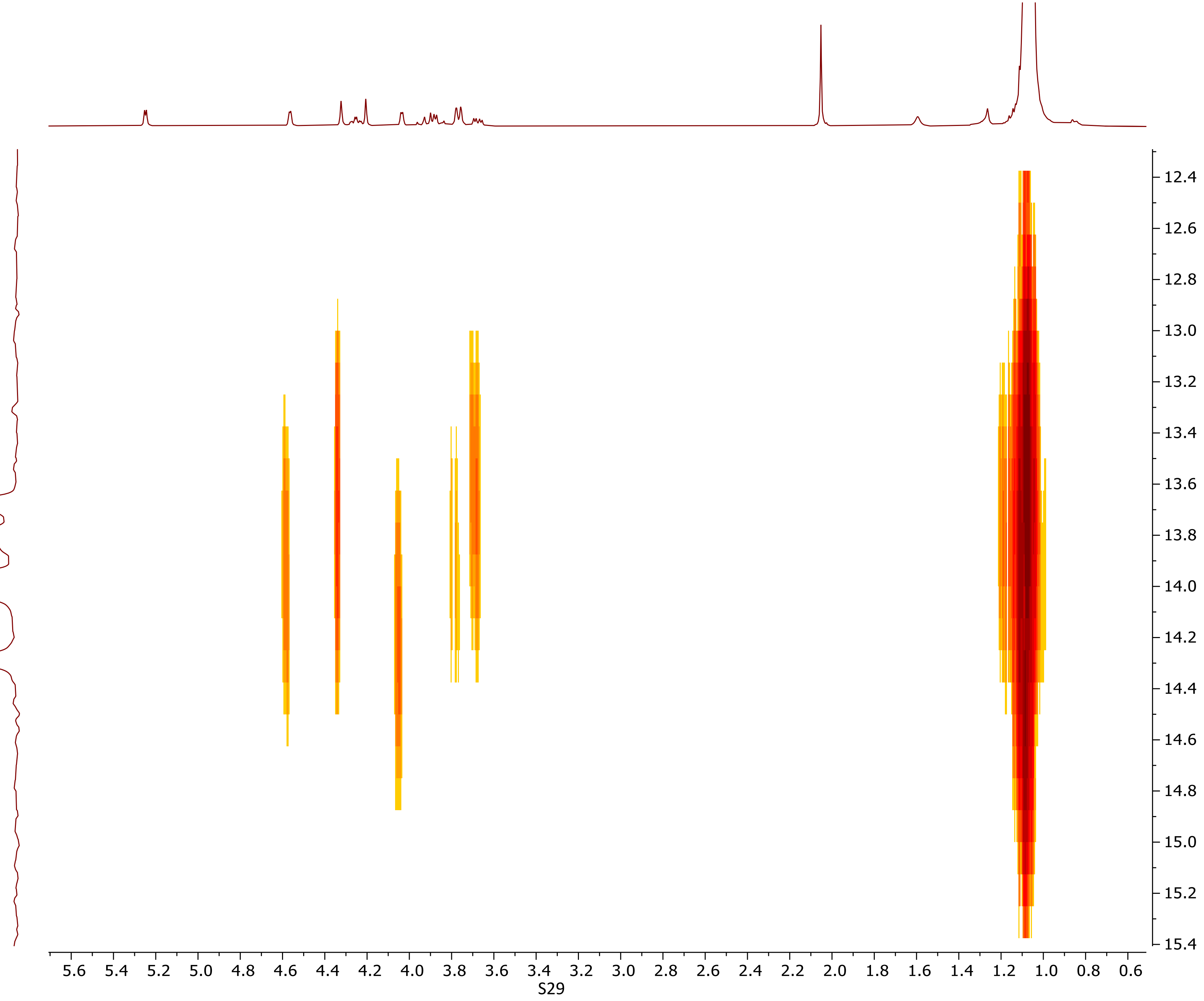

## $^{29}$Si INEPT NMR (59.6 MHz) spectrum of compound 10 in $CDCl_3$

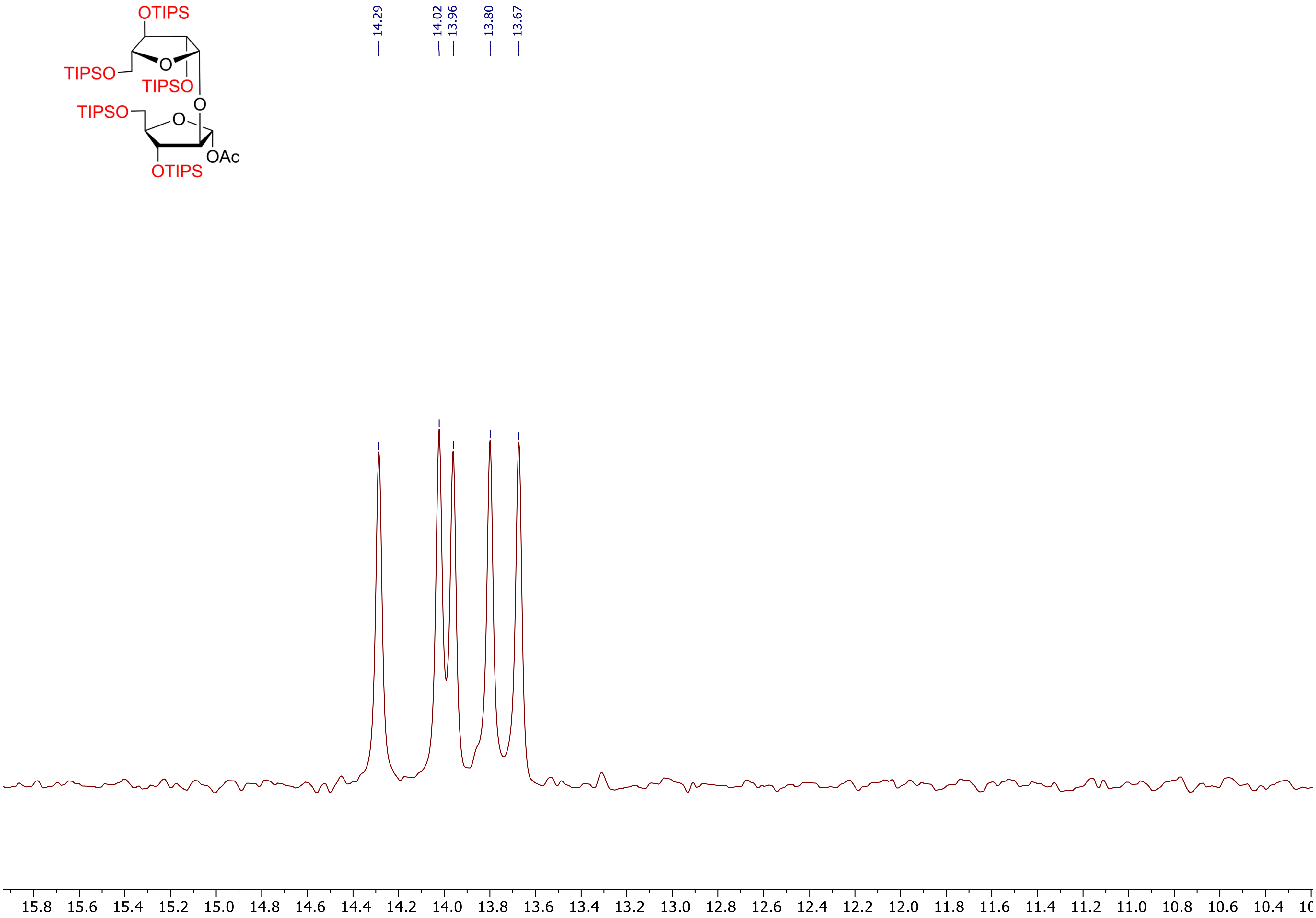

## $^{1}$H NMR (600 MHz) spectrum of compound 13 in $CDCl_3$

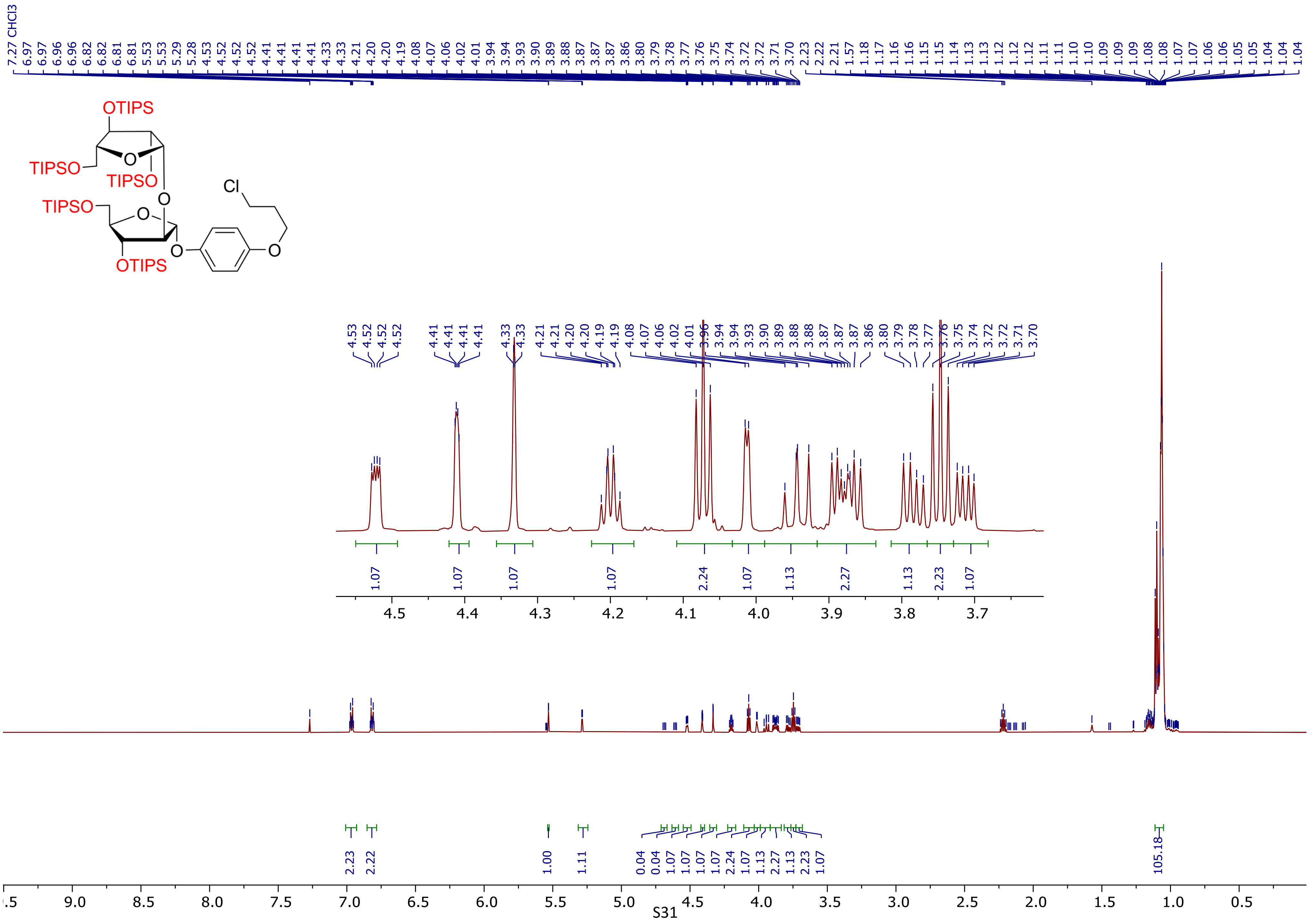

## $^{13}C$ NMR (151 MHz) spectrum of compound 13 in $CDCl_3$

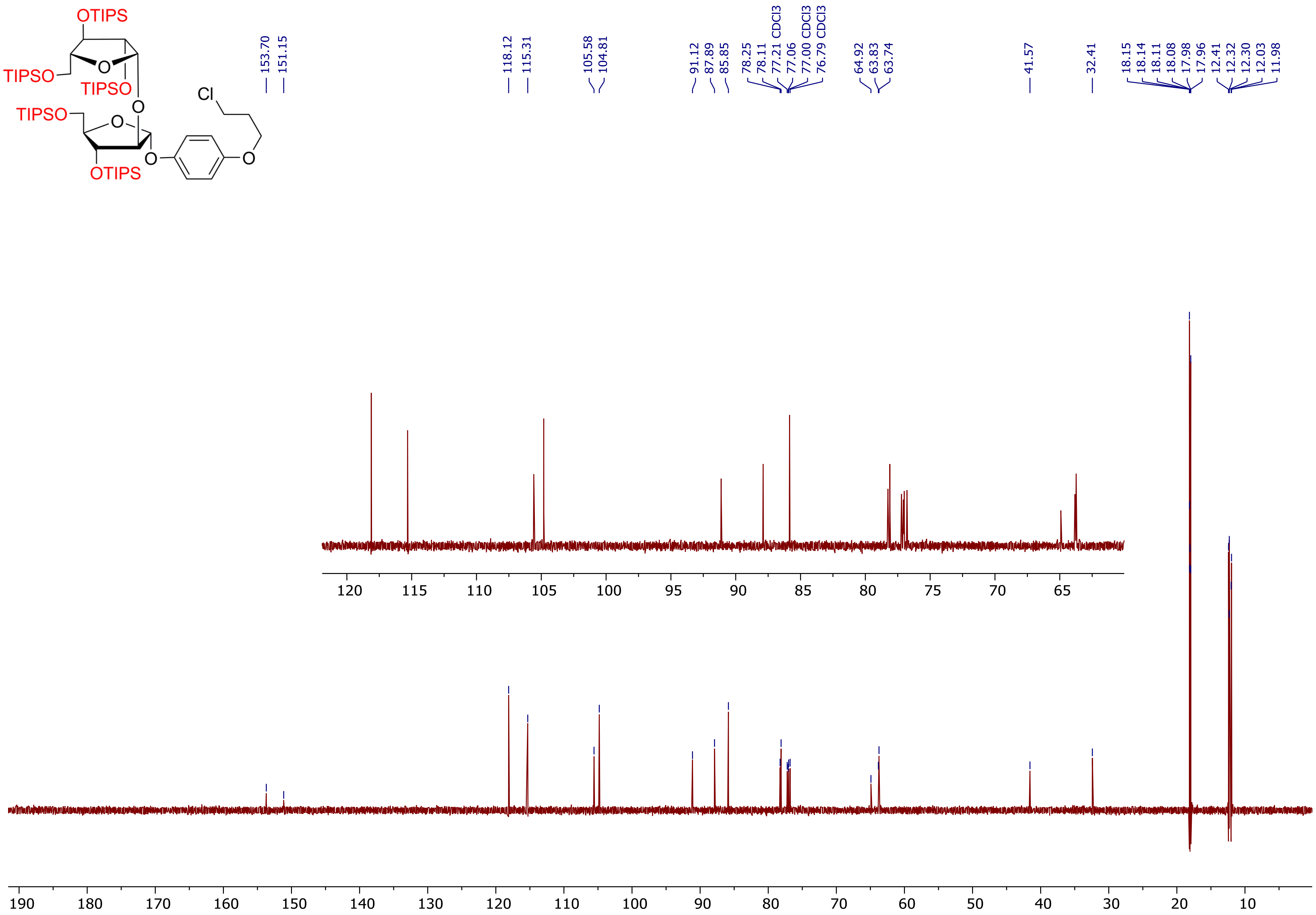

# COSY (600 MHz) spectrum of compound 13 in $CDCl_3$

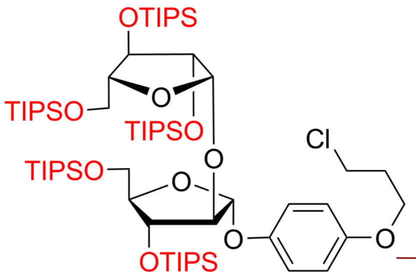


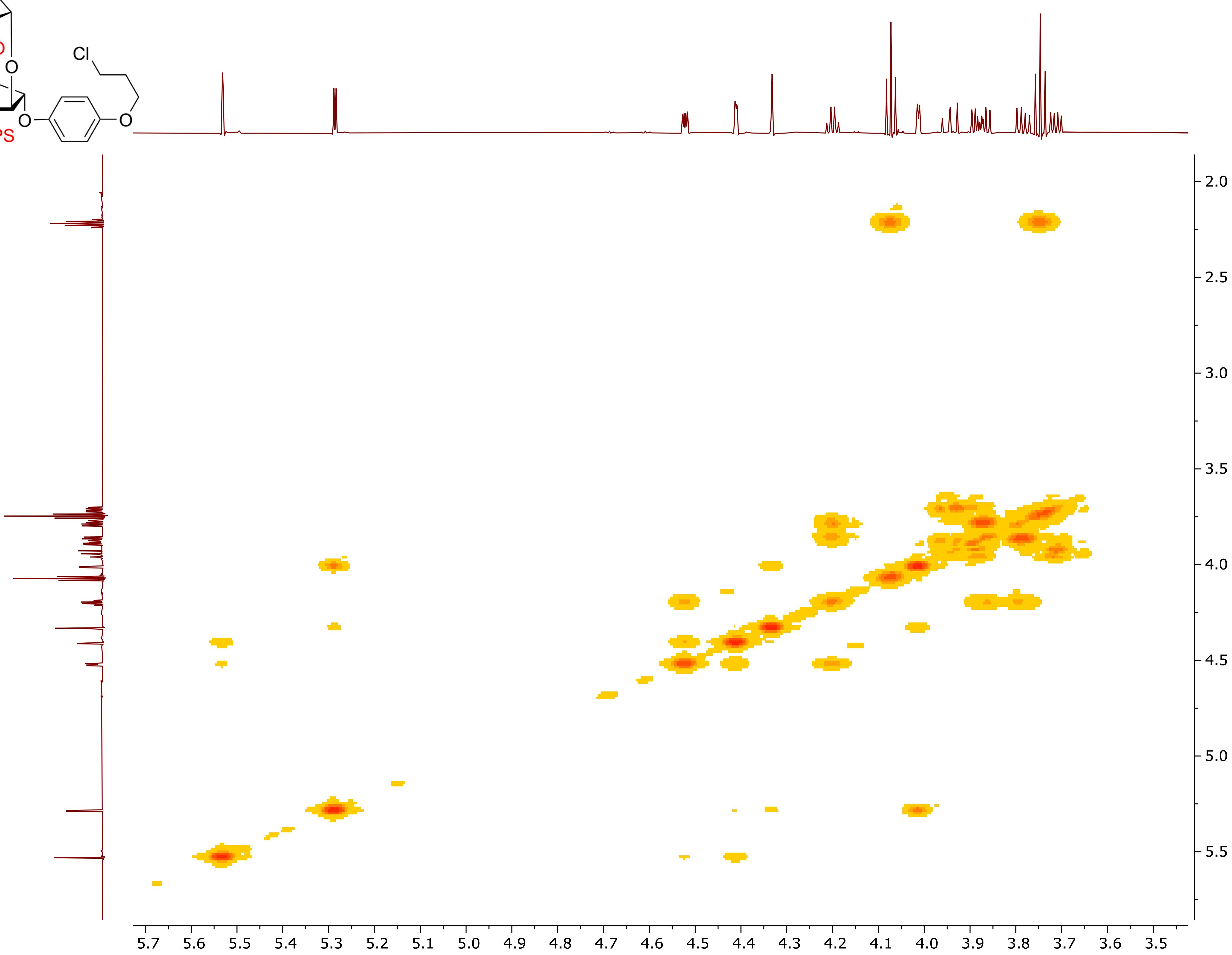

## HSQC (600 MHz) spectrum of compound 13 in $CDCl_3$

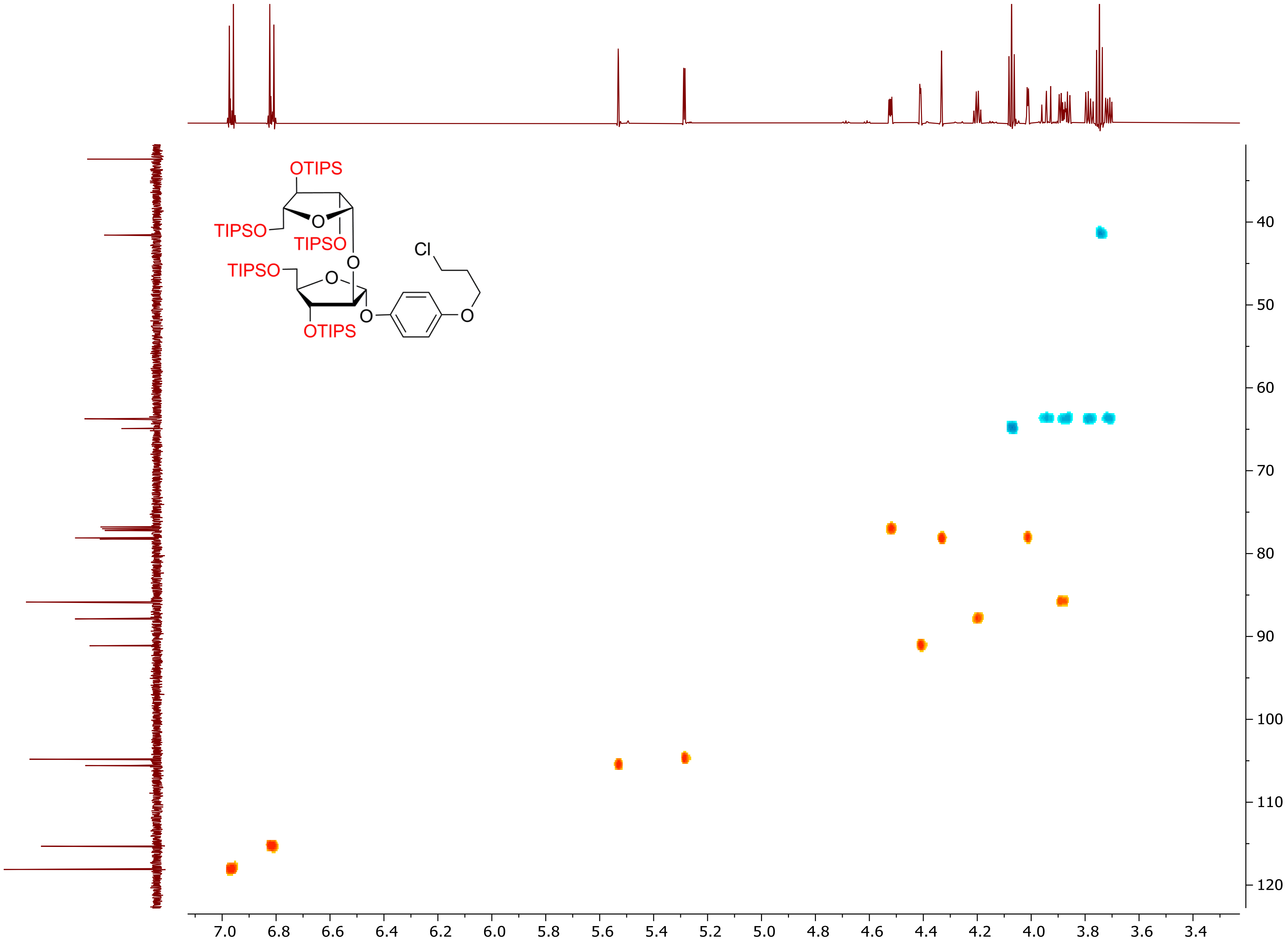

## HMBC (600 MHz) spectrum of compound 13 in $CDCl_3$

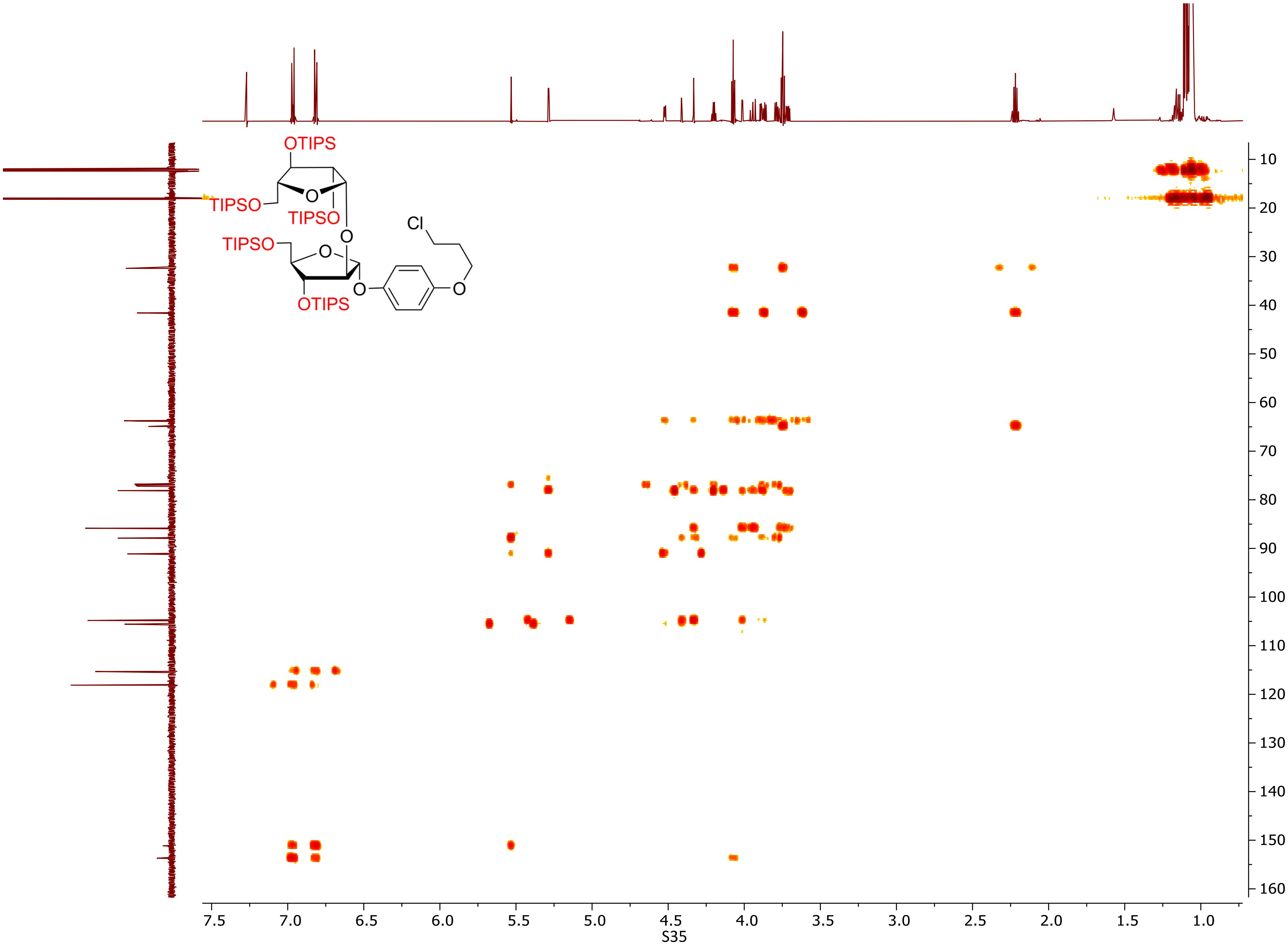

## $^{1}H$–$^{29}Si$ HMBC (300 MHz) spectrum of compound 13 in $CDCl_3$

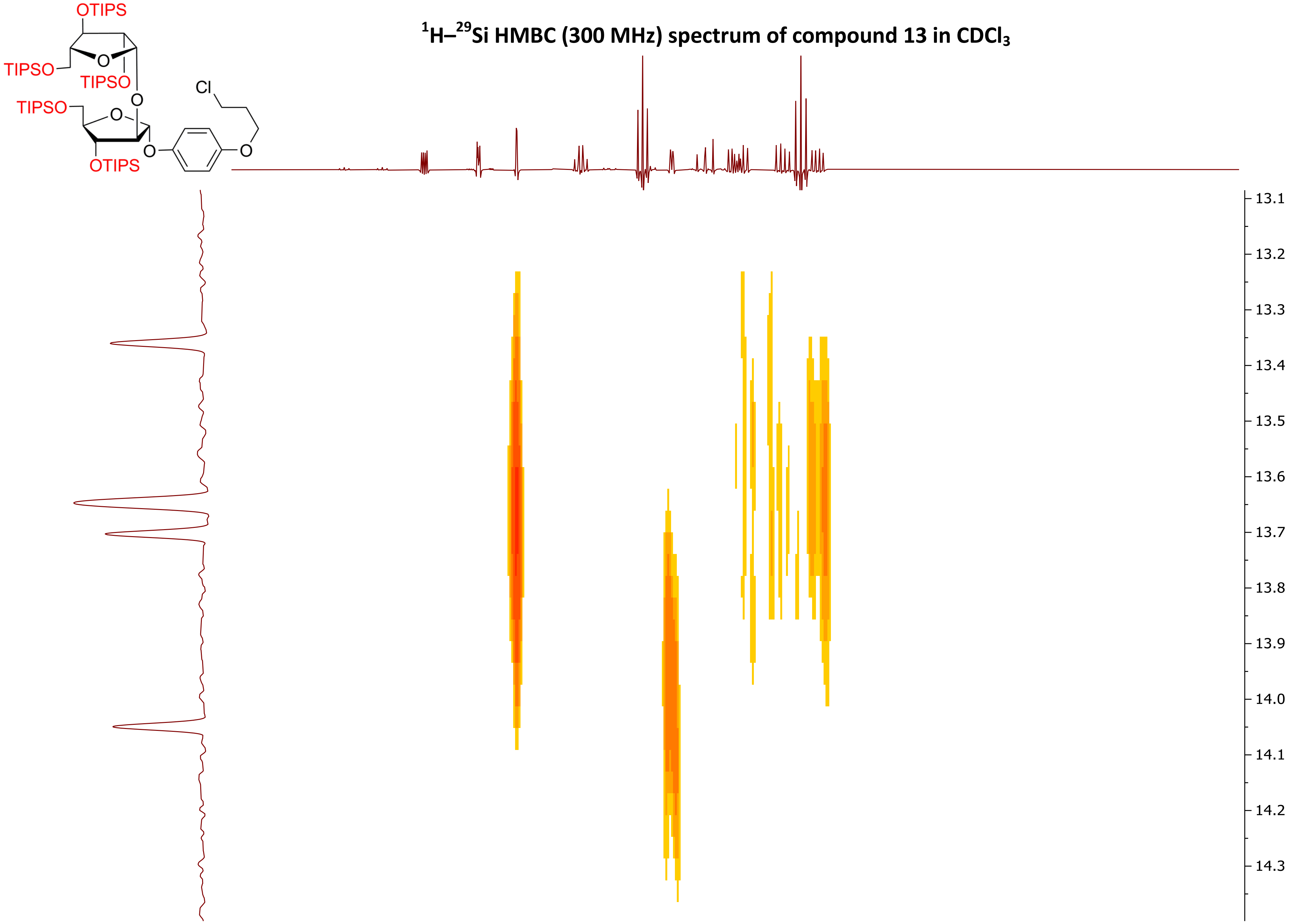

# $^{29}$Si INEPT NMR (59.6 MHz) spectrum of compound 13 in $CDCl_3$

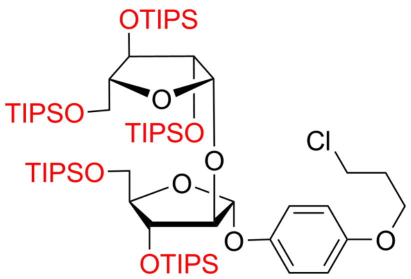


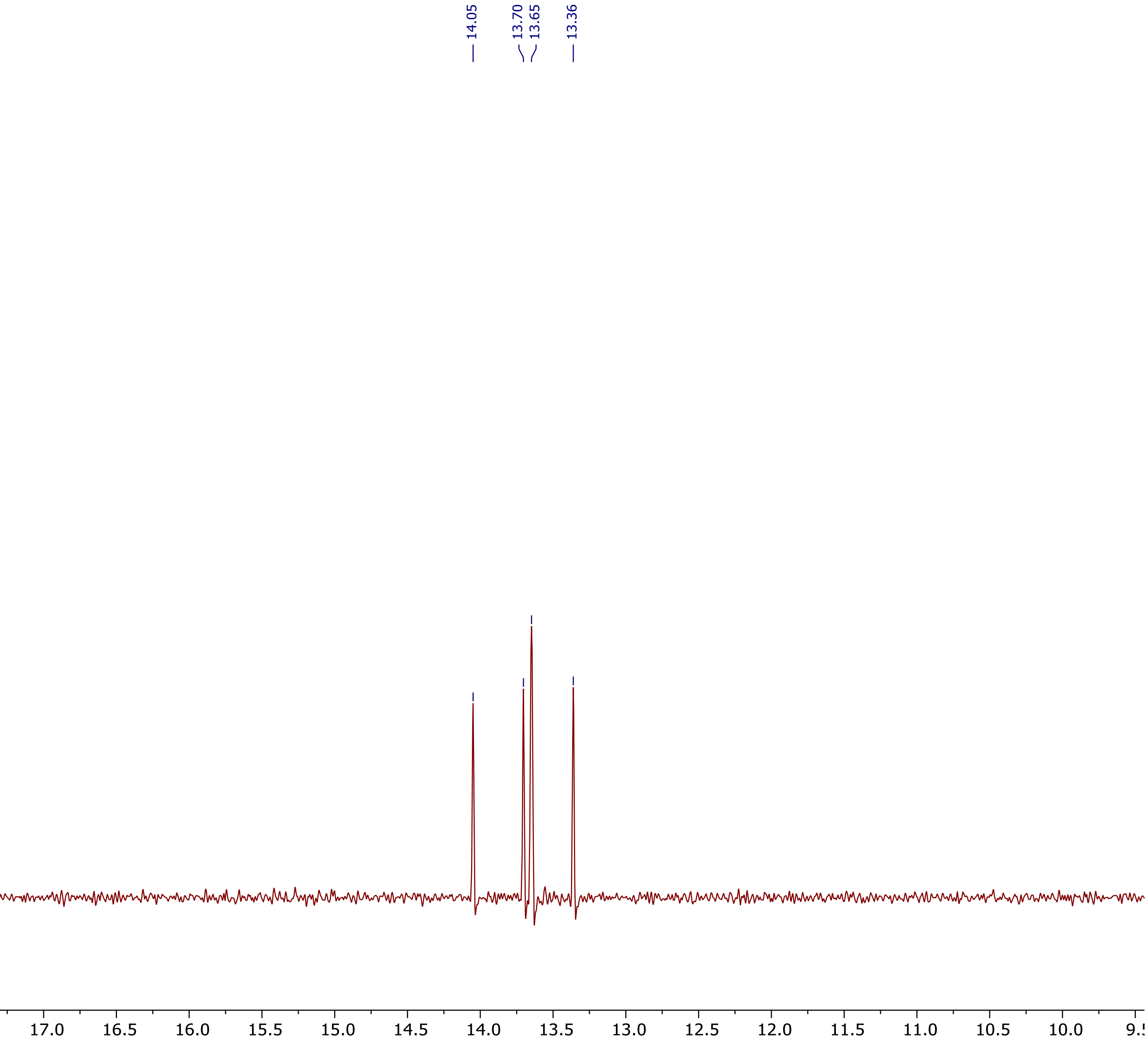

# $^1$H NMR (600 MHz) spectrum of compound 14 in $CDCl_3$

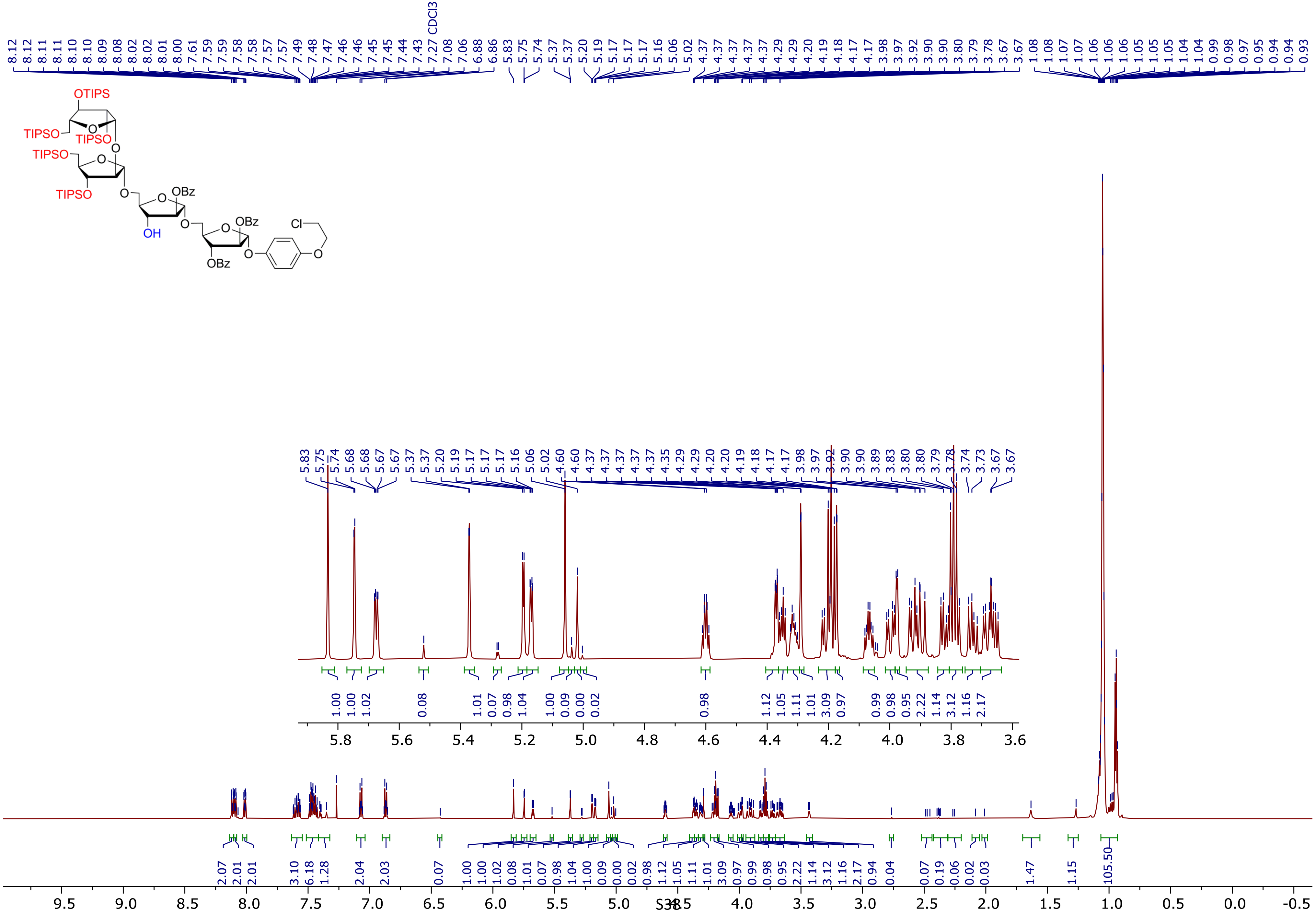

# $^{13}C$ NMR (151 MHz) spectrum of compound 14 in $CDCl_3$

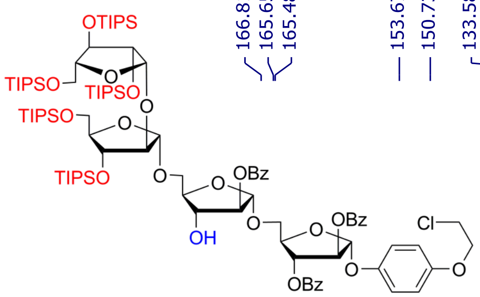


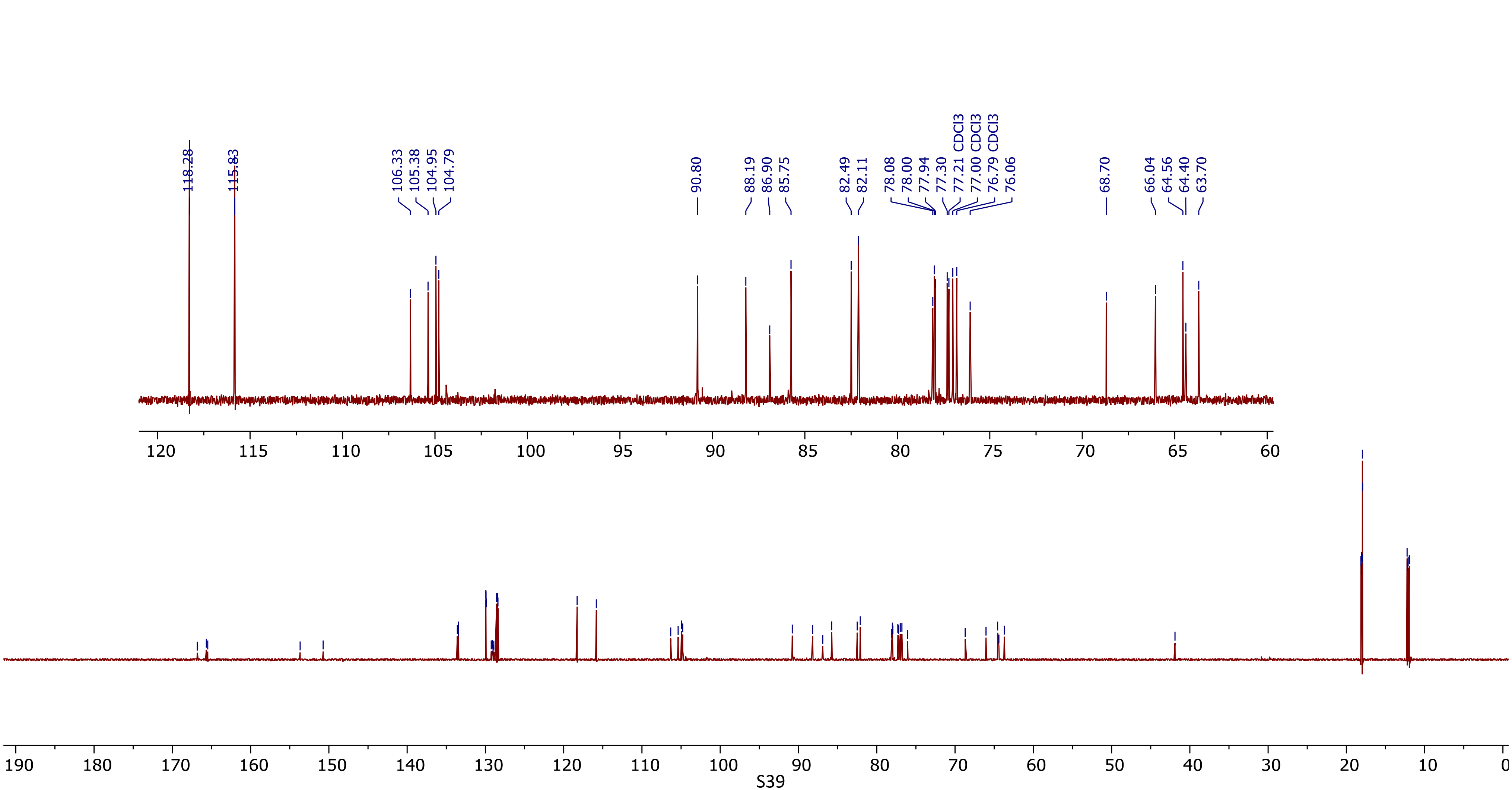

## COSY (600 MHz) spectrum of compound 14 in $CDCl_3$

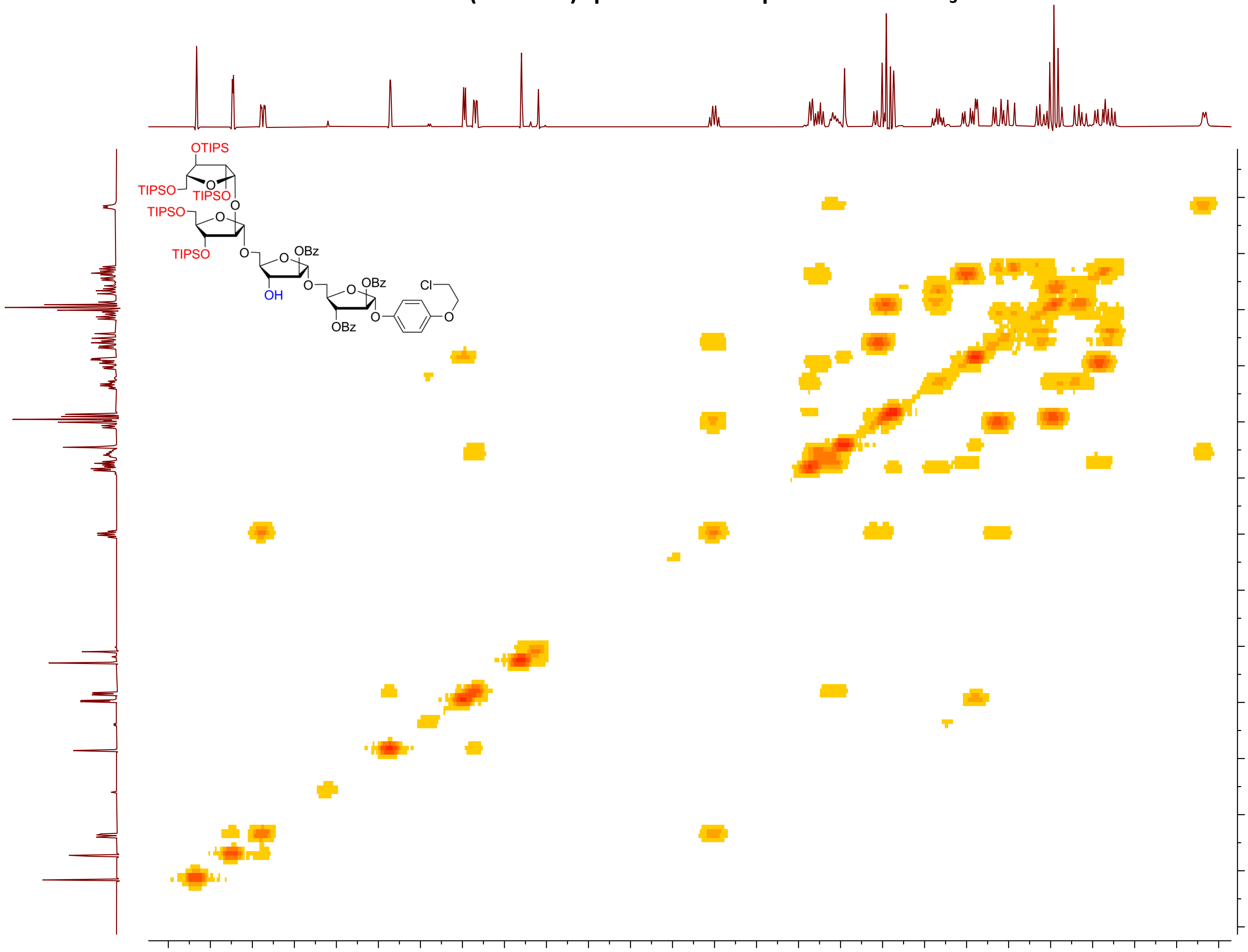

HSQC (600 MHz) spectrum of compound 14 in $CDCl_3$

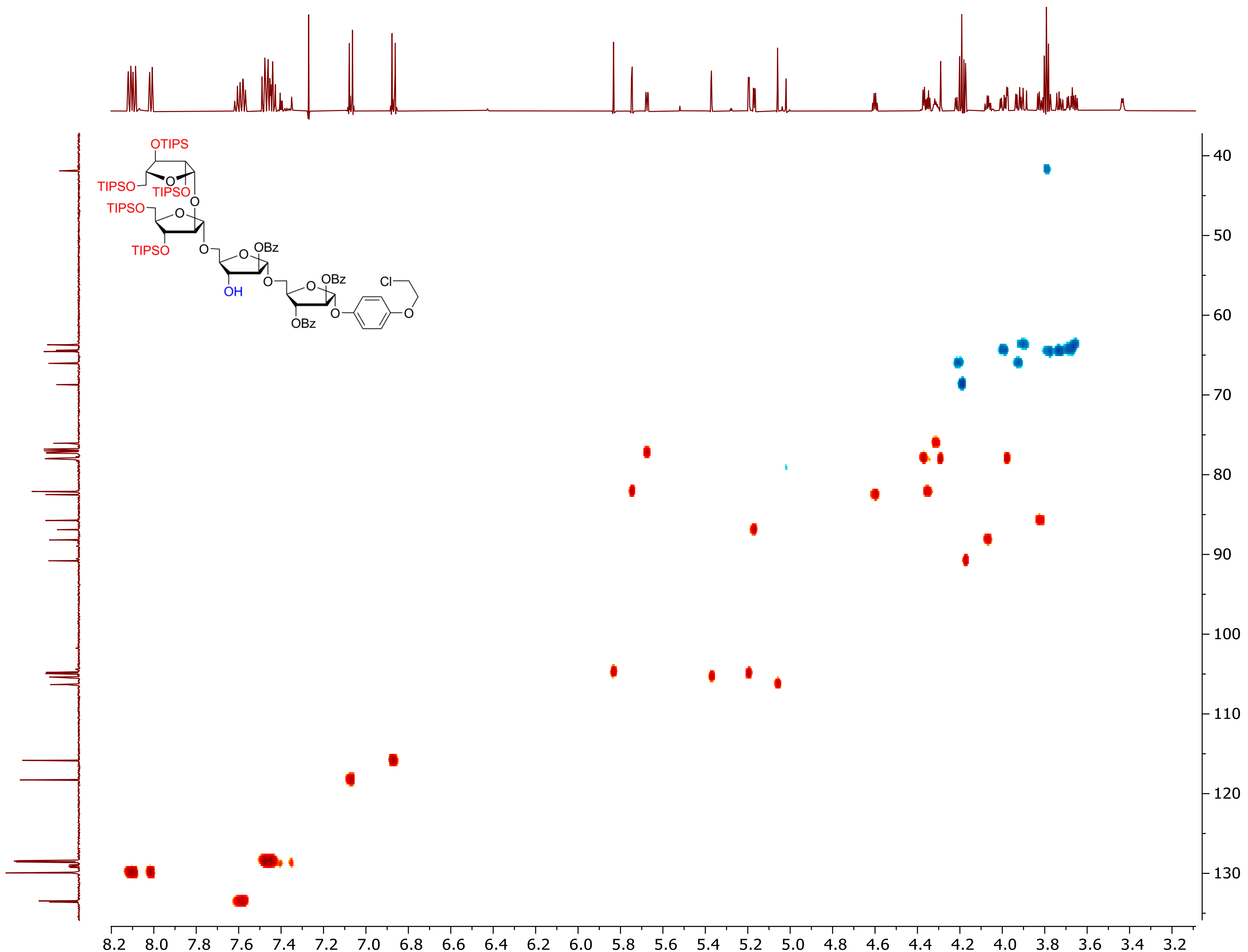

# HMBC (600 MHz) spectrum of compound 14 in $CDCl_3$

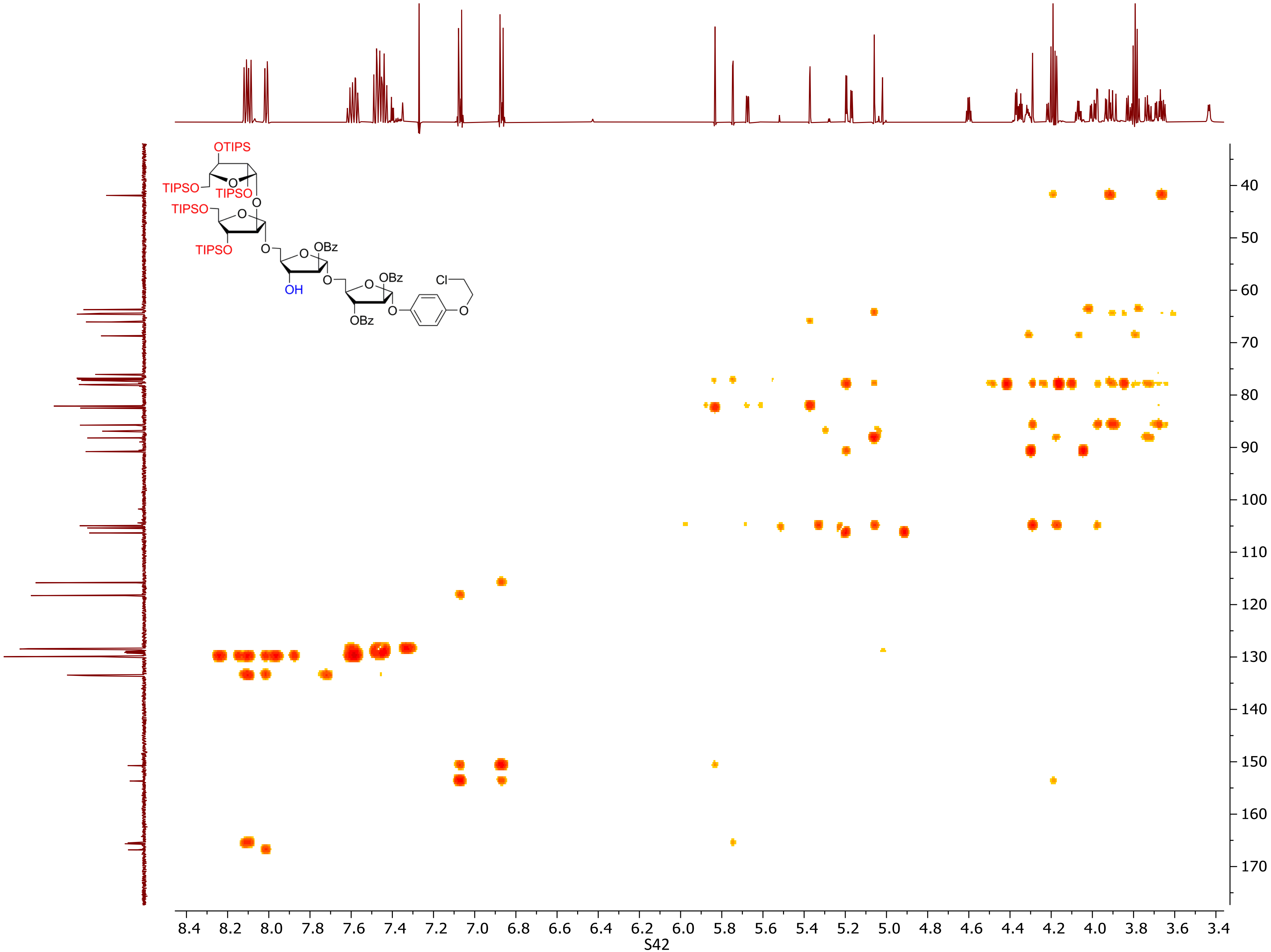

# $^1$H NMR (300 MHz) spectrum of compounds 15+16 in $CDCl_3$

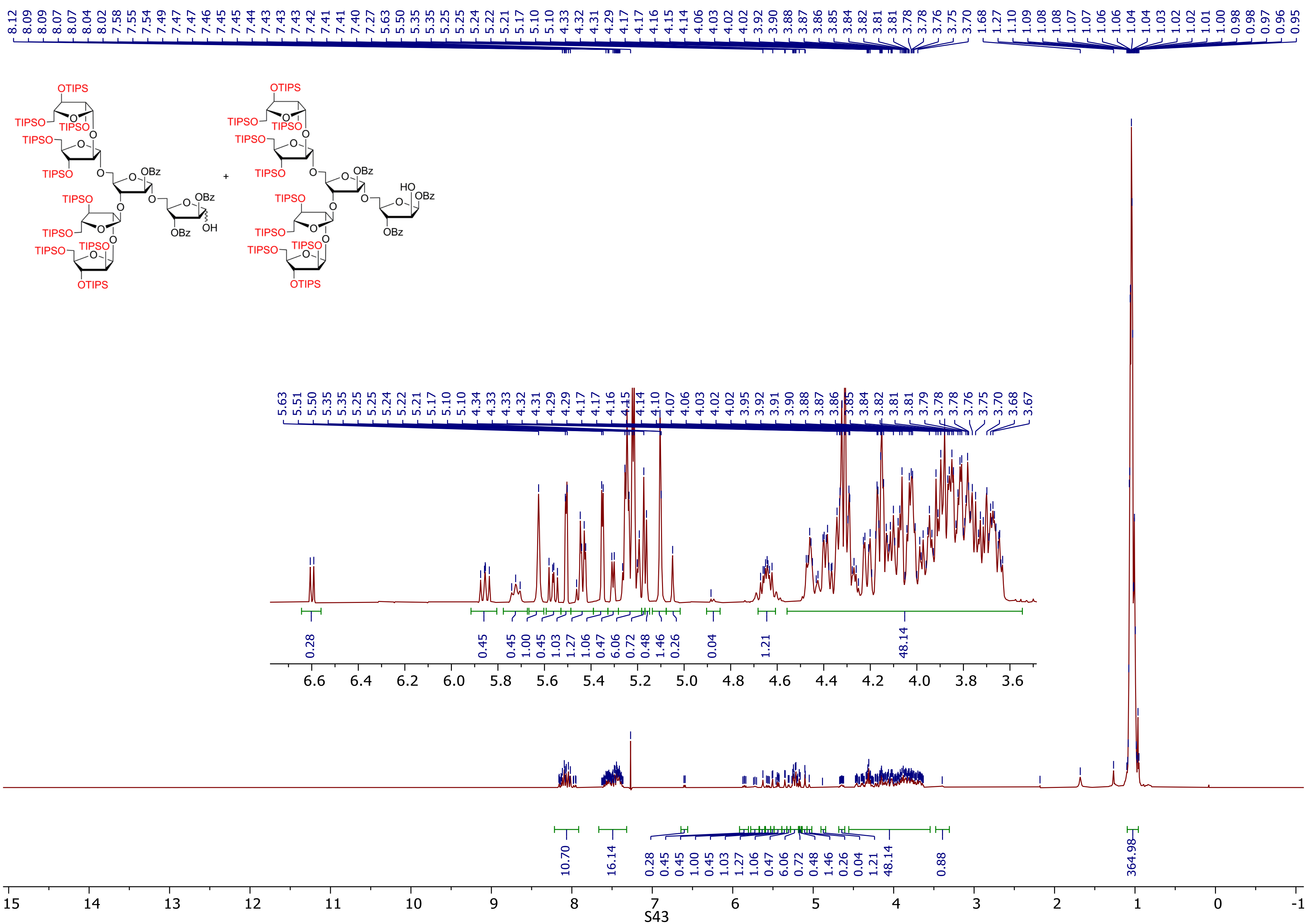

**$^{13}C$ NMR (151 MHz) spectrum of compounds 15+16 in $CDCl_3$**

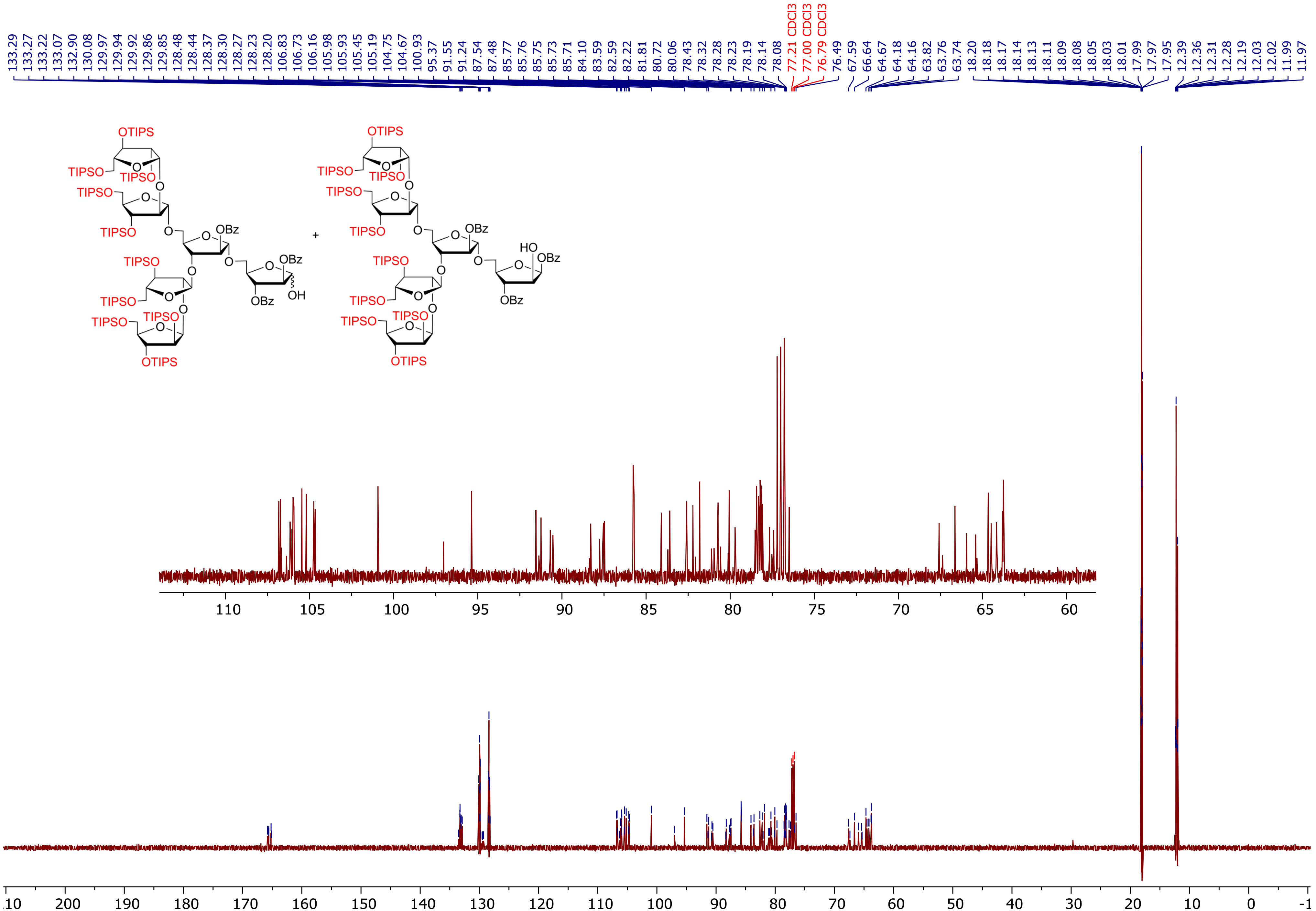

## COSY (600 MHz) spectrum of compounds 15+16 in $CDCl_3$

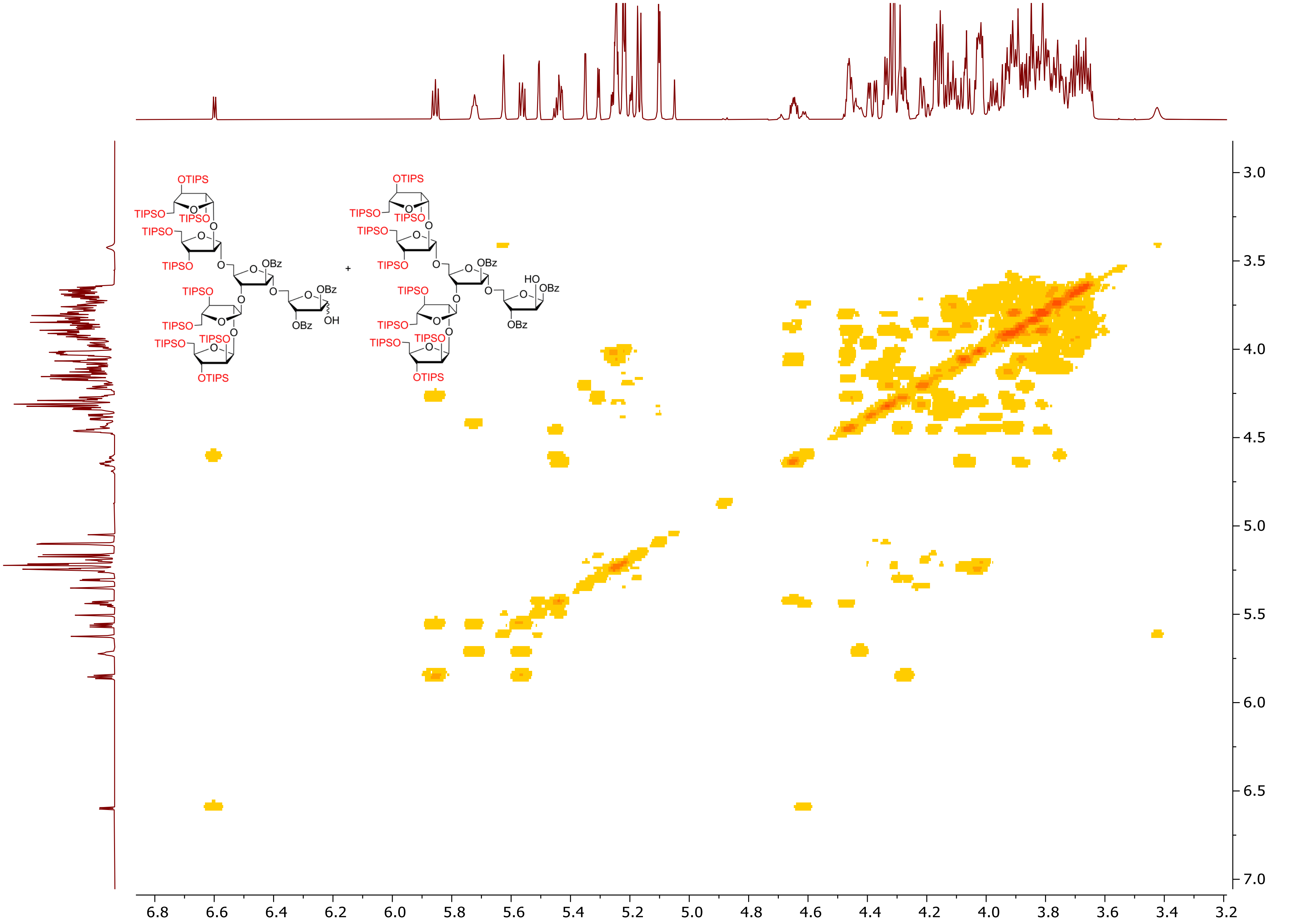

## HSQC (600 MHz) spectrum of compounds 15+16 in $CDCl_3$

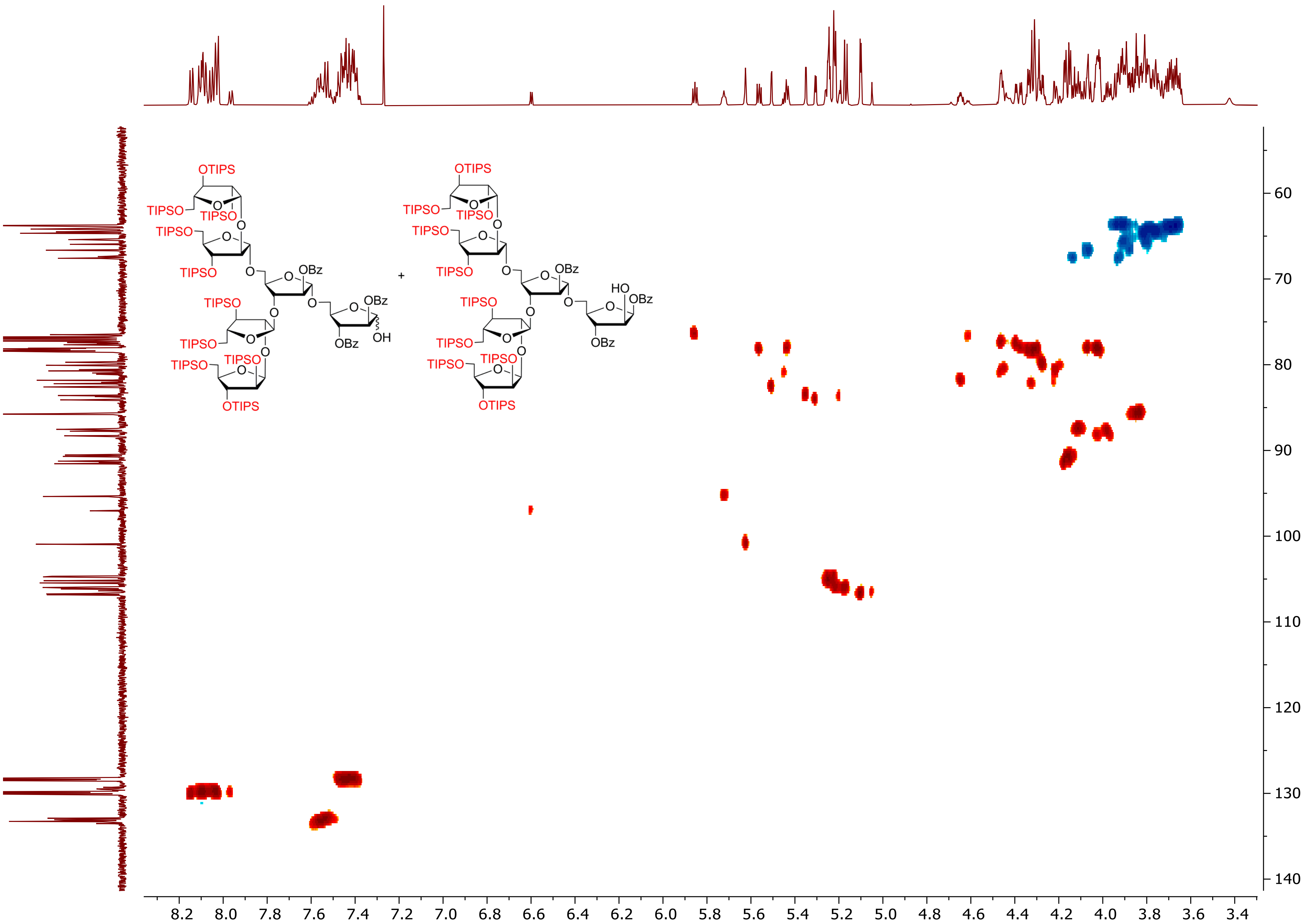

## HMBC (600 MHz) spectrum of compounds 15+16 in $CDCl_3$

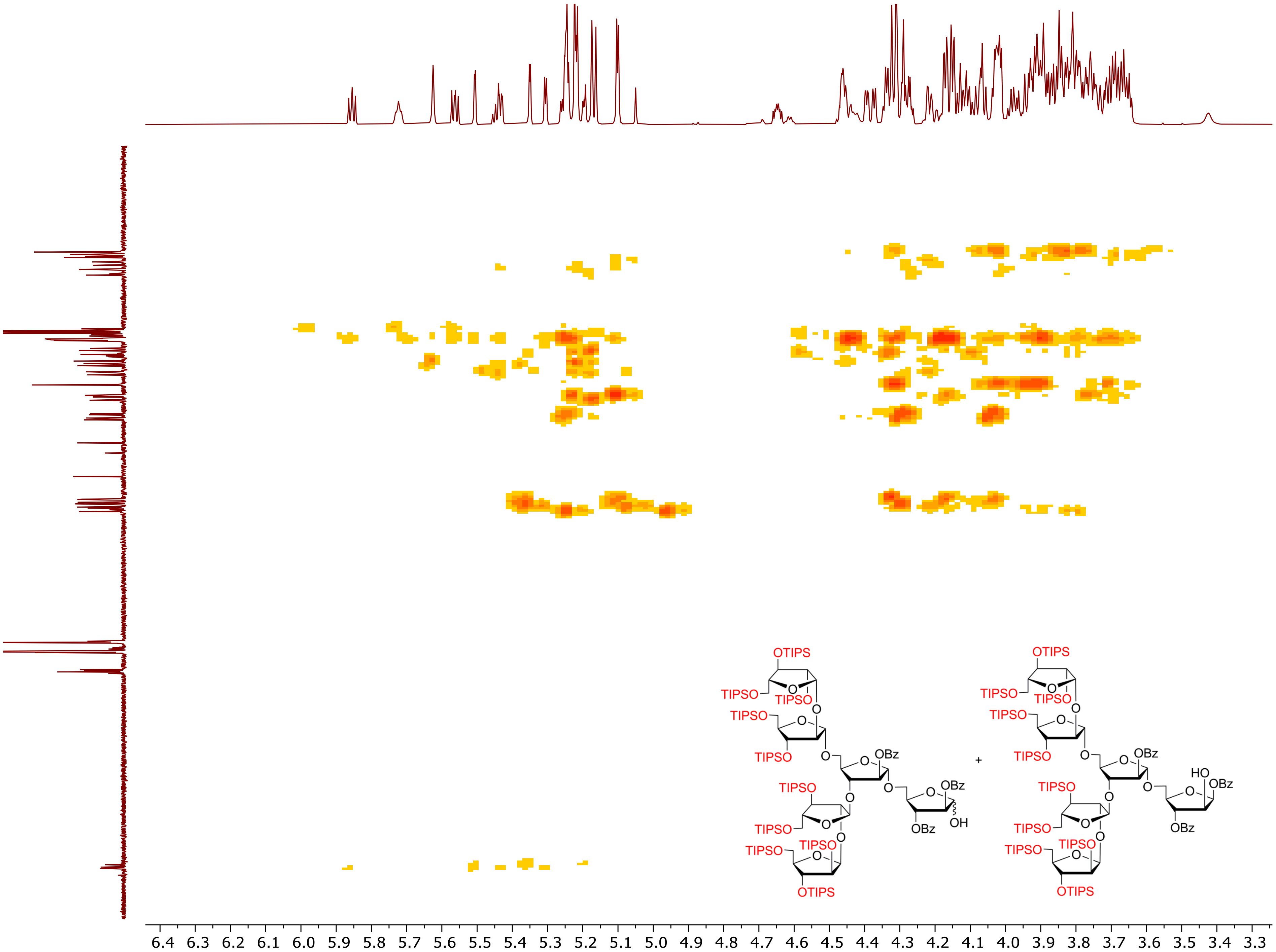

# $^{1}H$ NMR (600 MHz) spectrum of compound 18 in $CDCl_3$

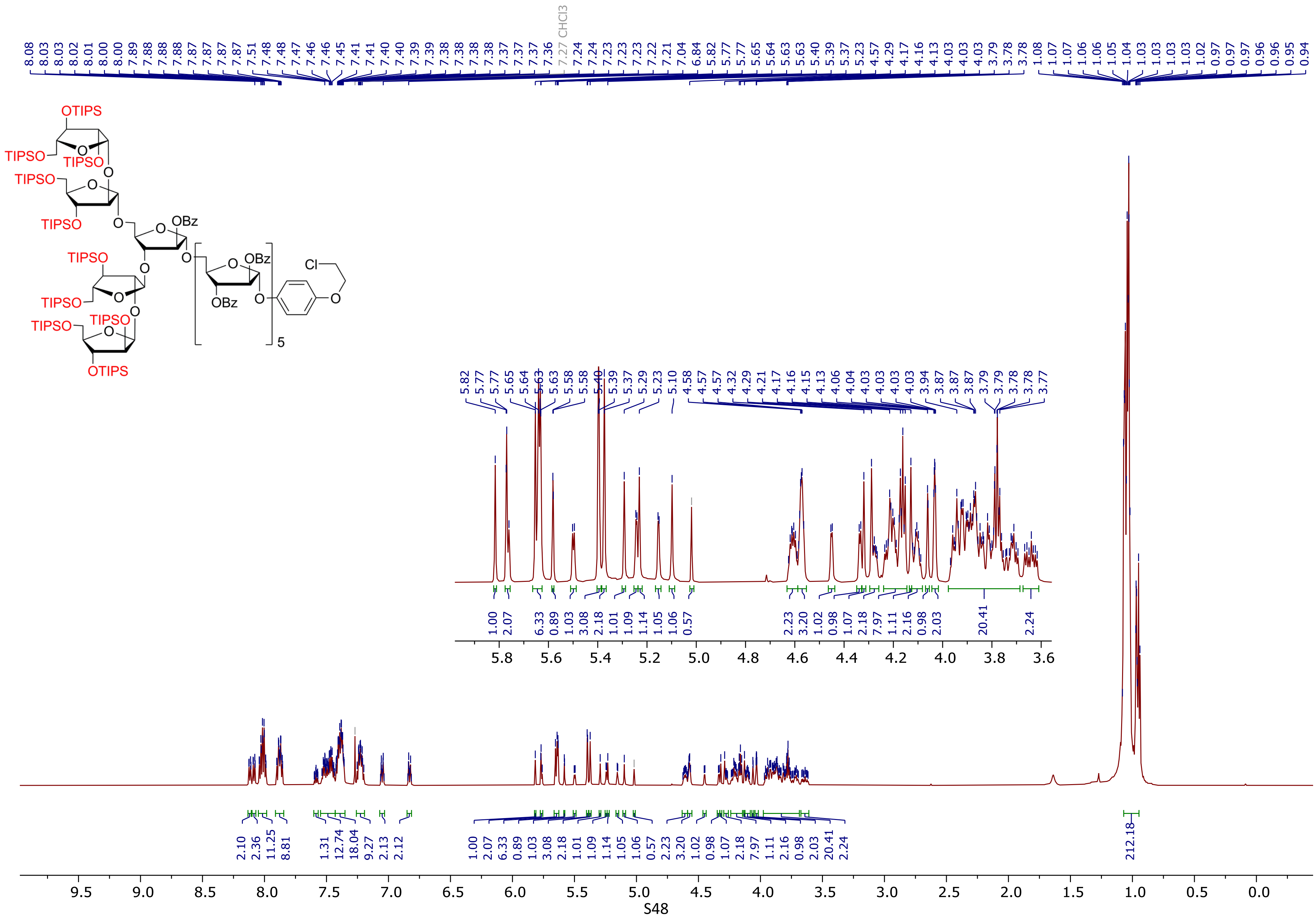

# $^{13}C$ NMR (151 MHz) spectrum of compound 18 in $CDCl_3$

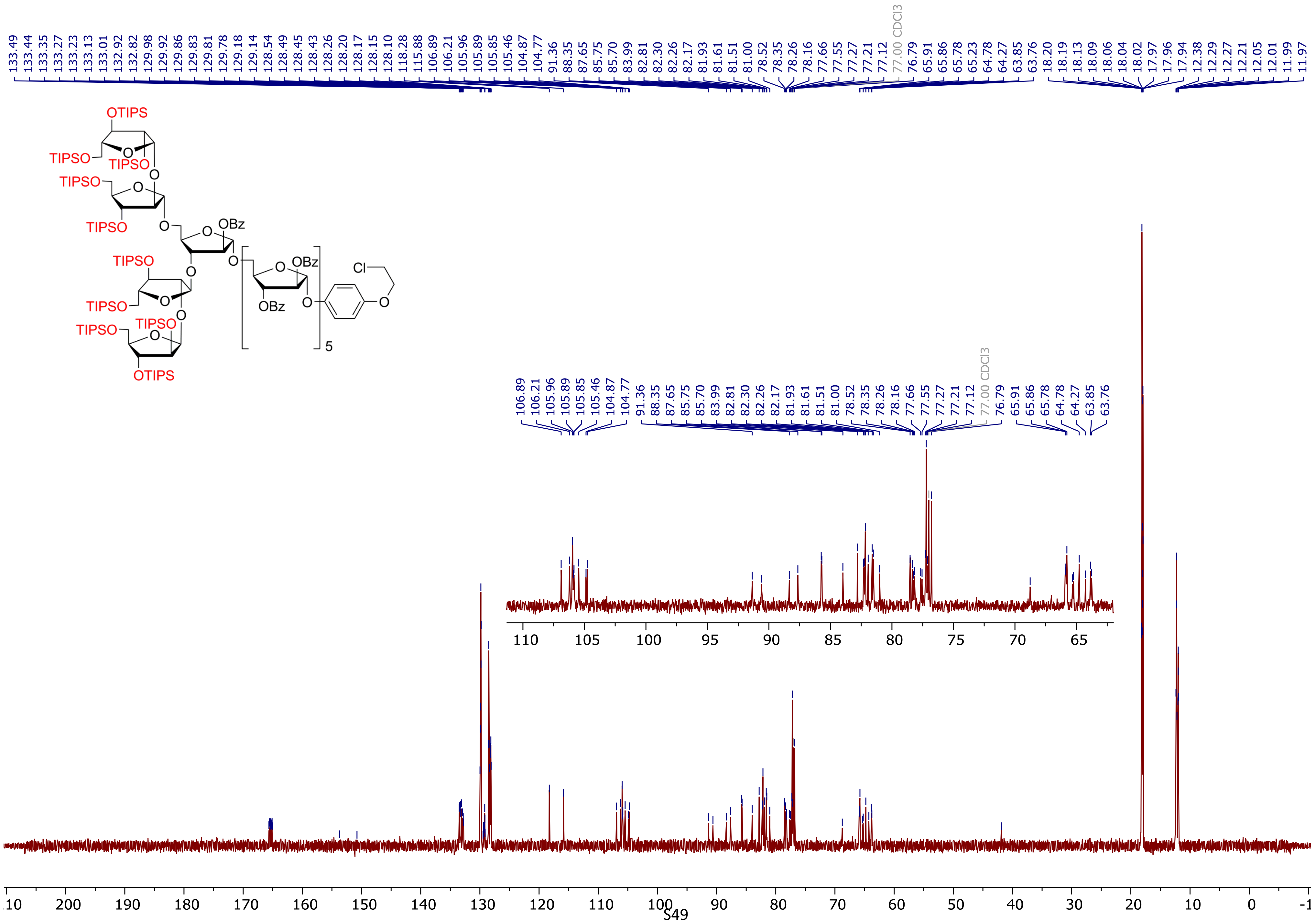

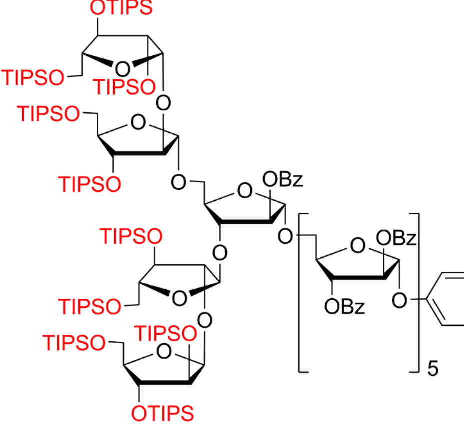


## COSY (600 MHz) spectrum of compound 18 in $CDCl_3$

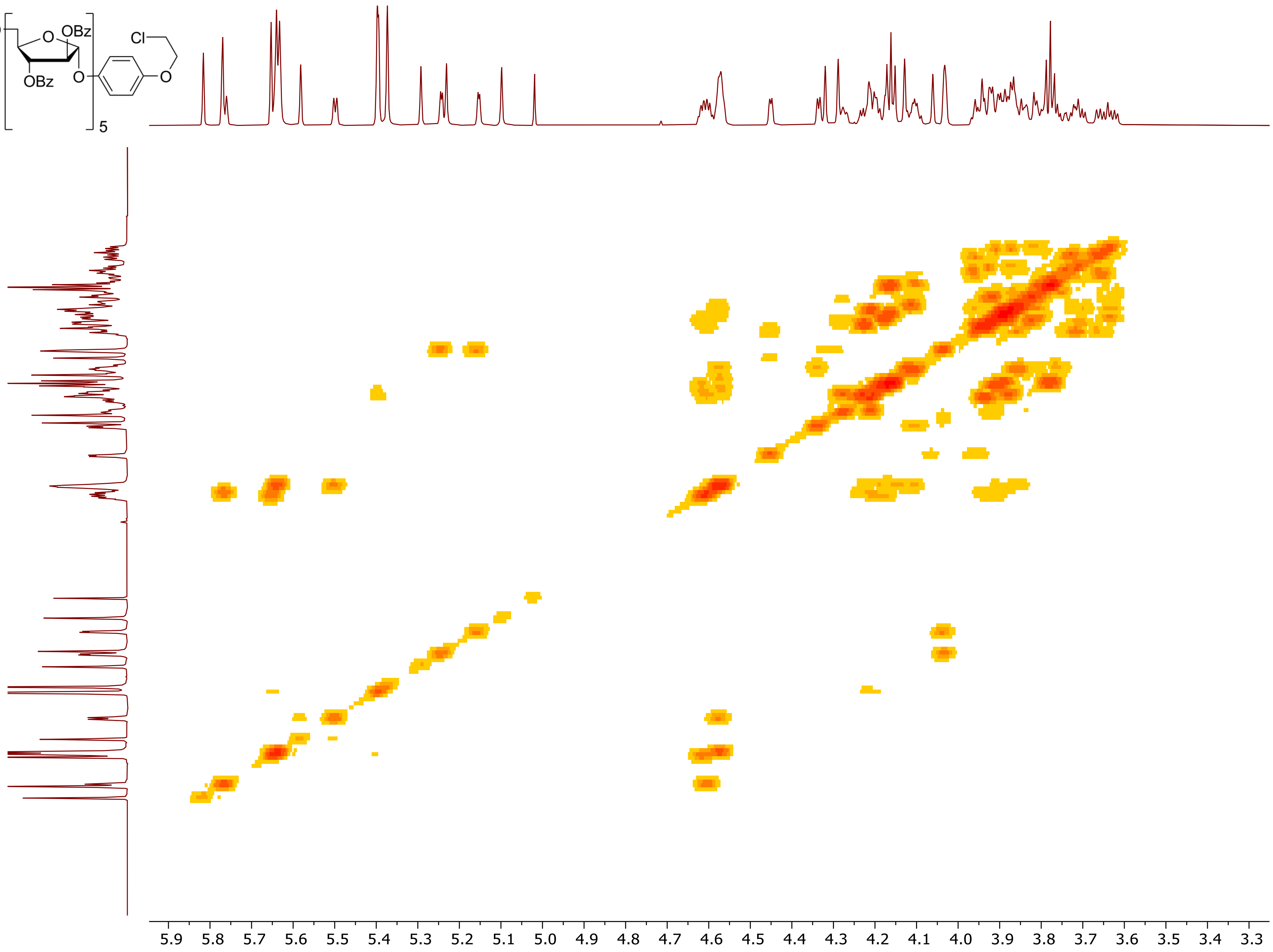

## HSQC (600 MHz) spectrum of compound 18 in $CDCl_3$

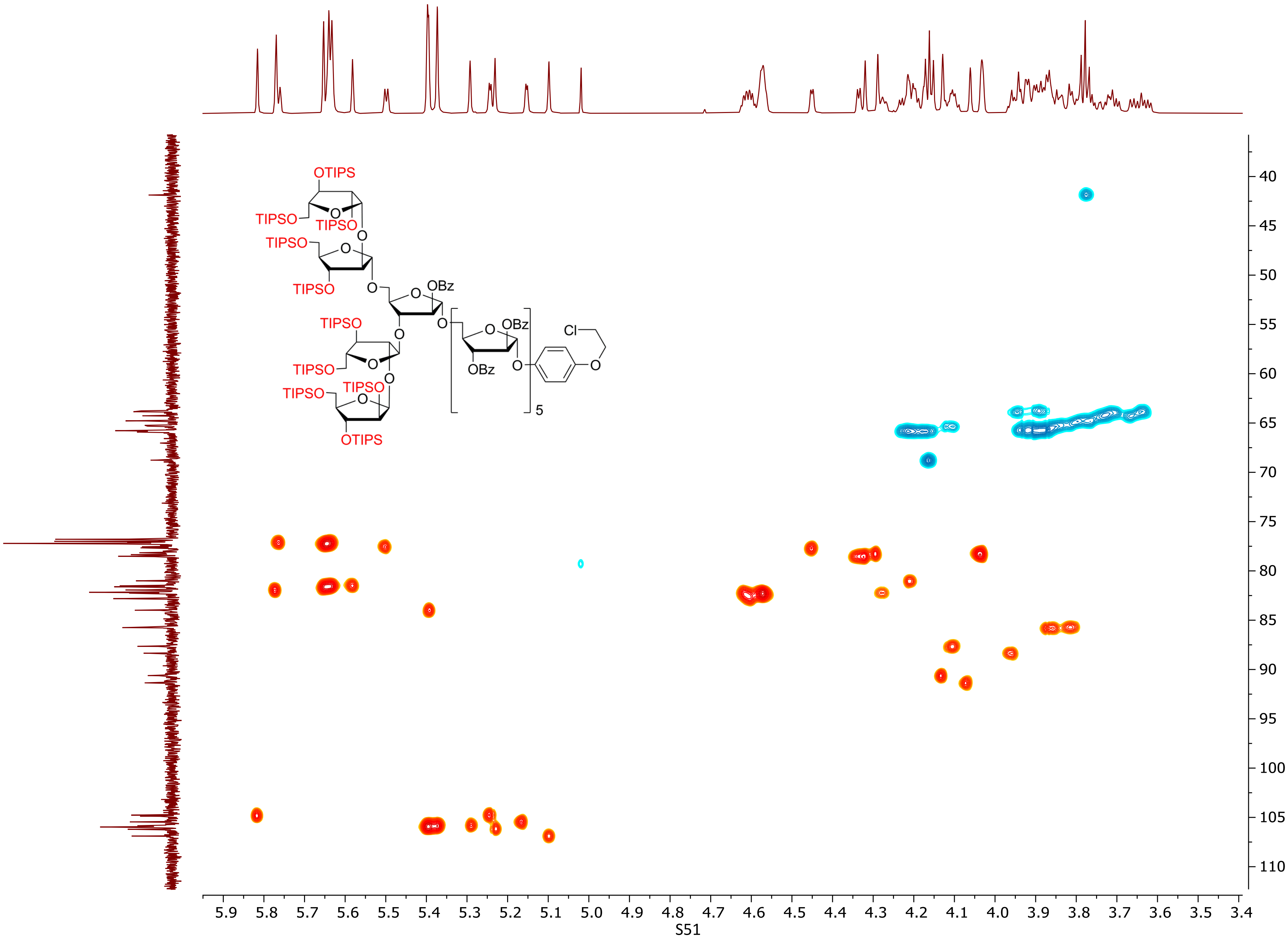

HMBC (600 MHz) spectrum of compound 18 in $CDCl_3$

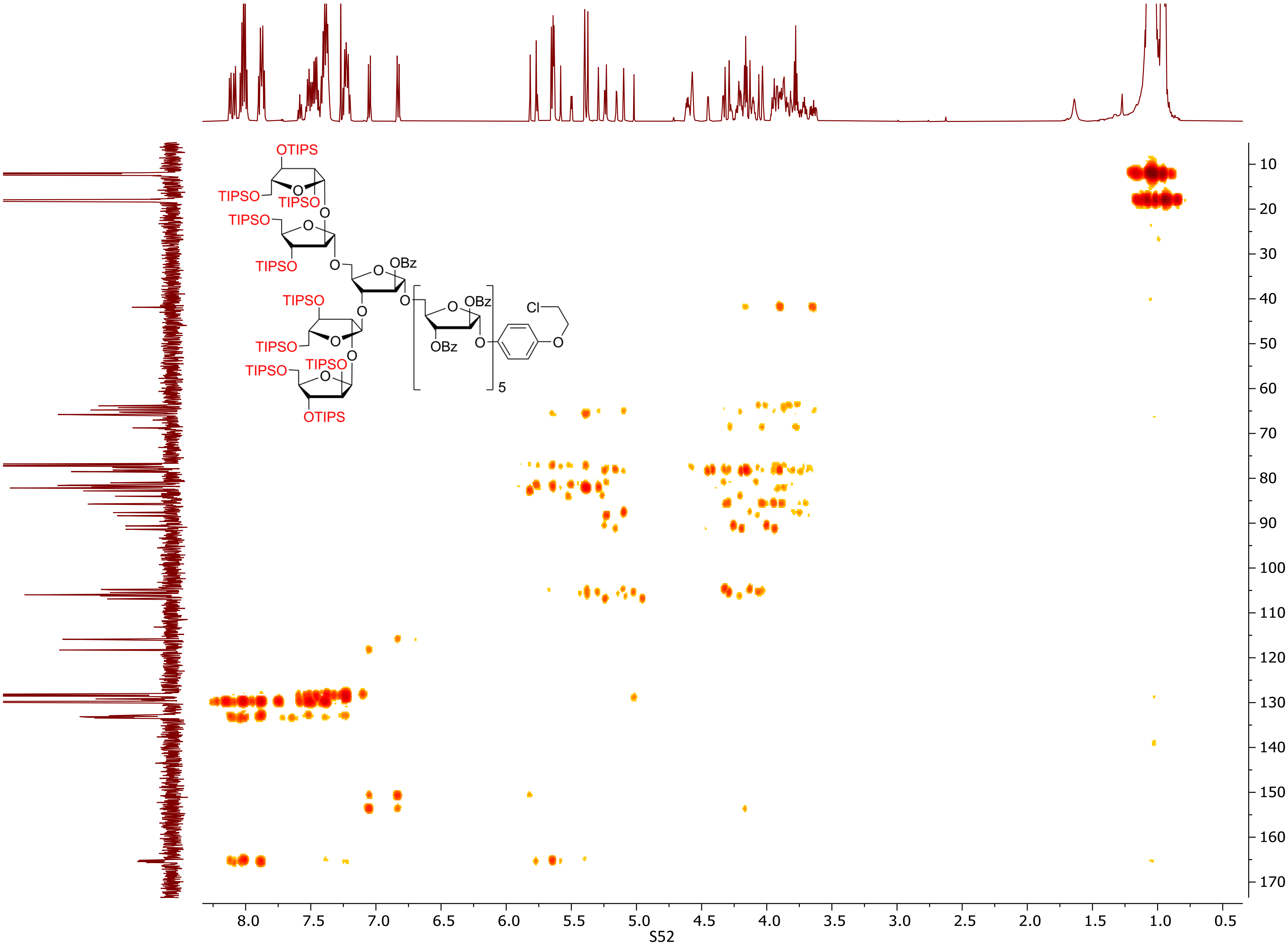

## $^{1}H$–$^{29}Si$ HMBC (300 MHz) spectrum of compound 18 in $CDCl_3$

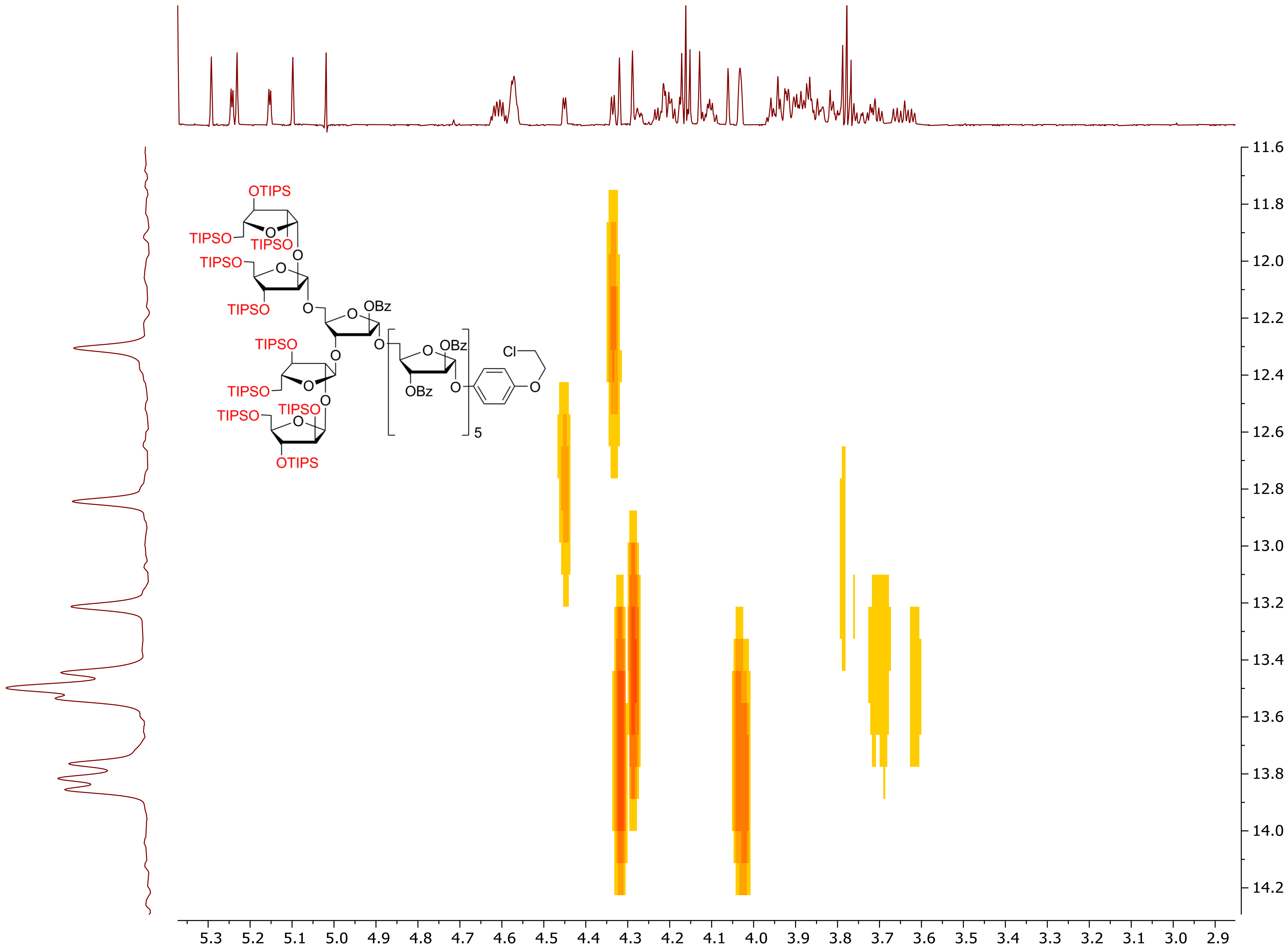

## $^{29}$Si INEPT NMR (59.6 MHz) spectrum of compound 18 in $CDCl_3$

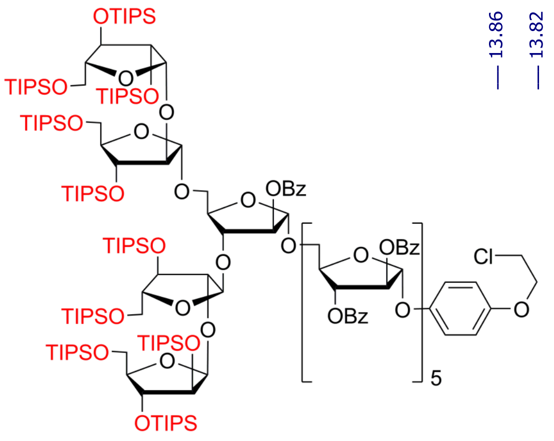


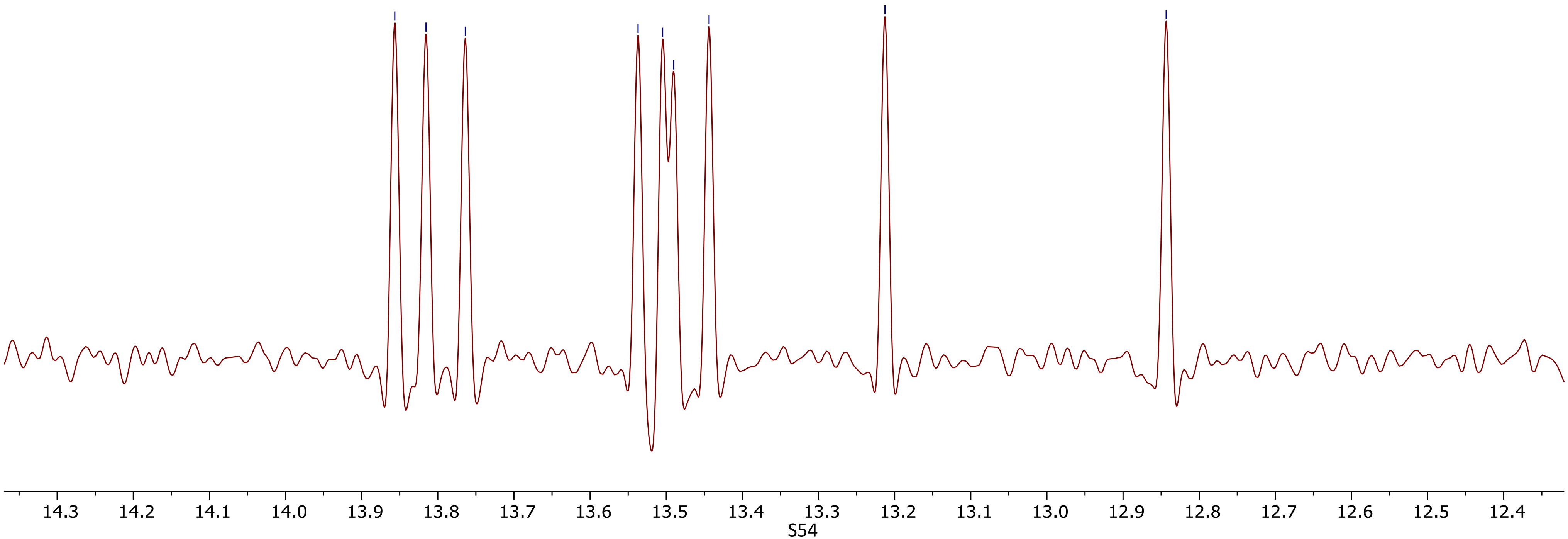

# $^{1}H$ NMR (600 MHz) spectrum of compound 19 in $CDCl_3$

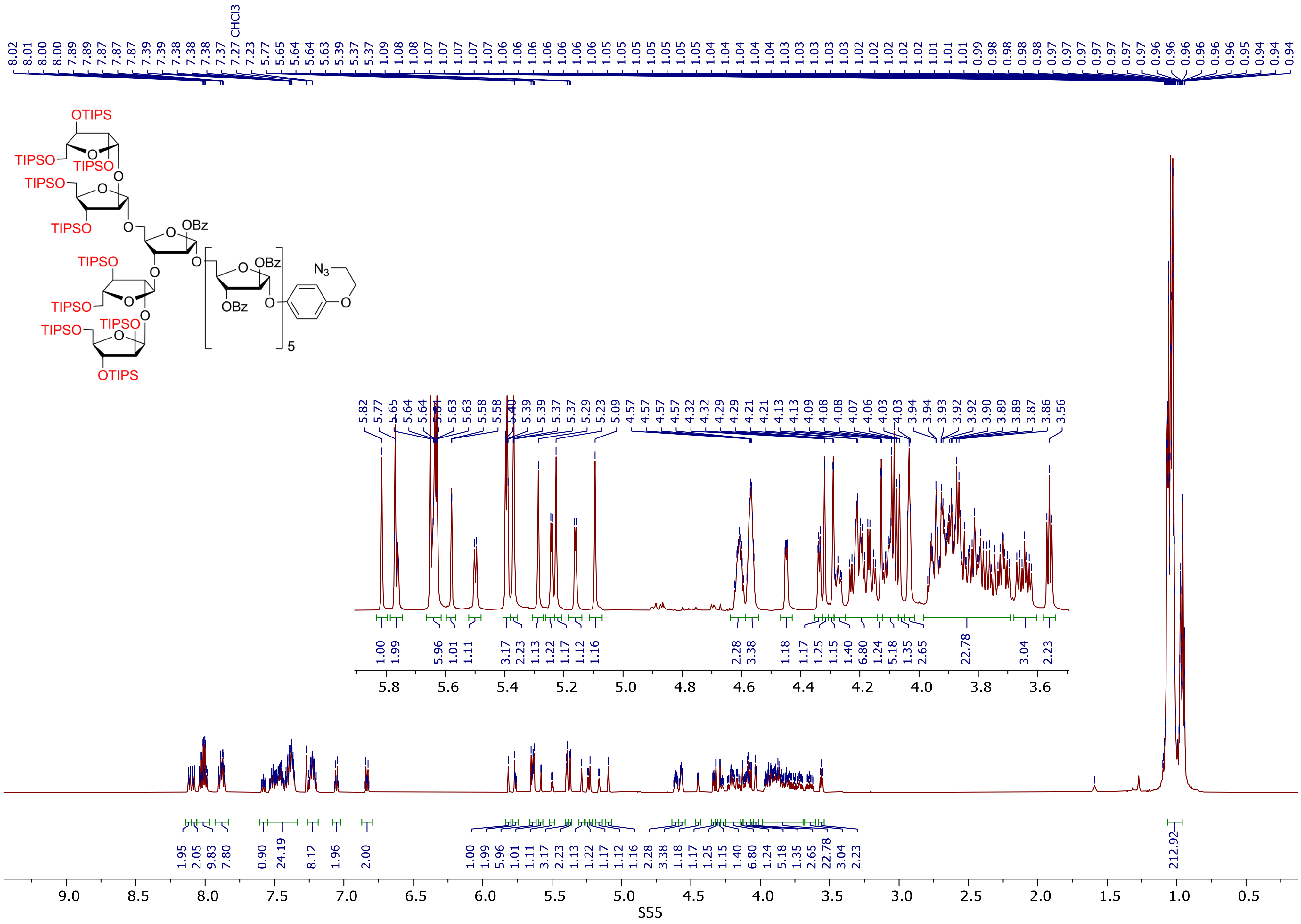

# $^{13}C$ NMR (151 MHz) spectrum of compound 19 in $CDCl_3$

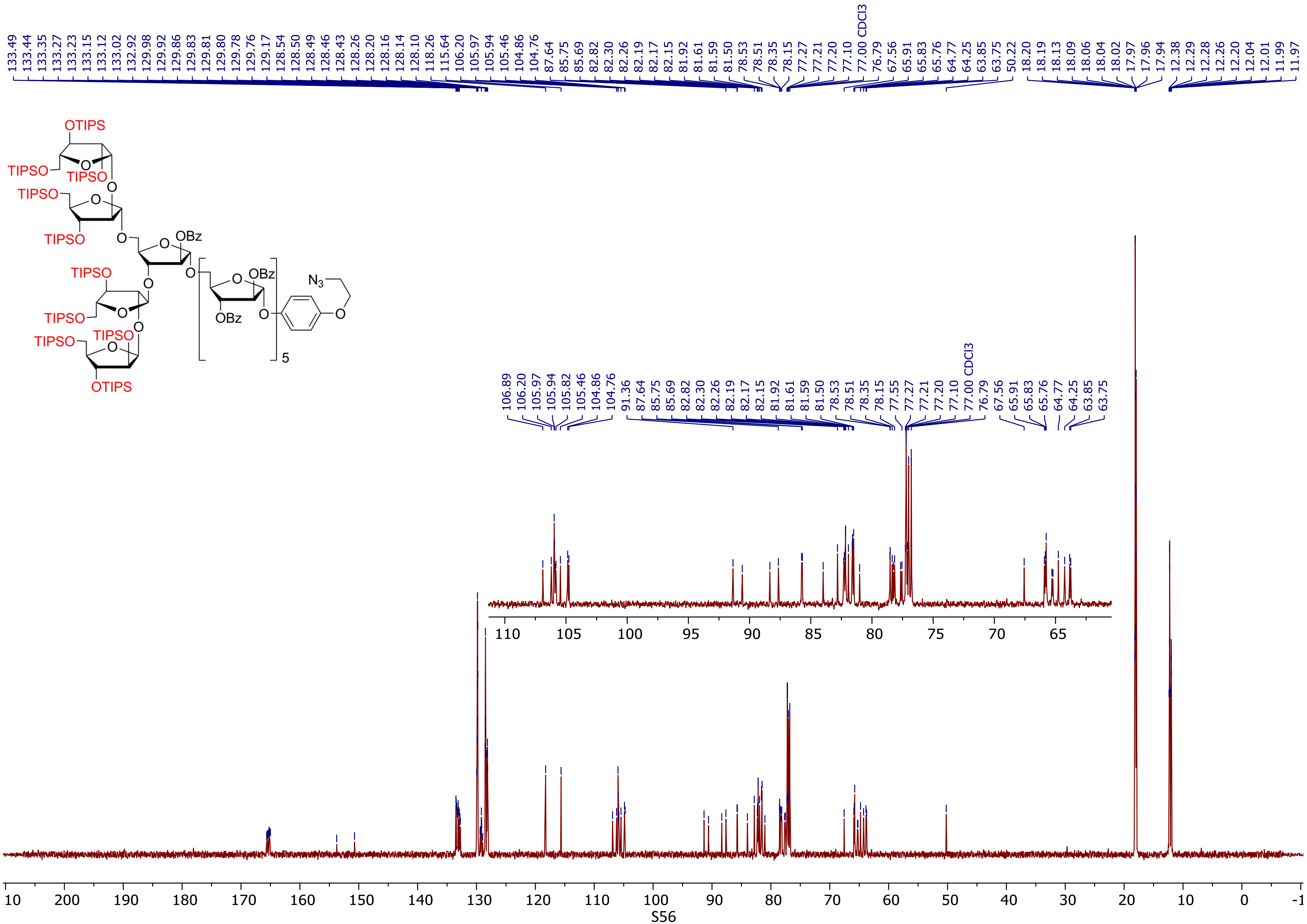

COSY (600 MHz) spectrum of compound 19 in $CDCl_3$

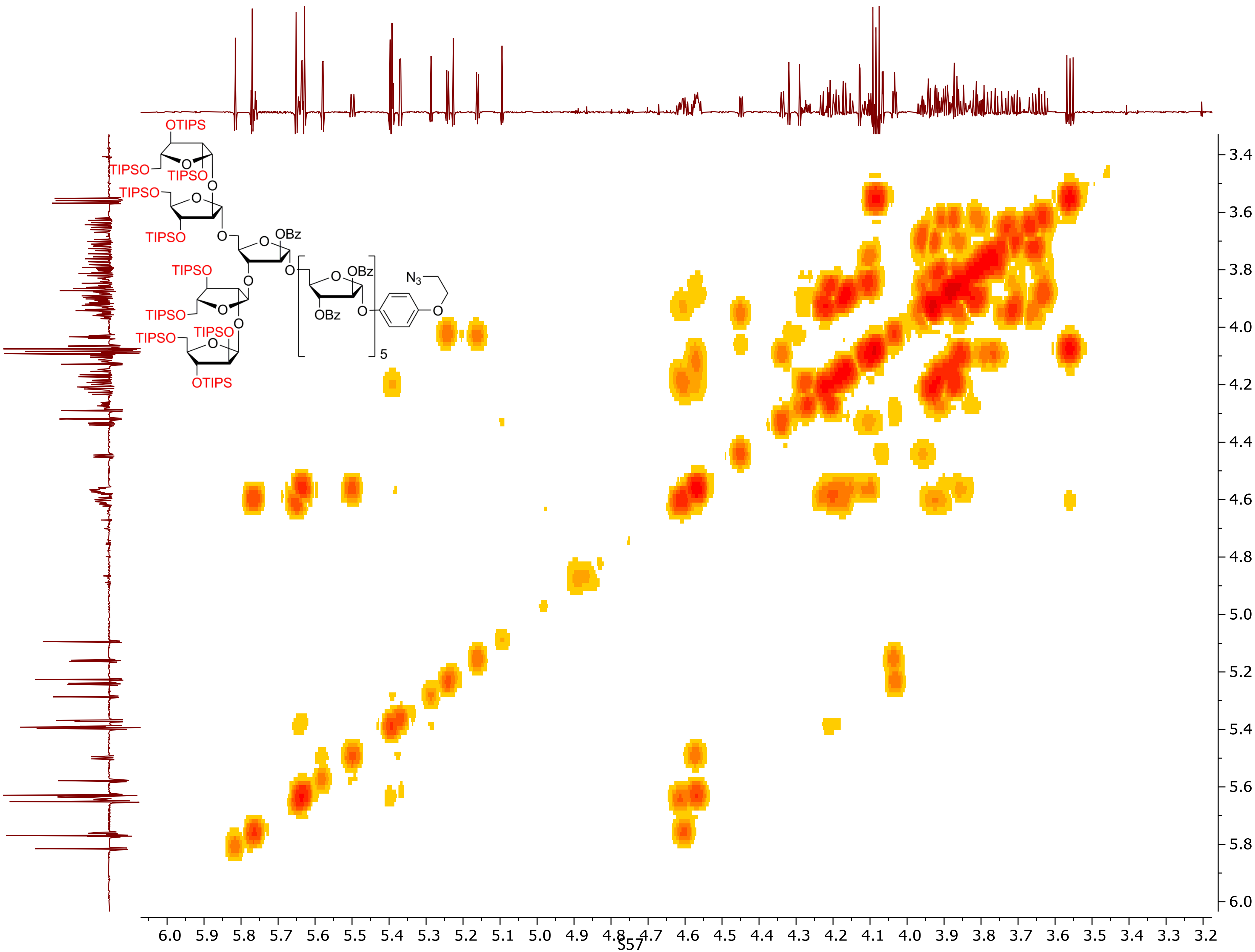

## HSQC (600 MHz) spectrum of compound 19 in $CDCl_3$

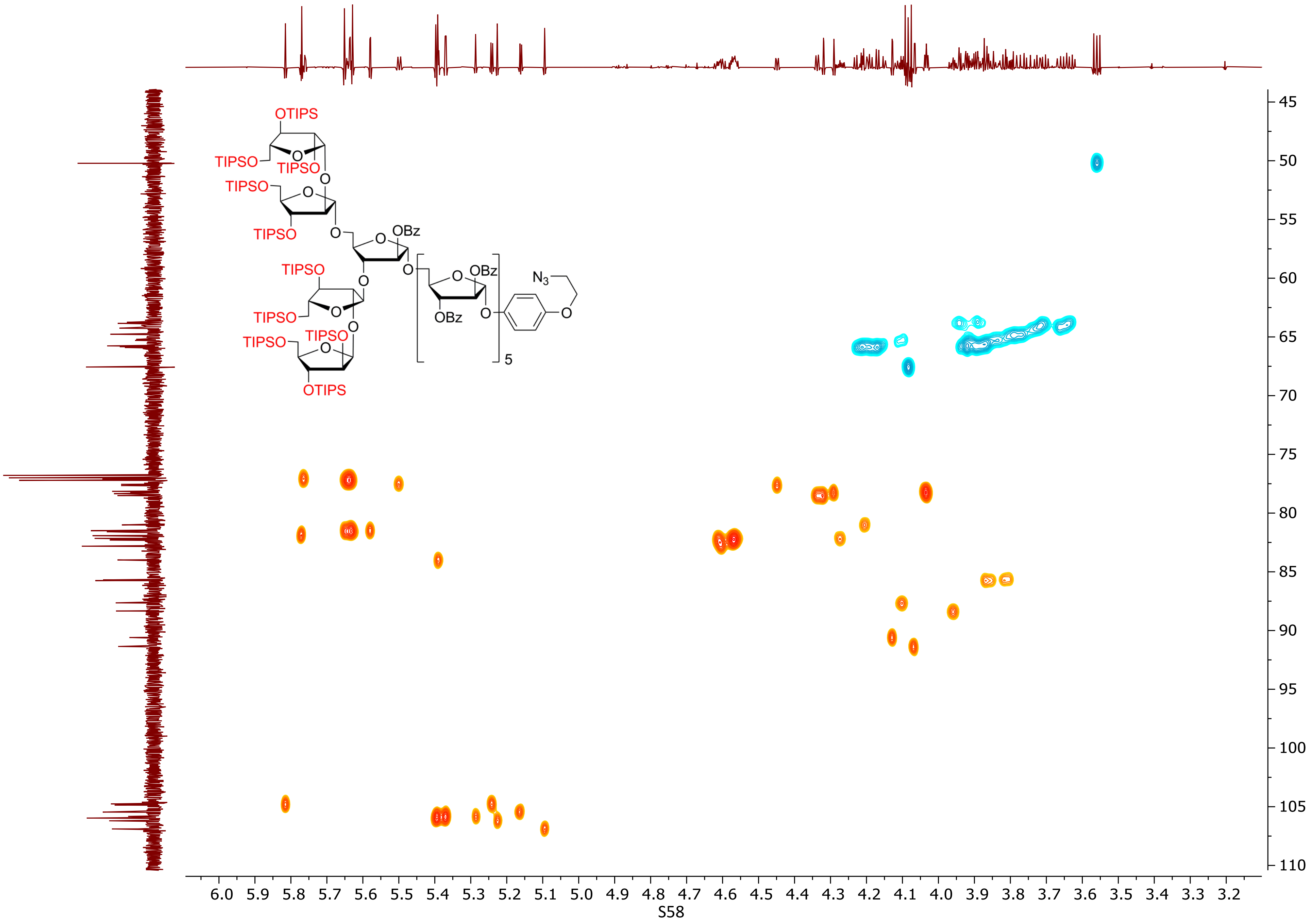

# HMBC (600 MHz) spectrum of compound 19 in $CDCl_3$

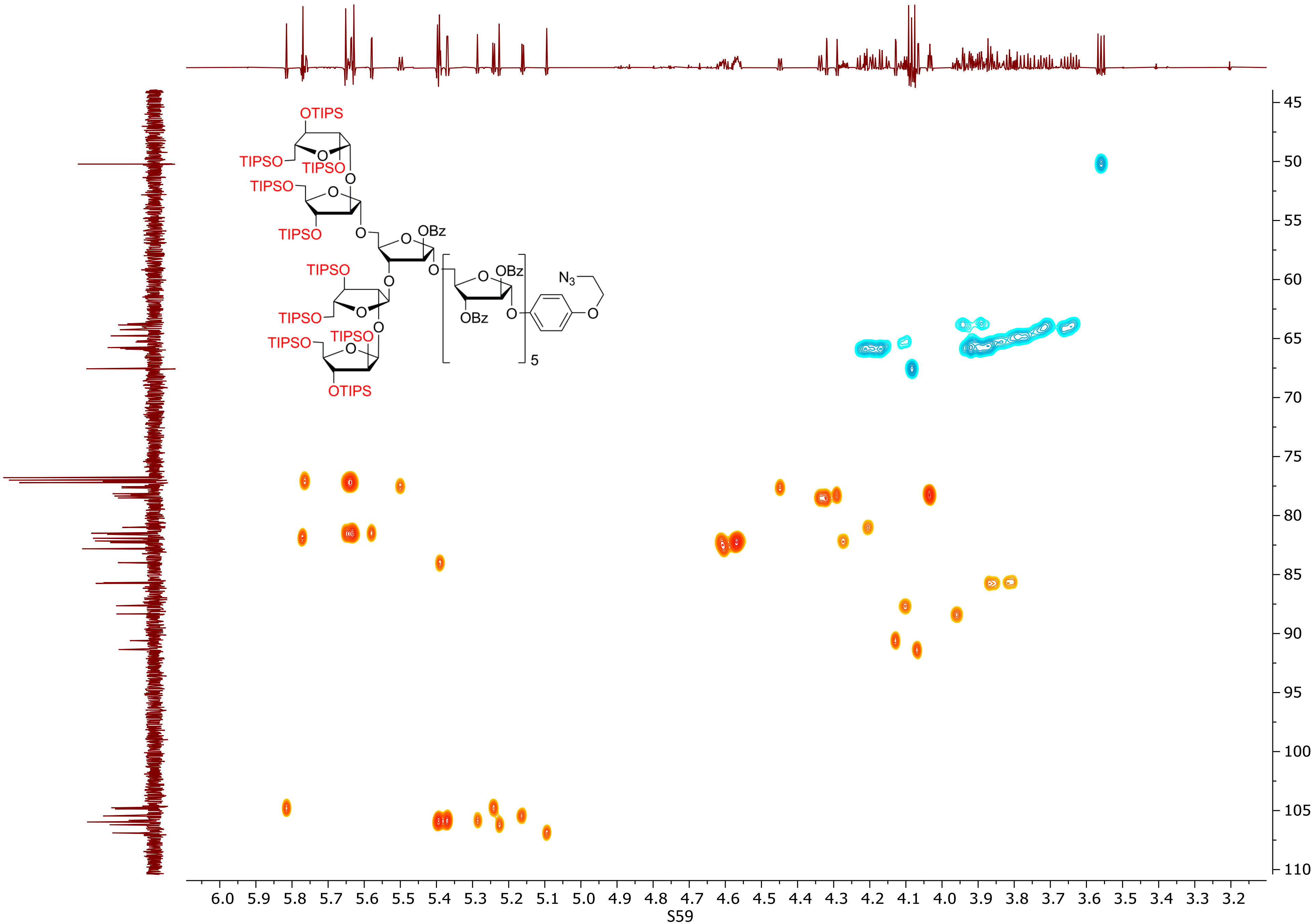

## $^{1}H$–$^{29}Si$ HMBC (300 MHz) spectrum of compound 19 in $CDCl_3$

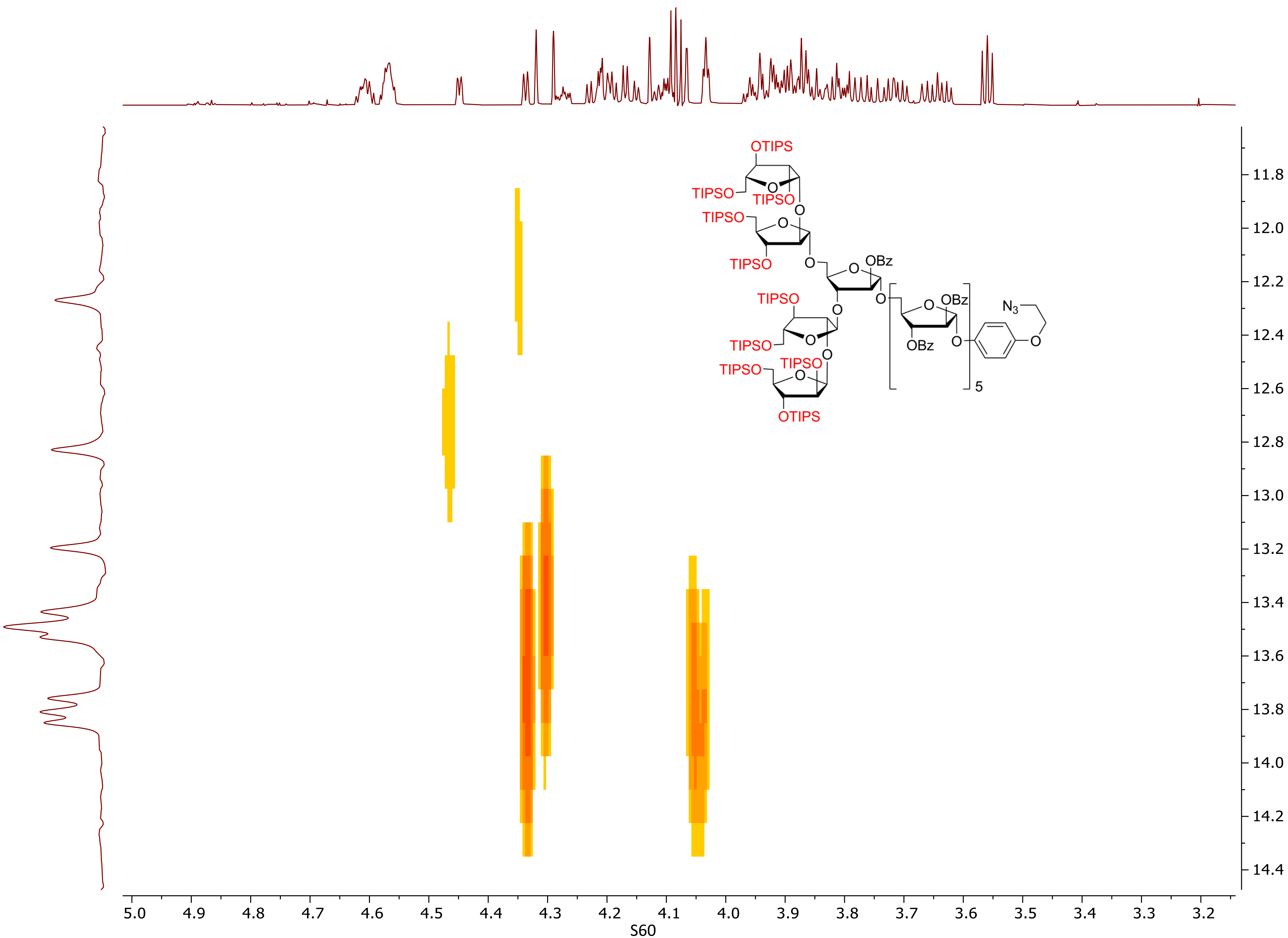

# $^{29}$Si INEPT NMR (59.6 MHz) spectrum of compound 19 in $CDCl_3$

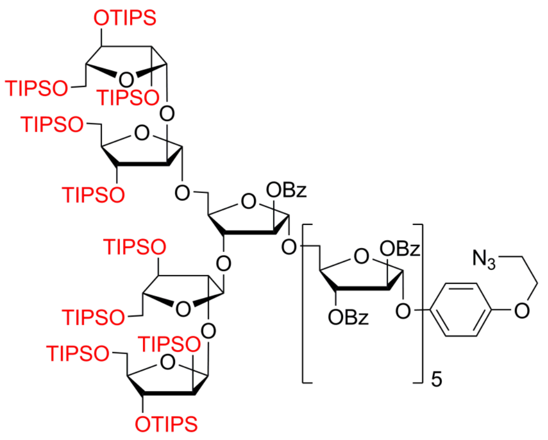


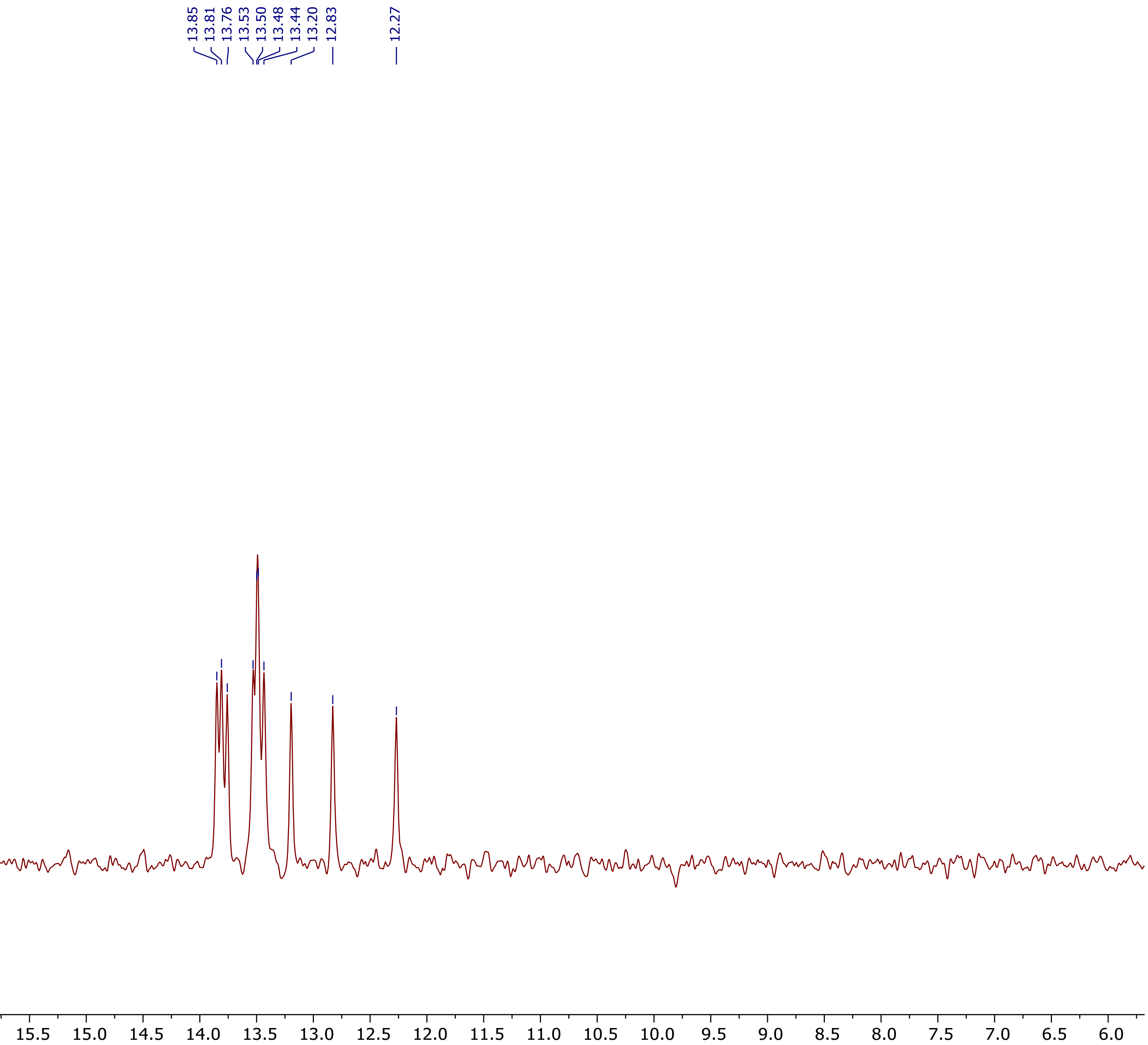

## $^1$H NMR (600 MHz) spectrum of compound 21 in $CDCl_3$

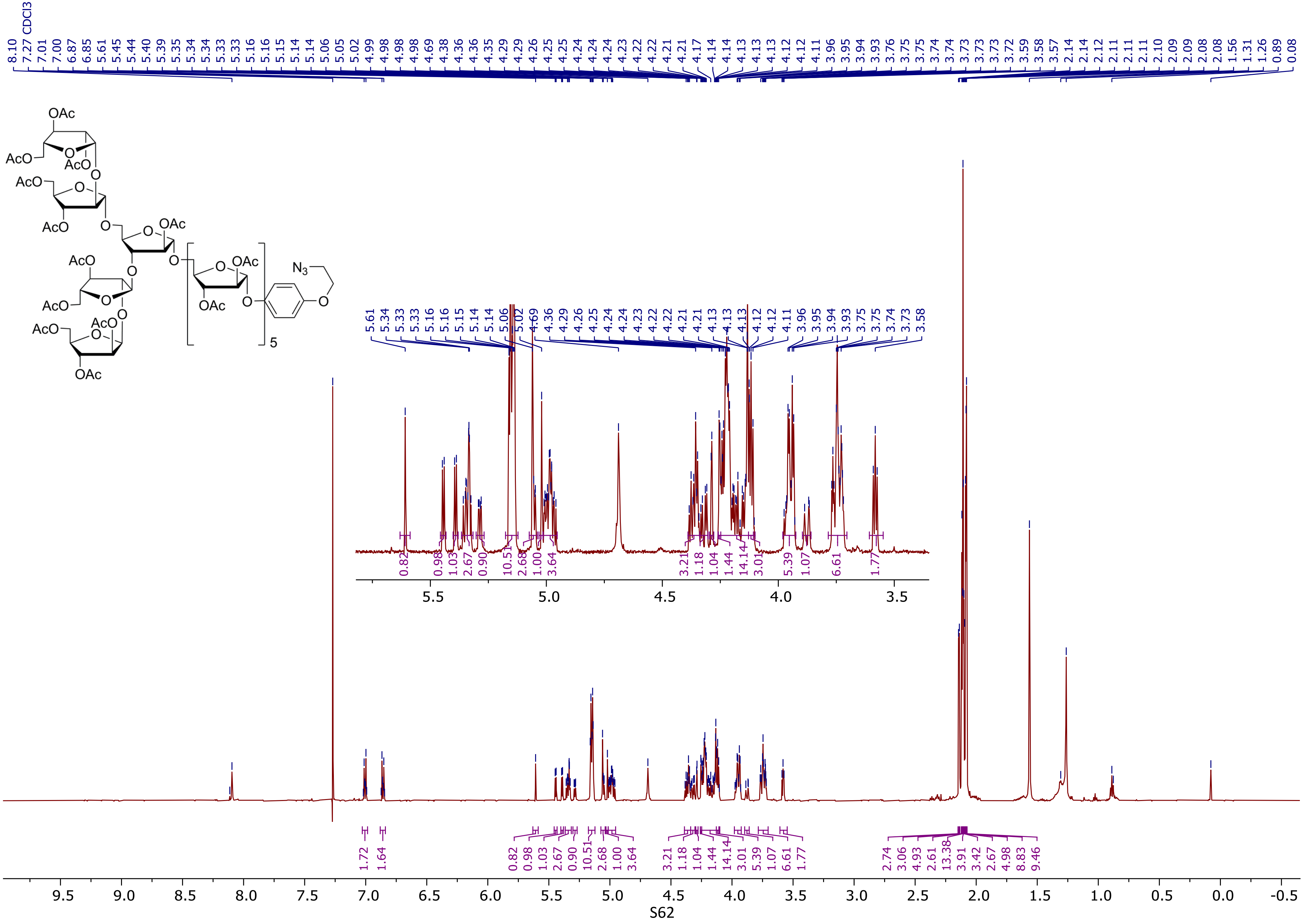

## $^{13}$C NMR (151 MHz) spectrum of compound 21 in $CDCl_3$

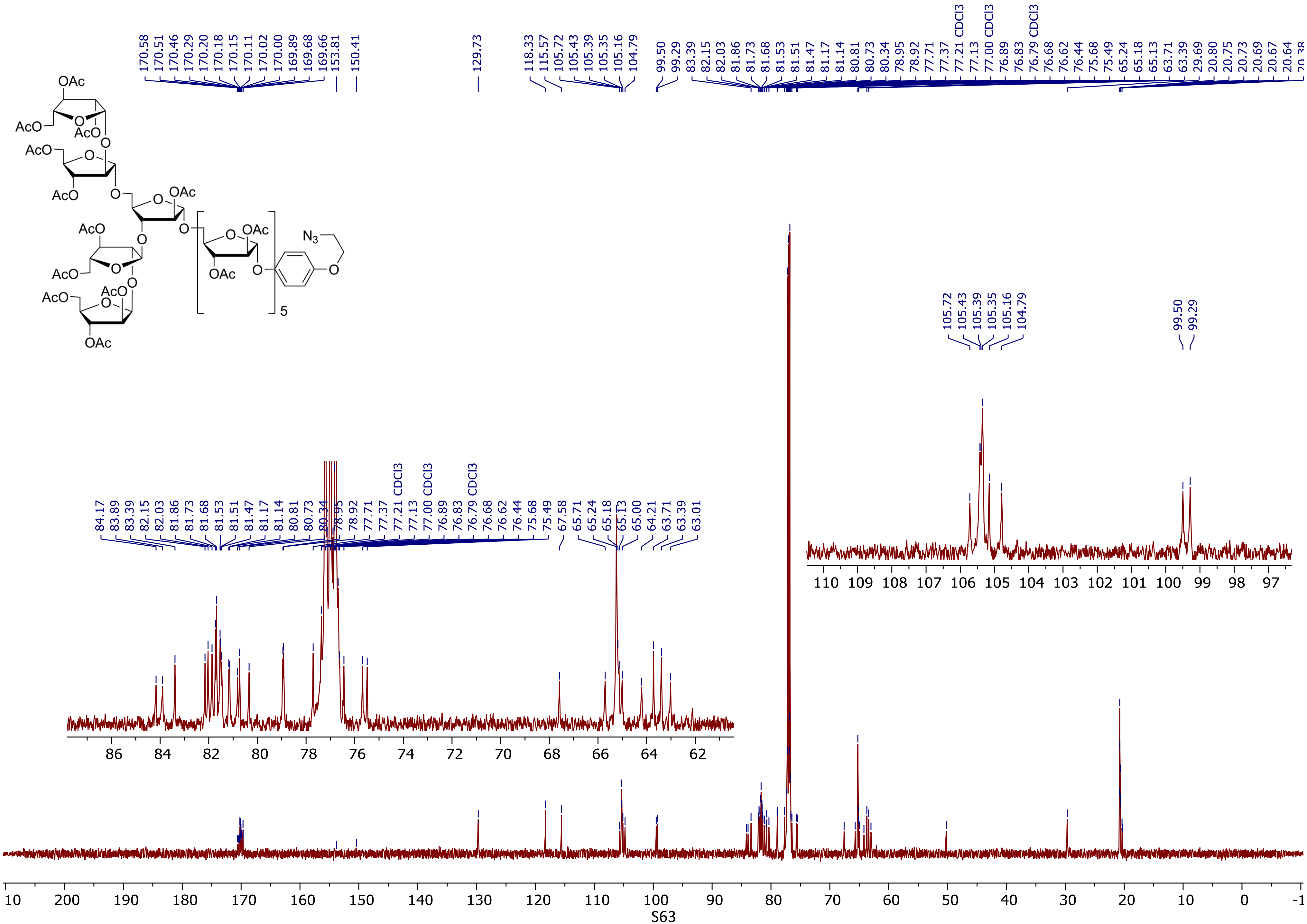

**COSY (600 MHz) spectrum of compound 21 in $CDCl_3$**

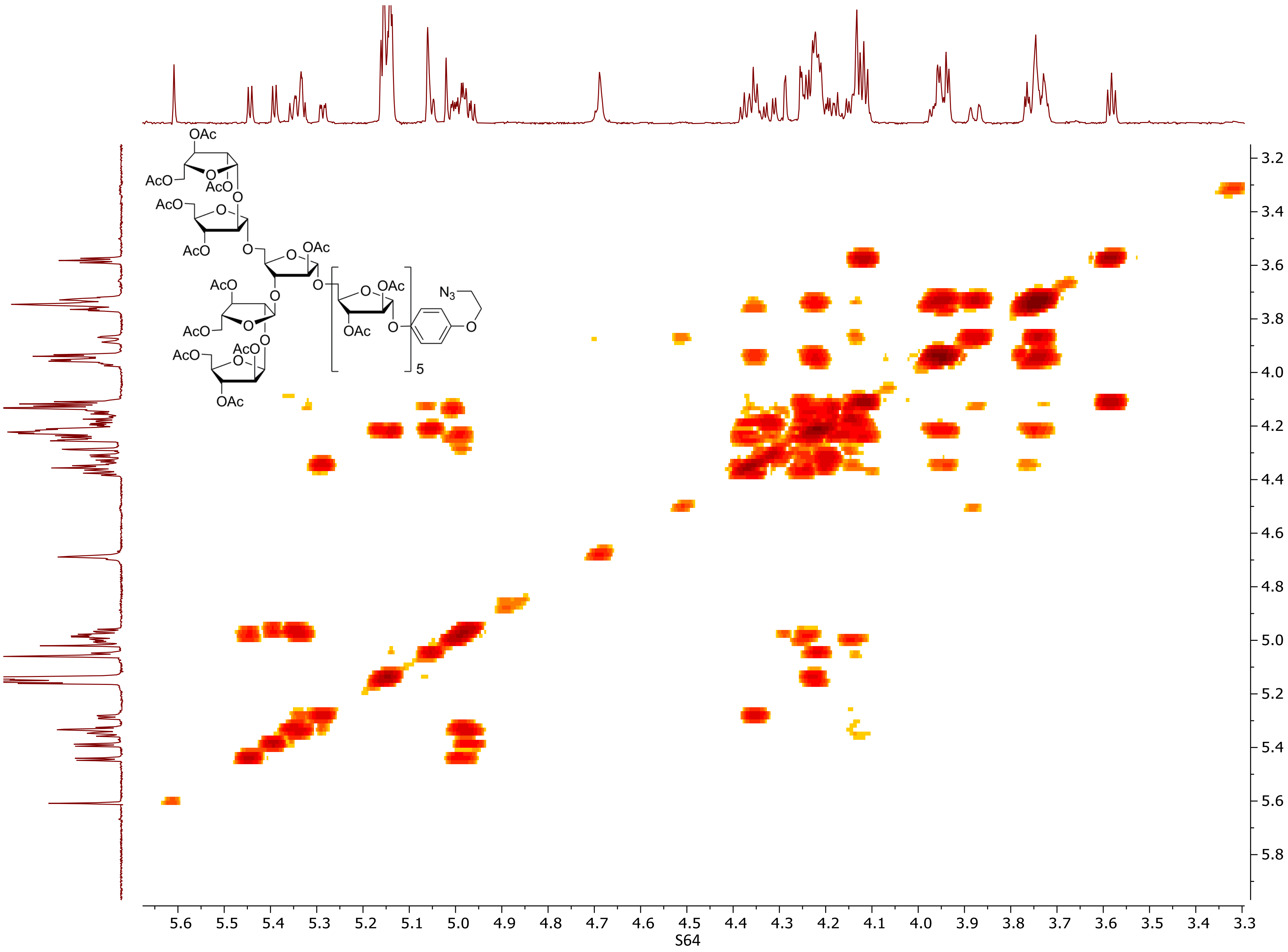

## HSQC (600 MHz) spectrum of compound 21 in $CDCl_3$

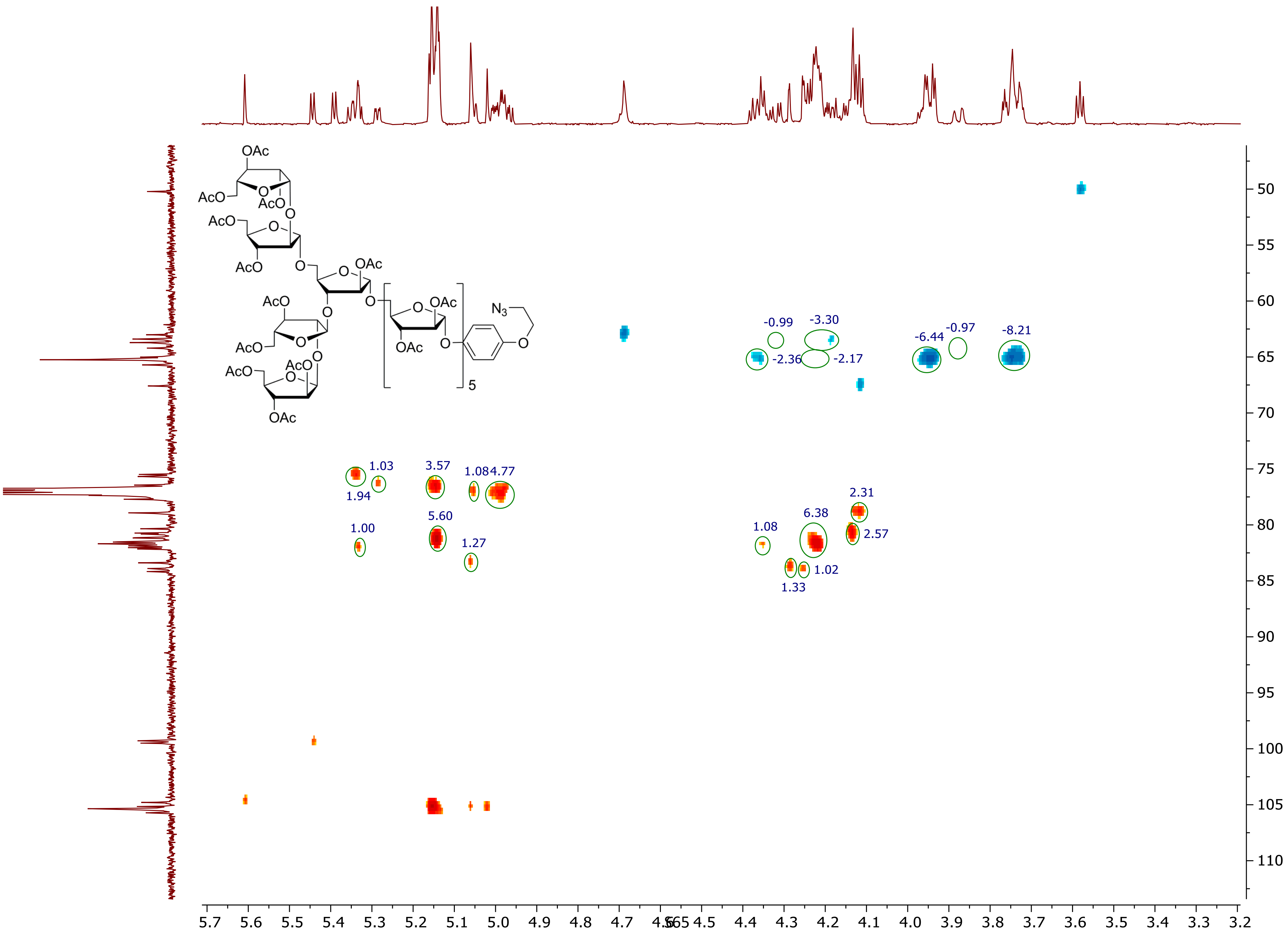

# HMBC (600 MHz) spectrum of compound 21 in $CDCl_3$

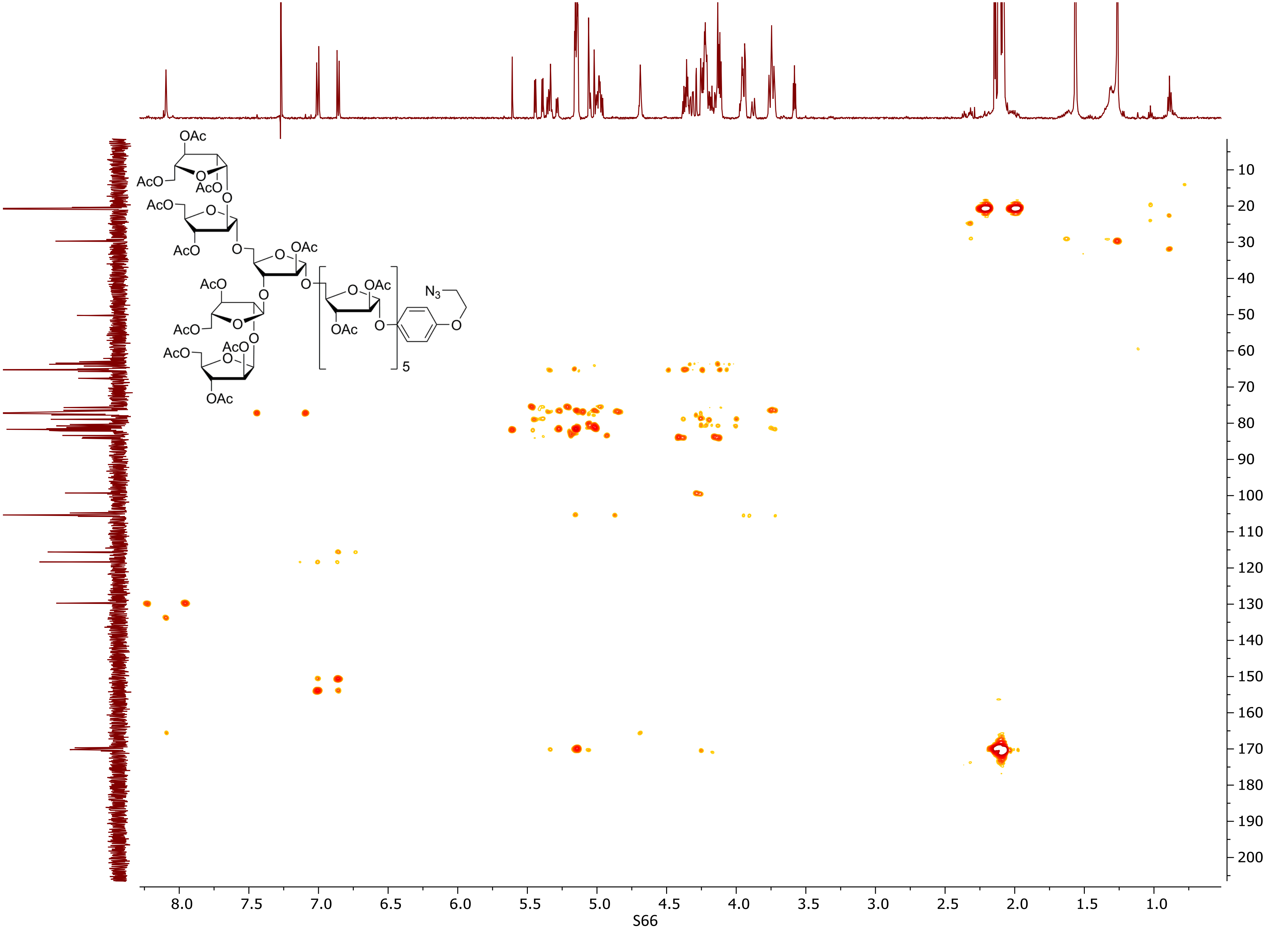

Supplement: Supplementary file 1 [file molecules-30-03295-s001.zip › molecules-3766976-supplementary.pdf]
